# Discovery of sparse, reliable omic biomarkers with Stabl

**DOI:** 10.1038/s41587-023-02033-x

**Published:** 2024-01-02

**Authors:** Julien Hédou, Ivana Marić, Grégoire Bellan, Jakob Einhaus, Dyani K. Gaudillière, Francois-Xavier Ladant, Franck Verdonk, Ina A. Stelzer, Dorien Feyaerts, Amy S. Tsai, Edward A. Ganio, Maximilian Sabayev, Joshua Gillard, Jonas Amar, Amelie Cambriel, Tomiko T. Oskotsky, Alennie Roldan, Jonathan L. Golob, Marina Sirota, Thomas A. Bonham, Masaki Sato, Maïgane Diop, Xavier Durand, Martin S. Angst, David K. Stevenson, Nima Aghaeepour, Andrea Montanari, Brice Gaudillière

**Affiliations:** 1 Department of Anesthesiology, Perioperative & Pain Medicine, Stanford University, Stanford, CA USA; 2 Department of Pediatrics, Stanford University, Stanford, CA USA; 3 Télécom Paris, Institut Polytechnique de Paris, Paris, France; 4 Department of Pathology and Neuropathology, University Hospital and Comprehensive Cancer Center Tübingen, Tübingen, Germany; 5 Division of Plastic and Reconstructive Surgery, Department of Surgery, Stanford University, Stanford, CA USA; 6 Department of Economics, Harvard University, Cambridge, MA USA; 7 Sorbonne University, GRC 29, AP-HP, DMU DREAM, Department of Anesthesiology and Intensive Care, Hôpital Saint-Antoine, Assistance Publique-Hôpitaux de Paris, Paris, France; 8 Department of Pathology, University of California San Diego, La Jolla, CA USA; 9 Department of Medical BioSciences, Radboud University Medical Center, Nijmegen, The Netherlands; 10 Bakar Computational Health Sciences Institute, University of California, San Francisco, San Francisco, CA USA; 11 Department of Medicine, University of Michigan Medical School, Ann Arbor, MI USA; 12 École Polytechnique, Institut Polytechnique de Paris, Paris, France; 13 Department of Biomedical Data Science, Stanford University, Stanford, CA USA; 14 Department of Statistics, Stanford University, Stanford, CA USA; 15 Department of Electrical Engineering, Stanford University, Stanford, CA USA

**Keywords:** Predictive medicine, Computational models

## Abstract

Adoption of high-content omic technologies in clinical studies, coupled with computational methods, has yielded an abundance of candidate biomarkers. However, translating such findings into bona fide clinical biomarkers remains challenging. To facilitate this process, we introduce Stabl, a general machine learning method that identifies a sparse, reliable set of biomarkers by integrating noise injection and a data-driven signal-to-noise threshold into multivariable predictive modeling. Evaluation of Stabl on synthetic datasets and five independent clinical studies demonstrates improved biomarker sparsity and reliability compared to commonly used sparsity-promoting regularization methods while maintaining predictive performance; it distills datasets containing 1,400–35,000 features down to 4–34 candidate biomarkers. Stabl extends to multi-omic integration tasks, enabling biological interpretation of complex predictive models, as it hones in on a shortlist of proteomic, metabolomic and cytometric events predicting labor onset, microbial biomarkers of pre-term birth and a pre-operative immune signature of post-surgical infections. Stabl is available at https://github.com/gregbellan/Stabl.

## Abstract (web summary)

Stabl selects sparse and reliable biomarker candidates from predictive models.

## Main

High-content omic technologies, such as transcriptomics, metabolomics or cytometric immunoassays, are increasingly employed in biomarker discovery studies^1,2^. These technologies allow researchers to measure thousands of molecular features in each biological specimen, offering unprecedented opportunities for advancing precision medicine tools across the spectrum of health and disease. Whether it is personalizing breast cancer diagnostics through multiplex imaging^3^ or identifying transcriptional signatures governing patient-specific vaccine responses across multiple vaccine types^4^, omic technologies have also dictated a shift in statistical analysis of biological data. The traditional univariate statistical framework is maladapted to large omic datasets characterized by a high number of molecular features *p* relative to the available samples *n*. The *p* ≫ *n* scenario reduces the statistical power of univariate analyses, and simply increasing *n* is often impractical due to cost or sample constraints^5,6^.

Statistical analysis in biomarker discovery research comprises three distinct tasks, all necessary for clinical translation and impacted by the *p* ≫ *n* challenge: (1) predicting clinical endpoints via identification of a multivariable model with high predictive performance (*predictivity*); (2) selecting a limited number of features as candidate clinical biomarkers (*sparsity*); and (3) ensuring confidence that the selected features are truly related to the outcome (*reliability*).

Several machine learning methods, including sparsity-promoting regularization methods (SRMs), such as Lasso^7^, Elastic Net (EN)^8^, Adaptive Lasso (AL)^9^ and sparse group Lasso (SGL)^10^, provide predictive modeling frameworks adapted to *p* ≫ *n* omic datasets. Furthermore, data fusion methods, such as early-fusion and late-fusion Lasso, enable integration of multiple, often heterogeneous, omic datasets^11,12^. Nevertheless, the challenge of selecting a sparse and reliable set of candidate biomarkers persists. Most SRMs employ *ℓ*_1_ regularization to limit the number of features in the final model. However, as the learning phase often relies on a limited number of samples, small perturbations in the training data can yield widely different sets of selected features^13–15^, undermining confidence in their relevance to the outcome. This inherent limitation hampers sparsity and reliability, impeding the biological interpretation and clinical significance of predictive models. Consequently, few omic biomarker discovery studies progress to later clinical development phases^1,2,5,6,16,17^.

High-dimensional feature selection methods, such as stability selection (SS), Model-X (MX) knockoff or bootstrap-enhanced Lasso (Bolasso), improve reliability by controlling for false discoveries in the selected feature set^18–20^. However, these methods often require a priori definition of the feature selection threshold or target false discovery rate (FDR), which decouples feature selection from the multivariable modeling process. Without prior knowledge of the data, this can lead to suboptimal feature selection, requiring multiple iterations to identify a desirable threshold and hindering optimal integration of multiple omic datasets into a unique predictive model, as a single fixed selection threshold may not be suited to the specificities of each dataset.

In this context, we introduce Stabl, a supervised machine learning framework designed to facilitate clinical translation of high-dimensional omic studies by bridging the gap between multivariable predictive modeling and the sparsity and reliability requirements of clinical biomarker discovery. Stabl combines noise injection into the original data, determination of a data-driven signal-to-noise threshold and integration of the selected features into a predictive model. Systematic benchmarking of Stabl against state-of-the-art SRMs, including Lasso, EN, SGL, AL and SS, using synthetic datasets, four existing real-world omic datasets and a newly generated multi-omic clinical dataset demonstrates that Stabl overcomes the shortcomings of current SRMs, thereby enhancing biological interpretation and clinical translation of sparse predictive models. The complete Stabl package is available at https://github.com/gregbellan/Stabl.

## Results

### Feature selection via false discovery proportion estimate

**Fig. 1:**
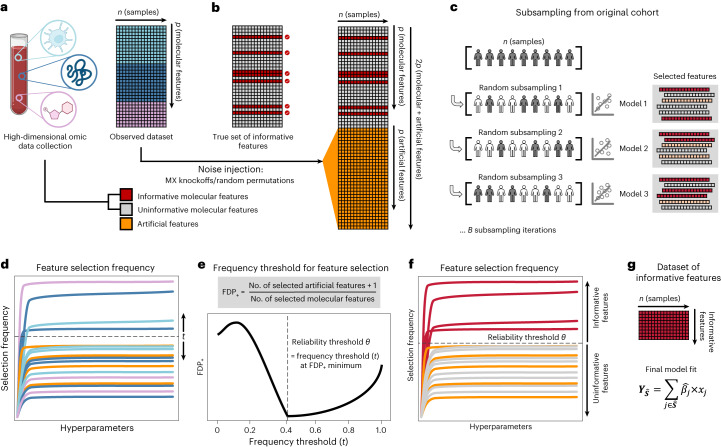
Overview of the Stabl algorithm. **a**, An original dataset of size *n* × *p* is obtained from measurement of *p* molecular features in each of *n* samples. **b**, Among the observed features, some are informative (related to the outcome, red), and others are uninformative (unrelated to the outcome, gray). *p* artificial features (orange), all uninformative by construction, are injected into the original dataset to obtain a new dataset of size *n* × 2*p*. Artificial features are constructed using MX knockoffs or random permutations. **c**, *B* subsample iterations are performed from the original cohort of size *n*. At each iteration *k*, SRM models varying in their regularization parameter(s) *λ* are fitted on the subsample, resulting in a different set of selected features for each iteration. **d**, For a given *λ*, *B* sets of selected features are generated in total. The proportion of sets in which feature *i* is present defines the feature selection frequency *f*_*i*_(*λ*). Plotting *f*_*i*_(*λ*) against 1/*λ* yields a stability path graph. Features whose maximum frequency is above a frequency threshold (*t*) are selected in the final model. **e**, Stabl uses the reliability threshold (*θ*), obtained by computing the minimum value of the FDP_+_ (Methods). **f**,**g**, The feature set with a selection frequency larger than *θ* (that is, reliable features) is included in a final predictive model.

**Extended Data Fig. 1:**
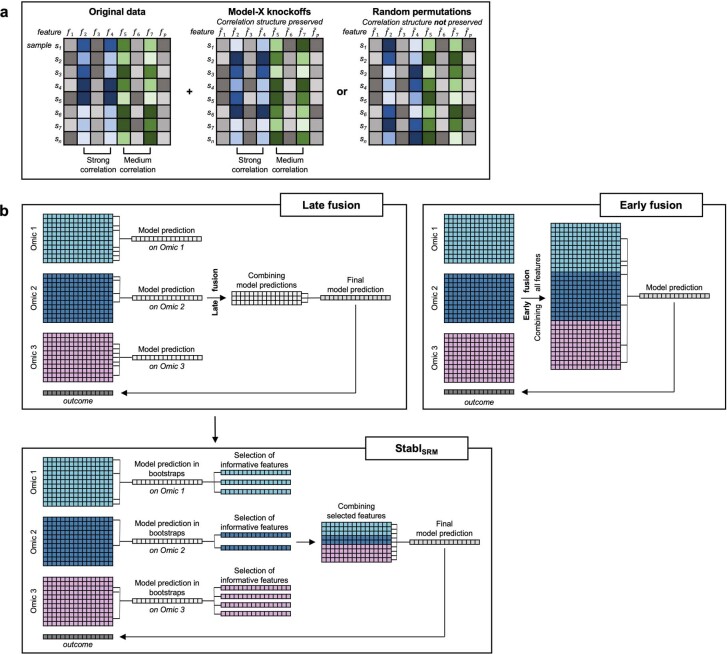
Infographics for noise injection methods and multi-omic data integration with StablSRM. **A**. **Noise injection methods**. Left panel depicting the original dataset with n samples and p features with strong correlation between features *f*! and *f*" as well as medium correlation between *f*# and *f*$. *Middle panel* showing MX knockoffs as noise injection method where generated artificial features preserve the original features’ correlation structure. *Right panel* showing random permutations as alternative noise generation method, which does not preserve the correlation structure. **B**. **Multi-omic data integration with StablSRM**. Early fusion approaches of multi-omic data integration combine all features of all omics to a concatenated dataset to derive a multivariate model. Late fusion approaches build predictive models on each omic layer individually, then concatenate the model predictions together and build a predictive model. StablSRM’s method builds models in a bootstrapping fashion on each omic individually to select the informative features, then concatenates all selected (informative) features and builds a final predictive model on all selected features.

When applied to a cohort randomly drawn from the population, SRMs will select informative features (that is, truly related to the outcome) with a higher probability, on average, than uninformative features (that is, unrelated to the outcome)^7,18^. However, as uninformative features typically outnumber informative features in high-dimensional omic datasets^1,2,17^, the fit of an SRM model on a single cohort can lead to selection of many uninformative features despite their lower probability of selection^18,20^. To address this challenge, Stabl implements the following strategy (Fig. 1 and Methods):

1. Stabl fits SRM models (Stabl_SRM_), such as Lasso, EN, SGL or AL, on subsamples of the data using a procedure similar to SS^18^. Subsampling mimics the availability of multiple random cohorts and estimates each feature’s selection frequency across all iterations. However, this procedure lacks an optimal frequency threshold for distinguishing informative from uninformative features objectively.
2. To define the optimal frequency threshold, Stabl creates artificial features unrelated to the outcome (noise injection) via MX knockoffs^19,21,22^ or random permutations^1–3^ (Extended Data Fig. 1), which we assume behave similarly to uninformative features in the original dataset^23^ (see ‘Theoretical guarantees’ in Methods). The artificial features are used to construct a false discovery proportion surrogate (FDP_+_). We define the ‘reliability threshold’, *θ*, as the frequency threshold that minimizes FDP_+_ across all possible thresholds. This method for determining *θ* is objective (minimizing a proxy for the FDP) and data driven (tailored to individual omic datasets).

As a result, Stabl provides a unifying procedure that selects features above the reliability threshold while building a multivariable predictive model. Stabl is amenable to both classification and regression tasks and can integrate multiple datasets of different dimensions and omic modalities. The complexity of the algorithm is described in Methods, and it allows for a scalable procedure with a runtime of under 1 h on a computer equipped with 32 vCPUs and 128 GB of RAM (Supplementary Table 1).

### Improved sparsity and reliability, retained predictivity

**Fig. 2:**
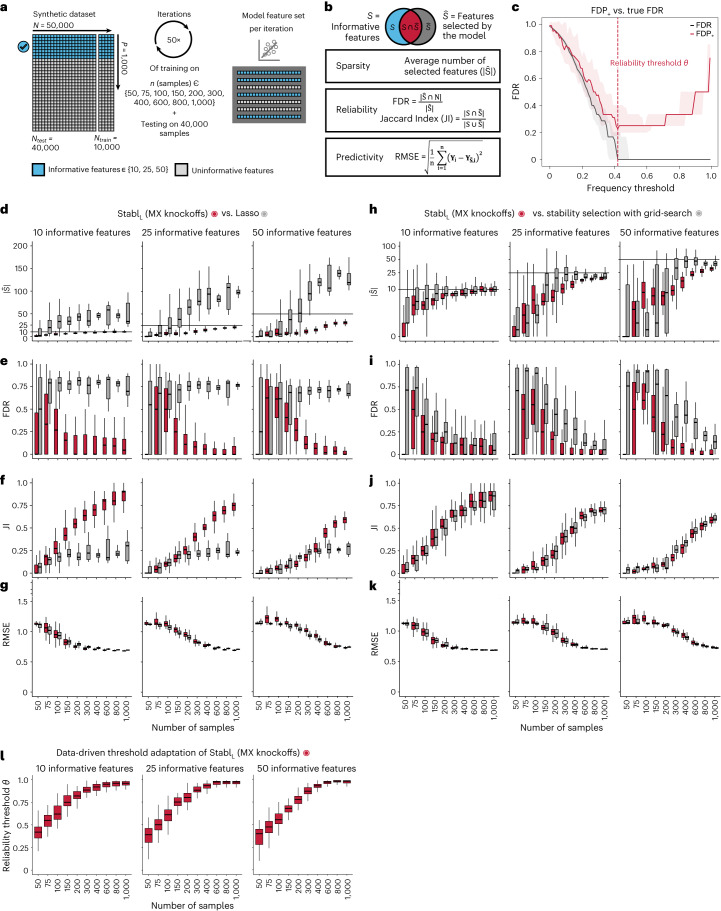
Synthetic dataset benchmarking against Lasso. **a**, A synthetic dataset consisting of *n* = 50,000 samples × *p* = 1,000 normally distributed features was generated. Some features are correlated with the outcome (informative features, light blue), whereas the others are not (uninformative features, gray). Forty thousand samples are held out for validation. Out of the remaining 10,000, 50 sets of sample sizes *n* ranging from 50 to 1,000 are drawn randomly to assess model performance. The Stabl_SRM_ framework is used using Lasso (Stabl_L_) with MX knockoffs for noise generation. Performances are tested on continuous outcomes (regression tasks). **b**, Sparsity (average number of selected features, $| \hat{S}|$), reliability (true FDR and JI) and predictivity (RMSE) metrics used for performance evaluation. **c**, The FDP_+_ (red line; 95% CI, red shading) and the true FDR (gray line; 95% CI, gray shading) as a function of the frequency threshold (example shown for *n* = 150 samples and 25 informative features; see Extended Data Fig. 3 for other conditions). The FDP_+_ estimate approaches the true FDR around the reliability threshold, *θ*. **d**–**g**, Sparsity (**d**), reliability (FDR, **e**; JI, **f**) and predictivity (RMSE, **g**) performances of Stabl_L_ (red box plots) and Lasso (gray box plots) with increasing number of samples (*n*, *x* axis) for 10 (left panels), 25 (middle panels) or 50 (right panels) informative features. **h**–**k**, Sparsity (**h**), reliability (**i** and **j**) and predictivity (**k**) performances of models built using a data-driven reliability threshold *θ* (Stabl_L_, red box plots) or grid search-coupled SS (gray box plots). **l**, The reliability threshold chosen by Stabl_L_ shown as a function of the sample size (*n*, *x* axis) for 10 (left panel), 25 (middle panel) or 50 (right panel) informative features. Boxes indicate median and IQR; whiskers indicate 1.5× IQR.

We benchmarked Stabl using synthetic training and validation datasets containing known informative and uninformative features (Fig. 2a). Simulations mimicking real-world scenarios incorporated variations in sample size (*n*), number of total features (*p*) and informative features (∣*S*∣). Three key performance metrics were employed (Fig. 2b and Supplementary Table 2):

1. *Sparsity*: measured as the average number of selected features ($| \hat{S}|$) relative to informative features
2. *Reliability*: evaluated through the FDR and Jaccard index (JI), indicating the overlap between algorithm-selected features and true informative features
3. *Predictivity*: assessed using root mean square error (RMSE)

**Extended Data Fig. 2:**
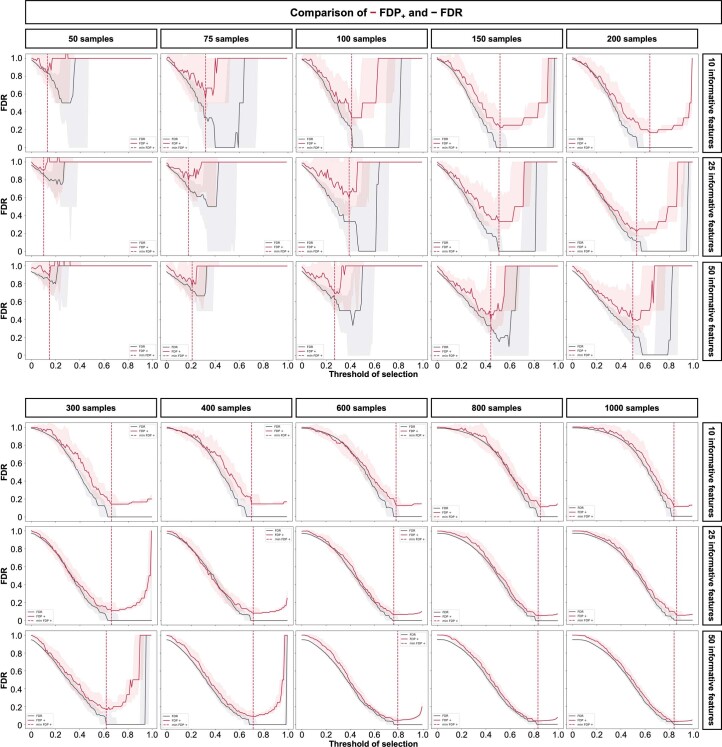
Comparison of FDP_+_ and *FDR* in synthetic dataset benchmarking. On the generated synthetic dataset, the FDP_+_ and the true FDR were assessed for different dataset sizes ranging from n = 50 to 1000 samples with 10 (*upper panels*), 25 (*middle panels*), or 50 (*lower panels*) informative features. The FDP_+_ (*red line*) and the true FDR (*black line*) are shown as a function of the frequency threshold. The selected reliability threshold (*θ*, *red dotted line*) varied across conditions. Boxes in box plots indicate the median and interquartile range (IQR), with whiskers indicating 1.5 × IQR.

**Extended Data Fig. 3:**
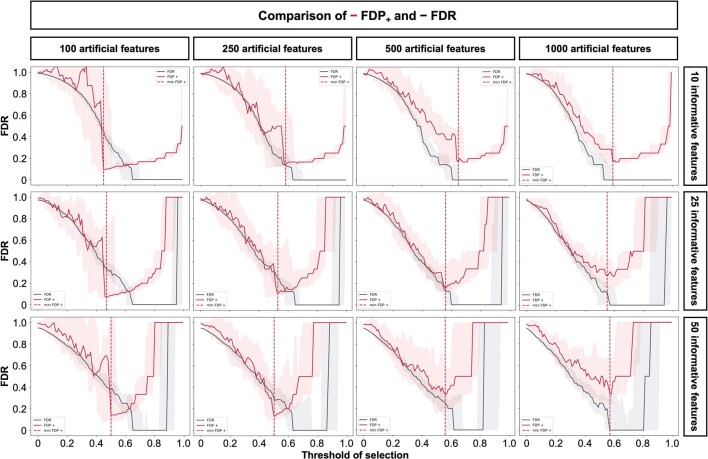
Effect of varying numbers of artificial features on the computation of FDP_+_. On the generated synthetic dataset, the FDP_+_ and the true FDR were assessed for a varying number of artificial features on a dataset of n = 200 samples and 10 (*upper panels*), 25 (*middle panels*), or 50 (*lower panels*) informative features within p = 1000 features. The FDP_+_ (*red line*) and the true FDR (*black line*) are shown as a function of the frequency threshold. Increasing the number of artificial features allows for a more accurate estimation of the reliability threshold (*θ*, *red dotted line*). Boxes in box plots indicate the median and interquartile range (IQR), with whiskers indicating 1.5 × IQR.

Before benchmarking, we tested whether Stabl’s FDP_+_ experimentally controls the FDR at the reliability threshold *θ*, as the actual FDR value is known for synthetic data. We observed that FDP_+_(*θ*) consistently exceeded the true FDR value (Fig. 2c and Extended Data Fig. 2). Further experiments explored how the number of artificial features influenced FDP_+_ computation. Results indicated that increasing artificial features improved FDP_+_(*θ*) estimation, notably with more than 500 artificial features (Extended Data Fig. 3). These observations experimentally confirmed Stabl’s validity in optimizing the frequency threshold for feature selection. Furthermore, under the assumption of feature exchangeability between uninformative and artificial features, we bound the probability that FDP exceeds a multiple of the proximity to FDP_+_(*θ*), thus providing a theoretical validation of our experimental observations (see ‘Theoretical guarantee’ in Methods).

#### Benchmarking against Lasso and SS

**Extended Data Fig. 4:**
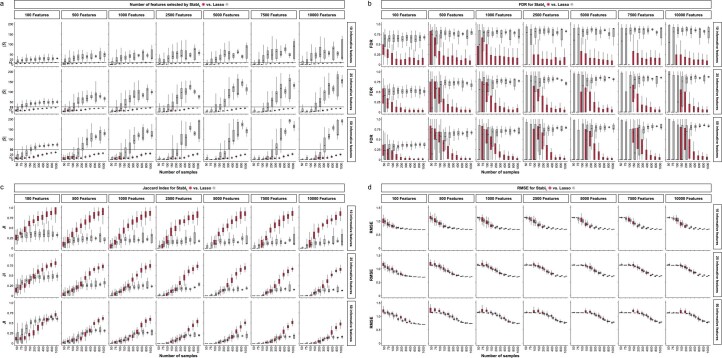
Stabl_L_’s performance on synthetic data with varying number of total features compared to Lasso. Synthetic datasets differing in the number of features were generated as described in Fig. 2. Sparsity (∣Ŝ∣, **a**) reliability (FDR, **b**, and JI, **c**), and predictivity (RMSE, **d**) of Stabl_L_ (*red box plots*) and Lasso (*grey box plots*) as a function of the number of samples (n, x-axis) for 10 (*left*), 25 (*middle*), or 50 (*right*) informative features within p = 100, 500, 1000, 2500, 5000, 7500, and 10000 total number of features. Boxes in box plots indicate the median and interquartile range (IQR), with whiskers indicating 1.5 × IQR.

**Extended Data Fig. 5:**
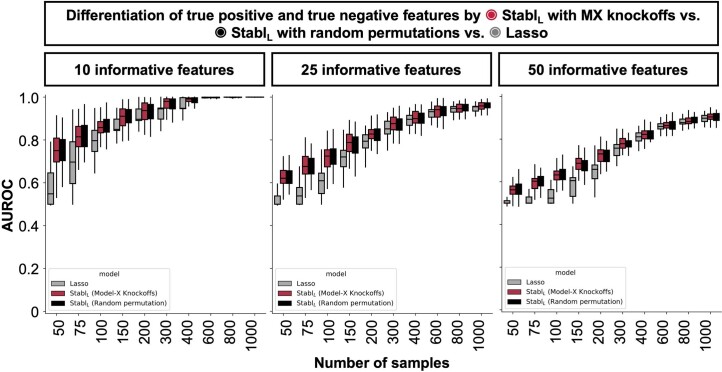
Reliability performance of selection frequency (Stabl_L_) and beta coefficients (Lasso) to distinguish true positive and true negative features. Beta coefficients assigned by Lasso and feature selection frequency assigned by Stabl were used to distinguish true positive and true negative features in a synthetic dataset with p = 1000 total features. The AUROC for this procedure is shown as a function of the number of samples (n, x-axis) for 10 (*left panels*), 25 (*middle panels*), or 50 (right panels) informative features. Boxes in box plots indicate the median and interquartile range (IQR), with whiskers indicating 1.5 × IQR.

Stabl_SRM_ was first benchmarked against Lasso using normally distributed, uncorrelated data for regression tasks, incorporating MX knockoffs as artificial features (Fig. 2d–g and Extended Data Fig. 4). Stabl_L_ consistently achieved greater sparsity compared to Lasso by selecting fewer features across all conditions tested, converging toward the true number of informative features (Fig. 2d). Stabl_L_ also achieved better reliability compared to Lasso, as evidenced by lower FDR (Fig. 2e) and higher JI (increased overlap with the true informative feature set) (Fig. 2f). Moreover, Stabl_L_’s feature selection frequency better distinguished true positives from true negatives, enhancing accuracy, as measured by the area under the receiver operating characteristic (AUROC) curve, compared to Lasso coefficients, thus providing an additional metric for estimating reliability (Extended Data Fig. 5). Notably, Stabl_L_ and Lasso exhibited similar predictivity (Fig. 2g).

**Extended Data Fig. 6:**
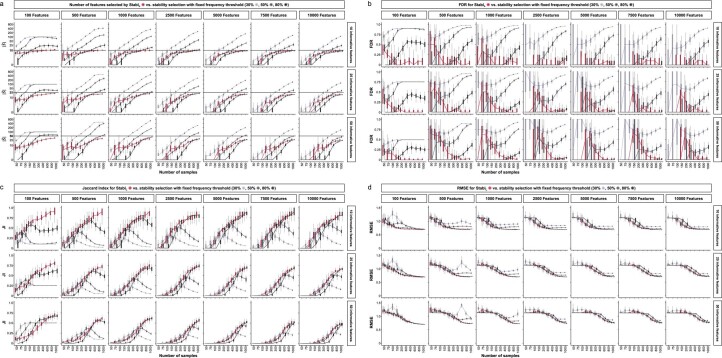
Stabl_L_’s performance on synthetic data with varying number of total features compared to SS with fixed frequency thresholds. Synthetic datasets differing in the number of total features were generated as described in Fig. 2. Sparsity (∣Ŝ∣, **a**), reliability (FDR, **b**, and JI, **c**), and predictivity (RMSE, **d**) of Stabl_L_ (*red lines*) and stability selection with fixed frequency threshold of 30% (*light grey lines*), 50% (*dark grey lines*), or 80% (*black lines*) as a function of the number of samples (n, x-axis) for 10 (*left*), 25 (*middle*), or 50 (*right*) informative features within p = 100, 500, 1000, 2500, 5000, 7500, and 10000 total number of features. Boxes in box plots indicate the median and interquartile range (IQR), with whiskers indicating 1.5 × IQR.

We then assessed the impact of data-driven *θ* computation in comparison to SS, which relies on a fixed frequency threshold chosen a priori. Three representative frequency thresholds were evaluated: 30%, 50% or 80% (Extended Data Fig. 6). The choice of threshold greatly affected model performance depending on the simulation conditions: the 30% threshold yielded the highest sparsity and reliability with smaller sample sizes (*n* < 75), whereas the 80% threshold resulted in superior performances with larger sample sizes (*n* > 500). In contrast, Stabl_L_ systematically reached optimal sparsity, reliability and predictivity. To generalize the comparative analysis of SS and Stabl_L_, we coupled SS with a grid search method to find the optimal feature selection threshold (Fig. 2h–k). The analysis demonstrated that the grid search-coupled SS method produced models with more features and greater variability in feature selection compared to Stabl_L_. Furthermore, Stabl_L_ consistently improved reliability (lower FDR) at similar predictive performance compared to the grid search-coupled SS method. We also show that Stabl_L_’s *θ* varied greatly with sample size (Fig. 2l), illustrating its adaptive ability to identify an optimal frequency threshold solution across datasets of different dimensions.

#### Extension of Stabl_SRM_ to multi-omic synthetic datasets

Finally, experiments were performed simulating integration of multiple omic datasets. Unlike the early-fusion method, which concatenates all omic data layers before applying a statistical learner, Stabl adopts an independent analysis approach, fitting specific reliability thresholds for each omic data layer before selecting the most reliable features to merge into a final layer. Consequently, Stabl_L_ was benchmarked against Lasso using the comparable late-fusion method, wherein a model is trained on each omic dataset independently before merging the predictions into a final dataset (Extended Data Fig. 1)^11,12^. The results show that Stabl_L_ improved the sparsity and reliability of integrated multi-omic models compared to late-fusion Lasso at a similar predictive performance (Supplementary Table 3).

In sum, synthetic modeling results show that Stabl_L_ achieves better sparsity and reliability compared to Lasso while preserving predictivity and that Stabl_L_’s feature selection aligns more closely with the true set of informative features. These findings underscore the advantage of data-driven adaptation of the frequency threshold to each dataset’s unique characteristics, as opposed to relying on arbitrarily pre-determined thresholds.

### Generalization to other sparse learners and distributions

**Fig. 3:**
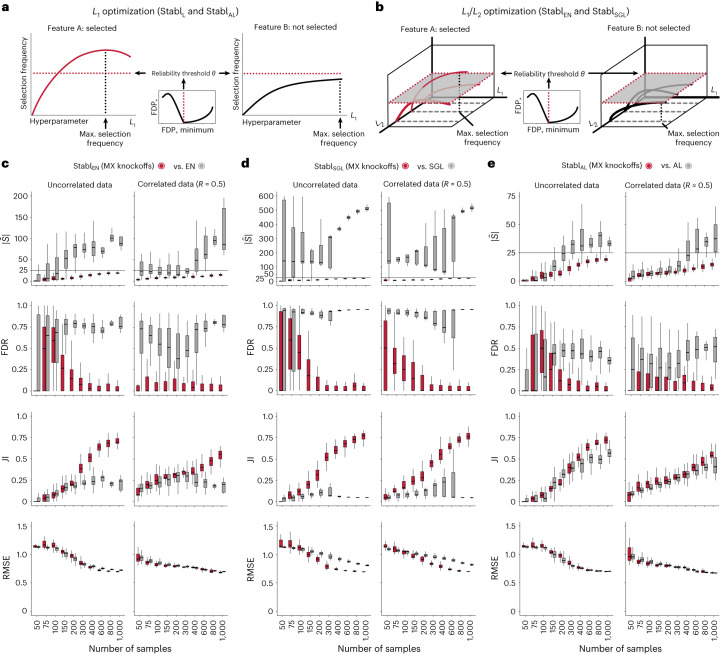
Extension of the Stabl_SRM_ framework to EN, SGL and AL: synthetic dataset benchmarking. The Stabl_SRM_ framework is benchmarked against various SRMs, including EN (Stabl_EN_), SGL (Stabl_SGL_) and AL (Stabl_AL_), respectively. **a**,**b**, Diagrams depict the strategy for identifying the maximum selection frequency for each feature across one (*L*_1_ for Lasso and AL, **a**) or two (*L*_1_/*L*_2_ for EN and SGL, **b**) regularization parameters before minimizing the FDP_+_. **c**–**e**, Sparsity ($| \hat{S}|$), reliability (FDR and JI) and predictivity (RMSE) performances of Stabl_SRM_ (red box plots) are compared to their respective SRM (gray box plots) in *n* = 50 independent experiments for each number of samples for Stabl_EN_ (**c**), Stabl_SGL_ (**d**) and Stabl_AL_ (**e**). Synthetic modeling experiments performed on normally distributed datasets containing *S* = 25 informative features with uncorrelated (left panels) or intermediate correlation structures (right panels) are shown. For all correlated datasets, the target correlation between informative features is set at a Pearson correlation coefficient, *R*, of 0.5, yielding a covariance matrix with approximately the target correlation (*R* ≈ 0.5). Results with low or high correlation structures are shown in Extended Data Fig. 7. Performances are shown for regression tasks. Results for classification tasks are shown in Supplementary Table 10. Box plots indicate median and IQR; whiskers indicate 1.5× IQR.

**Extended Data Fig. 7:**
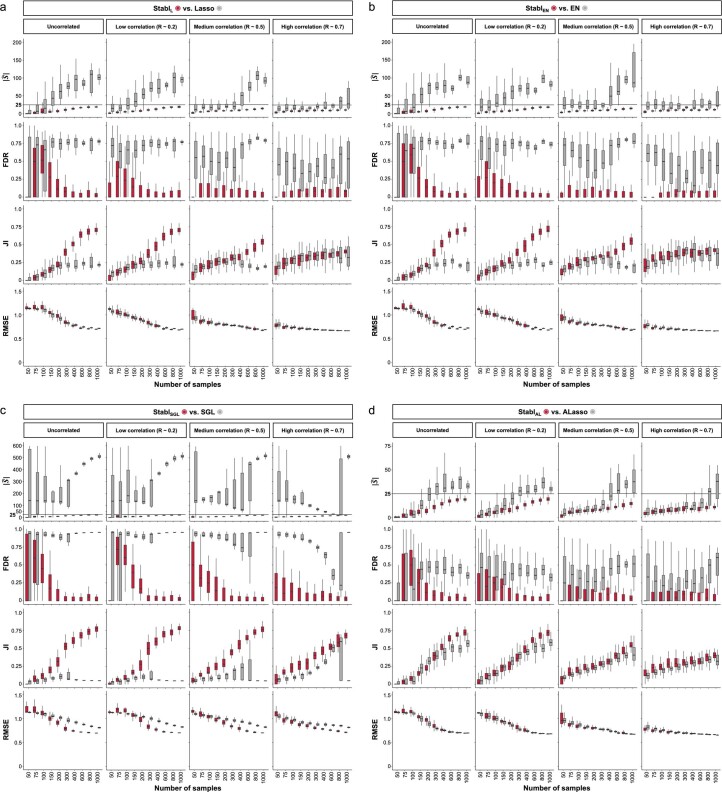
Stabl’s performance on synthetic data with different correlation structures. Synthetic datasets differing in correlation structure (low, medium, or high) were generated as described in Fig. 3. Sparsity (∣Ŝ∣, *upper panels*), reliability (FDR and JI, *middle panels*), and predictivity performances (AUROC, lower panels) for Stabl_L_ (**a**), Stabl_EN_ (**b**), Stabl_SGL_ (**c**), and Stabl_AL_ (**d**) (*red box plots*) and Lasso (*grey box plots*) as a function of the number of samples (n, x-axis) for 10 (*left panels*), 25 (*middle panels*), or 50 (right panels) informative features. Boxes in box plots indicate the median and interquartile range (IQR), with whiskers indicating 1.5 × IQR.

**Extended Data Fig. 8:**
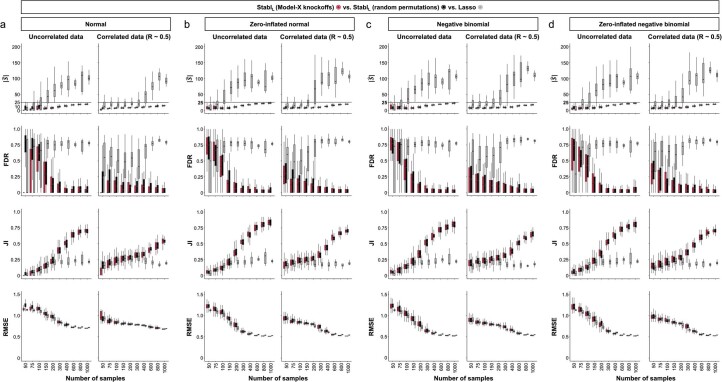
Stabl_L_’s performance with MX knockoffs or random permutations on synthetic data with normal and non-normal distributions compared to Lasso. Synthetic datasets differing in distribution were generated using the Normal to Anything (NORTA) framework, as described in methods. Sparsity (∣Ŝ∣, *upper panels*), reliability (FDR and JI, *middle panels*), and predictivity performances (RMSE, *lower panels*) of Stabl_L_ (MX knockoffs, *red box plots*, or random permutations, black box plots), and Lasso (grey box plots) as a function of the number of samples (n, x-axis) for synthetic data with a normal distribution (**a**), zero-inflated normal distribution (**b**), negative binomial distribution (**c**), or zero-inflated negative binomial distribution (**d**). The results are shown for datasets with 25 informative features in the context of uncorrelated (*left panels*) or correlated (*right panels*, intermediate correlation, R ~ 0.5) data for regression tasks (continuous outcomes). Results obtained for other scenarios, including other SRMs (EN, SGL, and AL), correlation structures (low, R ~ 0.2, high, R ~ 0.7), and classification tasks are listed in Table S2. Boxes in box plots indicate the median and interquartile range (IQR), with whiskers indicating 1.5 × IQR.

**Extended Data Fig. 9:**
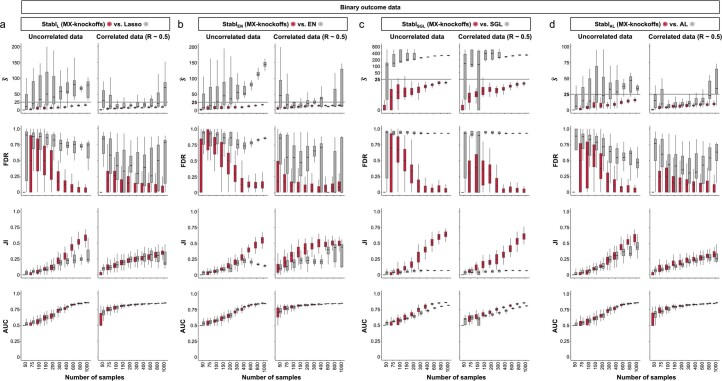
Stabl’s performance on synthetic data with binary outcomes. Synthetic datasets with binary outcome variables were generated as described in Fig. 3. Sparsity (∣Ŝ∣), reliability (FDR and JI), and predictivity (RMSE) performances of StablSRM (*red box plots*) compared to the respective SRM (*grey box plots*) as a function of the sample size (n, x-axis) for Stabl_L_ (**a**), Stabl_EN_ (**b**), Stabl_SGL_ (**c**), and Stabl_AL_ (**d**). Scenarios with 25 informative features and uncorrelated (*left panels*) or intermediate feature correlation structures (Spearman R ~ 0.5, *right panels*) are shown. Boxes in box plots indicate the median and interquartile range (IQR), with whiskers indicating 1.5 × IQR.

A notable benefit of Stabl is the modularity of the statistical framework, enabling the use of different SRMs as base learners and different noise generation techniques (Methods). This modularity enables customization for datasets with various correlation structures, where specific SRMs may outperform Lasso. We conducted synthetic modeling experiments comparing SRM substitutions within the Stabl_SRM_ framework to their cognate SRM, including EN, SGL or AL (Fig. 3 and Extended Data Fig. 7). We also explored different feature distributions (normal, zero-inflated normal, negative binomial and zero-inflated negative binomial; Methods and Extended Data Fig. 8) and prediction tasks (regression (Fig. 3 and Extended Data Fig. 7) and classification (Extended Data Fig. 9 and Supplementary Table 2)). Synthetic datasets with *S* = 25 informative features, *p* = 1,000 total features and *n* ranging from 50 to 1,000 samples were used for these experiments.

Lasso encounters challenges with correlated data structures^9,24^, often favoring one of two correlated covariates. EN mitigates this by introducing *ℓ*_2_ regularization, encouraging consideration of multiple correlated features. Similarly, SGL handles correlated data with known groupings or clusters, by introducing a combination of between-group and within-group sparsity.

To integrate SRMs with multiple regularization hyperparameters (for example, *ℓ*_1_/*ℓ*_2_ for EN and SGL), Stabl_SRM_ extends the identification of the maximum selection frequency of each feature to a multi-dimensional space (Fig. 3a,b and Methods). Further simulation experiments benchmarked Stabl_EN_ against EN across low (*R* ≈ 0.2), intermediate (*R* ≈ 0.5) and high (*R* ≈ 0.7) Spearman correlations and Stabl_SGL_ against SGL in datasets containing known groups of correlated features (defined in Methods). Here, MX knockoff was used as it preserves the correlation structure of the original dataset (Extended Data Fig. 1)^25^. For low or intermediate correlation structures, Stabl_EN_ and Stabl_SGL_ selected fewer features with improved JI and FDR and similar predictivity compared to EN or SGL (Fig. 3c,d and Extended Data Fig. 7). In highly correlated datasets (Extended Data Fig. 7), the JI for Stabl_EN_ and Stabl_SGL_ paralleled that of EN and SGL, respectively, but with lower FDR across all correlation levels. This suggests that, whereas EN or SGL may achieve a similar JI to Stabl_EN_ or Stabl_SGL_, they do so at the expense of selecting more uninformative features.

Other SRMs offer advantages beyond adapting to different correlation structures. For example, AL, an extension of Lasso that demonstrates the oracle property^9^, ensures accurate identification of informative features as the sample size approaches infinity. Compared to AL, integrating AL within the Stabl framework (Stabl_AL_) resulted in fewer selected features, lower FDR and overall improved JI, especially evident with increasing sample sizes (Fig. 3e and Extended Data Fig. 7). For experiments with normally distributed, uncorrelated data, although AL had a higher JI compared to Stabl_AL_ in two out of 10 cases (sample sizes *n* = 150 and *n* = 200), Stabl_AL_ exhibited lower FDR for these sample sizes and beyond. These findings indicate that Stabl_AL_ improves the selection of informative features compared to AL, offering an advantageous approach, especially in the context of biomarker discovery studies with large sample sizes.

### Stabl enables biomarker discovery in omic studies

We evaluated Stabl’s performance on five distinct clinical omic datasets, encompassing various dimensions, signal-to-noise ratios, data structures, technology-specific pre-processing and predictive performances. Four were previously published with standard SRM analyses, whereas the fifth is a newly generated dataset. These datasets spanned bulk and single-cell omic technologies, including RNA sequencing (RNA-seq) (comprising cell-free RNA (cfRNA) and microbiome datasets), high-content proteomics, untargeted metabolomics and single-cell mass cytometry. To ensure broad applicability, we tested different Stabl_SRM_ variations using three base SRMs (Lasso, EN and AL) benchmarked against their respective SRM. To preserve the original data’s correlation structure, we primarily employed MX knockoffs for introducing noise across all omic datasets, except for the cfRNA dataset. This dataset exhibited the lowest internal correlation levels (with <1% of features displaying intermediate correlations, *R* > 0.5; Supplementary Table 4), prompting the use of random permutation as the noise generation approach.

In contrast to synthetic datasets, the true set of informative features is unknown in real-world datasets, precluding an assessment of true reliability performance. Consequently, we employed distinct performance metrics:

1. *Sparsity*: representing the average number of features selected throughout the cross-validation (CV) procedure
2. *Predictivity*: assessed through the AUROC for classification tasks or the RMSE for regression tasks

Model performances were evaluated over 100 random repetitions using a repeated five-fold or Monte Carlo CV strategy.

#### Sparse, reliable biomarker discovery from single-omic data

**Fig. 4:**
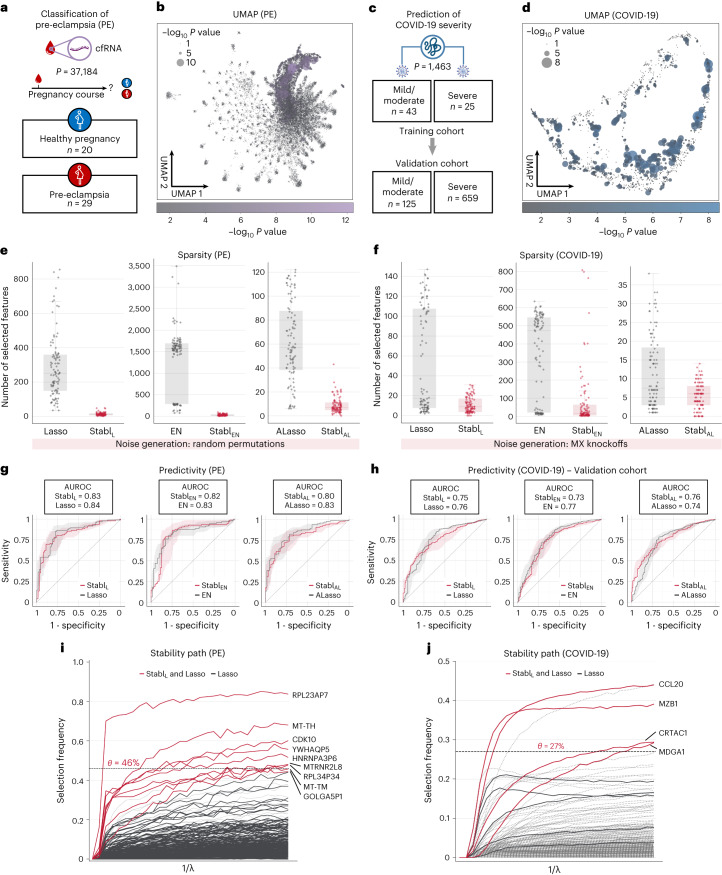
Stabl’s performance on transcriptomic and proteomic data. **a**, Clinical case study 1: classification of individuals with normotensive pregnancy or PE from the analysis of circulating cfRNA sequencing data. The number of samples (*n*) and features (*p*) are indicated. **b**, UMAP visualization of the cfRNA transcriptomic features; node size and color are proportional to the strength of the association with the outcome. **c**, Clinical case study 2: classification of mild versus severe COVID-19 in two independent patient cohorts from the analysis of plasma proteomic data (Olink). **d**, UMAP visualization of the proteomic data. Node characteristics as in **b**. **e**,**f**, Sparsity performances (the number of features selected across *n* = 100 CV iterations, median and IQR) on the PE (**e**) and COVID-19 (**f**) datasets for Stabl_L_ (left), Stabl_EN_ (middle) and Stabl_AL_ (right). **g**,**h**, Predictivity performances (AUROC, median and IQR) on the PE (**g**) and COVID-19 (**h**, validation set; training set shown in Supplementary Table 5) datasets for Stabl_L_ (left), Stabl_EN_ (middle) and Stabl_AL_ (right). Stabl_SRM_ performances are shown using random permutations for the PE dataset and MX knockoffs for the COVID-19 dataset. Median and IQR values comparing Stabl_SGL_ performances to the cognate SRM are listed numerically in Supplementary Table 5. Results in the COVID-19 dataset using random permutations are also shown for Stabl_L_ in Supplementary Table 5. **i**,**j**, Stabl_L_ stability path graphs depicting the relationship between the regularization parameter and the selection frequency for the PE (**i**) and COVID-19 (**j**) datasets. The reliability threshold (*θ*) is indicated (dotted line). Features selected by Stabl_L_ (red lines) or Lasso (black lines) are shown. Significance between outcome groups was calculated using a two-sided Mann–Whitney test. Box plots indicate median and IQR; whiskers indicate 1.5× IQR.

Stabl_SRM_ was first applied to two single-omic clinical datasets. The first study comprised a large-scale plasma cfRNA dataset (*p* = 37,184 features) and aimed to classify pregnancies as either normotensive or pre-eclamptic (PE) (Fig. 4a,b)^26,27^. The second study, involving high-plex plasma proteomics (*p* = 1,463 features, Olink Explore 1536 assay), aimed to classify coronavirus disease 2019 (COVID-19) severity in two independent cohorts (a training cohort and a validation cohort) of patients positive for severe acute respiratory syndrome coronavirus 2 (SARS-CoV-2) (Fig. 4c,d)^28,29^. Although both Lasso and EN models achieved very good predictive performance (AUROC = 0.74–0.84) in these examples, suggesting that they have a robust biological signal with diagnostic potential^30,31^, the lack of model sparsity or reliability hindered the identification of a manageable number of candidate biomarkers, necessitating additional feature selection methods that were decoupled from the predictive modeling process^26–29^.

Consistent with the results obtained using synthetic data, Stabl_L_, Stabl_EN_ and Stabl_AL_ demonstrated improved sparsity compared to Lasso, EN and AL, respectively (Fig. 4e,f and Supplementary Table 5). For the PE dataset, Stabl_SRM_ selected over 20-fold fewer features compared to Lasso or EN and eight-fold fewer compared to AL (Fig. 4e). For COVID-19 classification, Stabl_SRM_ reduced the number of features by factors of 1.9, >20 and 1.25 for Lasso, EN and AL, respectively (Fig. 4f). Remarkably, Stabl_L_, Stabl_EN_ and Stabl_AL_ maintained similar predictive performance to their respective SRMs on both datasets (Fig. 4g,h) despite this favorable feature reduction.

Comparing Stabl_L_ to SS using fixed frequency thresholds (30%, 50% and 80%; Supplementary Table 6) revealed that SS’s predictivity and sparsity performances varied widely based on the chosen threshold, consistent with synthetic modeling findings, whereas Stabl_L_ consistently optimized sparsity while preserving predictive performance. For example, using SS with a 30% versus a 50% threshold resulted in a 42% decrease in predictivity for the COVID-19 dataset (AUROC_30%_ = 0.85 versus AUROC_50%_ = 0.49), with a model selecting no features. Conversely, for the PE dataset, fixing the frequency threshold at 30% versus 50% yielded a 5.3-fold improvement in sparsity with only a 6% decrease in predictivity (AUROC_30%_ = 0.83 versus AUROC_50%_ = 0.78).

Stabl’s ability to identify fewer, more reliable features streamlined biomarker discovery, pinpointing the most informative biological features associated with the clinical outcome. For simplicity, biological interpretation of predictive model features is provided in the context of the Stabl_L_ analyses (Fig. 4i,j and Supplementary Tables 7 and 8). For example, the Stabl_L_ model comprised nine features, including cfRNAs encoding proteins with fundamental cellular function (for example, CDK10 (ref. ^32^)), providing biologically plausible biomarker candidates. Other features included non-coding RNAs and pseudogenes with yet unknown functions (Fig. 4i). For the COVID-19 dataset, Stabl_L_ identified features that echoed key pathobiological mechanisms of the host’s inflammatory response, such as CCL20, a known element of the COVID-19 cytokine storm^33,34^; CRTAC1, a newly identified marker of lung function^35–37^; and MZB1, a protein associated with high neutralization antibody titers after COVID-19 infection (Fig. 4j)^28^. The Stabl_L_ model also selected MDGA1, a previously unknown candidate biomarker of COVID-19 severity.

#### Application of Stabl_SRM_ to multi-omic clinical datasets

We extended the assessment of Stabl to complex clinical datasets combining multiple omic technologies, comparing Stabl_L_, Stabl_EN_ and Stabl_AL_ to late-fusion Lasso, EN and AL, respectively, for predicting a continuous outcome variable from a triple-omic dataset and a binary outcome variable from a double-omic dataset.

**Fig. 5:**
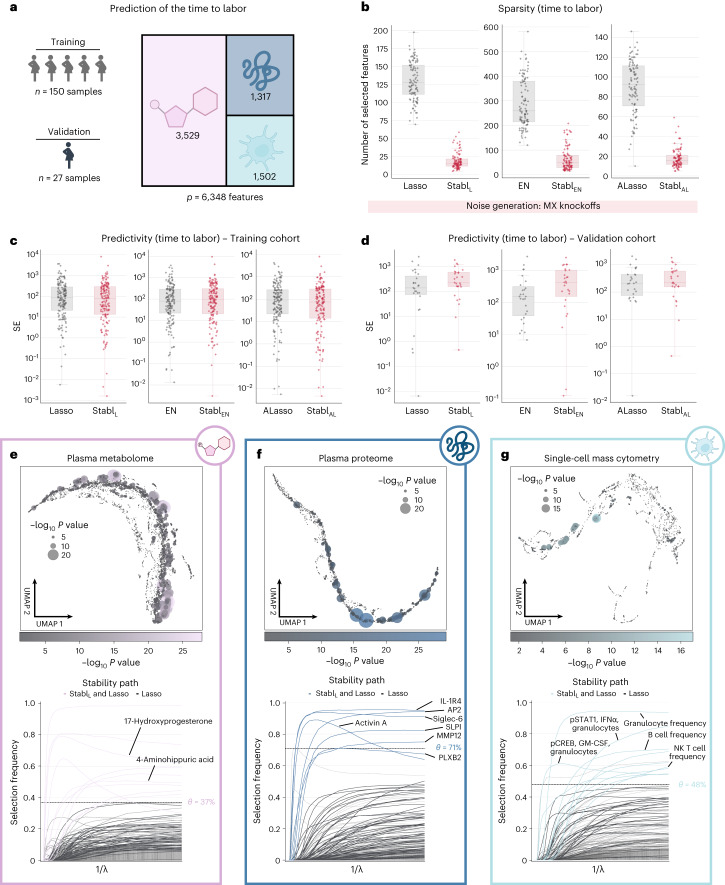
Stabl’s performance on a triple-omic data integration task. **a**, Clinical case study 3: prediction of the time to labor from longitudinal assessment of plasma proteomic (SomaLogic), metabolomic (untargeted mass spectrometry) and single-cell mass cytometry data in two independent cohorts of pregnant individuals. **b**, Sparsity performances (number of features selected across CV iterations, median and IQR) for Stabl_L_ (left), Stabl_EN_ (middle) and Stabl_AL_ (right) compared to their respective SRM (late-fusion data integration method) across *n* = 100 CV iterations. **c**,**d**, Predictivity performances as squared error (SE) on the training (*n* = 150 samples, **c**) and validation (*n* = 27 samples, **d**) datasets for Stabl_L_ (left), Stabl_EN_ (middle) and Stabl_AL_ (right). Stabl_SRM_ performances are shown using MX knockoffs. Results using random permutations are shown for Stabl_L_ in Supplementary Table 5. Median and IQR values comparing Stabl_SRM_ performances to their cognate SRMs are listed in Supplementary Table 5. **e**–**g**, UMAP visualization (upper) and stability path (lower) of the metabolomic (**e**), plasma proteomic (**f**) and single-cell mass cytometry (**g**) datasets. UMAP node size and color are proportional to the strength of association with the outcome. Stability path graphs denote features selected by Stabl_L_. The data-driven reliability threshold *θ* is computed for each individual omic dataset and is indicated by a dotted line. Significance of the association with the outcome was calculated using Pearson’s correlation. Box plots indicate median and IQR; whiskers indicate 1.5× IQR.

The first analysis leveraged a unique longitudinal biological dataset collected in independent training and validation cohorts of pregnant individuals (Fig. 5a)^38^, aiming to predict the time to labor onset, an important clinical need^39,40^. The triple-omic dataset included plasma proteomics (*p* = 1,317 features, SomaLogic), metabolomics (*p* = 3,529 untargeted mass spectrometry features) and single-cell mass cytometry (*p* = 1,502 immune cell features) (Methods). Relative to late-fusion Lasso, EN or AL, the Stabl_L_, Stabl_EN_ and Stabl_AL_ models selected fewer features (Fig. 5b) while estimating the time to labor with similar predictivity (training and validation cohorts; Fig. 5c,d). Stabl_SRM_ calculated a unique reliability threshold for each omic layer (for example, *θ*[Proteomics] = 71%, *θ*[Metabolomics] = 37% and *θ*[mass cytometry] = 48%, for Stabl_L_; Fig. 5e–g). These results emphasize the advantage of data-driven thresholds, as a fixed, common frequency threshold across all omic layers would have been suboptimal, risking over-selecting or under-selecting features in each omic dataset for integration into the final predictive model.

From a biological perspective, Stabl streamlined the interpretation of our previous multivariable analyses^38^, honing in on sentinel elements of a systemic biological signature predicting labor onset, valuable for developing a blood-based diagnostic test. The Stabl model highlighted dynamic changes in 10 metabolomic, seven proteomic and 10 immune cell features with approaching labor (Fig. 5e–g and Supplementary Table 9), including a regulated decrease in innate immune cell frequencies (for example, neutrophils) and their responsiveness to inflammatory stimulation (for example, the pSTAT1 signaling response to IFNα in natural killer (NK) cells^41,42^), along with a synchronized increase in pregnancy-associated hormones (for example, 17-hydroxyprogesterone^43^), placental-derived proteins (for example, Siglec-6 (ref. ^44^) and angiopoietin 2/sTie2 (ref. ^45^)) and immune regulatory plasma proteins (for example, IL-1R4 (ref. ^46^) and SLPI47 (ref. ^47^)).

The use cases provided thus far featured models with good to excellent predictive performance. Stabl was also tested on a dataset where previous models did not perform as well (AUROC < 0.7). The Microbiome Preterm Birth DREAM challenge aimed to classify pre-term (PT) and term (T) labor pregnancies using nine publicly available vaginal microbiome (phylotypic and taxonomic) datasets^48,49^. The top 20 models submitted by 318 participating analysis teams achieved AUROC scores between 0.59 and 0.69 for the task of predicting PT delivery. When applied to a subset of this dataset (*n* = 1,569 samples, 609 T and 960 PT deliveries), Stabl_L_ and Stabl_EN_ achieved better sparsity at similar predictive performance compared to late-fusion Lasso and EN (Supplementary Table 5).

#### Identifying promising candidate biomarkers from a new multi-omic dataset

**Fig. 6:**
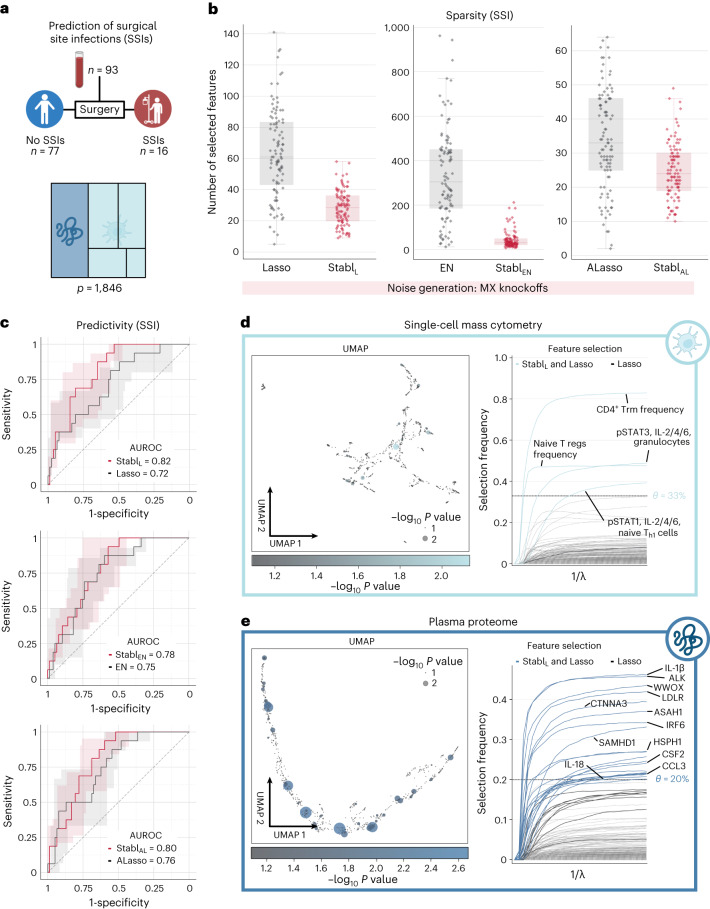
Candidate biomarker identification using Stabl for analysis of a newly generated multi-omic clinical dataset. **a**, Clinical case study 5: prediction of post-operative SSIs from combined plasma proteomic and single-cell mass cytometry assessment of pre-operative blood samples in patients undergoing abdominal surgery. **b**, Sparsity performances (the number of features selected across *n* = 100 CV iterations) for Stabl_L_ (left), Stabl_EN_ (middle) and Stabl_AL_ (right) compared to their respective SRMs (late-fusion data integration method). **c**, Predictivity performances (AUROC) for Stabl_L_ (upper), Stabl_EN_ (middle) and Stabl_AL_ (lower). Stabl_SRM_ performances are shown using MX knockoffs. Results using random permutations are shown in Supplementary Table 5. Median and IQR values comparing Stabl_SRM_ performances to their cognate SRMs are listed in Supplementary Table 5. **d**,**e**. UMAP visualization (left) and stability path (right) of the mass cytometry (**d**) and plasma proteomic (**e**) datasets. UMAP node size and color are proportional to the strength of association with the outcome. Stability path graphs denote features selected by Stabl_L_. The data-driven reliability threshold *θ* is computed for individual omic datasets and indicated by a dotted line. Significance of the association with the outcome was calculated using a two-sided Mann–Whitney test. Box plots indicate median and IQR; whiskers indicate 1.5× IQR.

**Extended Data Fig. 10:**
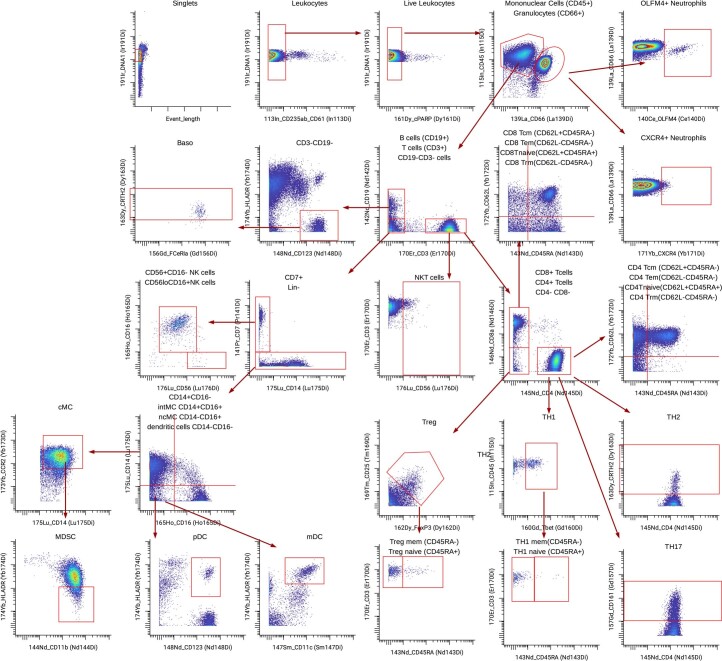
Gating strategy for mass cytometry analyses (SSI dataset). Live, non-erythroid cell populations were used for analysis.

Application of Stabl to the four existing omic datasets demonstrated the algorithm’s performance in biomarker discovery studies with known biological signal. To complete its systematic evaluation, Stabl was applied to our multi-omic clinical study performing an unbiased biomarker discovery task. The aim was to develop a predictive model for identifying patients at risk for post-operative surgical site infection (SSI) from analysis of pre-operative blood samples collected from 274 enrolled patients (Fig. 6a). Using a matched, nested case–control design, 93 patients were selected from the larger cohort to minimize the influence of clinical or demographic confounders on identified predictive models (Supplementary Table 10). These samples were analyzed using a combined single-cell mass cytometry (Extended Data Fig. 10 and Supplementary Table 11) and plasma proteomics (SomaLogic) approach.

Stabl merged all omic datasets into a final model that accurately classified patients with and without SSI (Stabl_L_: AUROC = 0.82 (0.71, 0.90); Stabl_EN_: AUROC = 0.78 (0.68, 0.88); and Stabl_AL_: AUROC = 0.80 (0.70, 0.89)). Compared to late-fusion Lasso, EN and AL, Stabl_L_, Stabl_EN_ and Stabl_AL_ had superior sparsity performances (Fig. 6b) yet similar predictive performances (Fig. 6c). The frequency-matching procedure ensured that major demographic and clinical variables did not differ significantly between patient groups, suggesting that model predictions were primarily driven by pre-operative biological differences in patients’ SSI susceptibility.

Stabl_L_ selected four mass cytometry and 21 plasma proteomic features, combined into a biologically interpretable immune signature predictive of SSI. Examination of Stabl_L_ features unveiled cell-type-specific immune signaling responses associated with SSI (Fig. 6d), which resonated with circulating inflammatory mediators (Fig. 6e and Supplementary Table 12). Notably, the model revealed elevated STAT3 signaling response to IL-6 in neutrophils before surgery in patients predisposed to SSI. Correspondingly, patients with SSI had increased plasma levels of IL-1β and IL-18, potent inducers of IL-6 production in response to inflammatory stress^50,51^. Other selected proteomic features included CCL3, which coordinates recruitment and activation of neutrophils, and the canonical stress response protein HSPH1. These findings concur with previous studies indicating that heightened innate immune cell responses to inflammatory stress, such as surgical trauma^52,53^, can result in diminished defensive responses to bacterial pathogens^39^, increasing susceptibility to subsequent infection.

Altogether, application of Stabl in a biomarker discovery study provided a manageable number of candidate SSI biomarkers, pointing at plausible biological mechanisms that can be targeted for further diagnostic or therapeutic development.

## Discussion

Stabl is a machine learning framework developed to facilitate clinical translation of high-dimensional omic biomarker studies. Through artificial noise injection and minimization of a proxy for FDP, Stabl enables data-driven selection of sparse and reliable biomarker candidates within a multivariable predictive modeling architecture. The modular framework of Stabl allows for customization across various SRMs and noise injection techniques, catering to the specific requirements of individual studies. When applied to real-world biomarker discovery tasks spanning different omic technologies, single-omic and multi-omic datasets and clinical endpoints, Stabl consistently demonstrates its adaptability and effectiveness in reliable selection of biologically interpretable biomarker candidates conducive to further clinical translation.

Stabl builds upon earlier methodologies, including SS and MX knockoff. These approaches aim to improve reliability of sparse learning algorithms by incorporating bootstrapping or artificial features^7,18,20,22^. However, they typically rely on fixed or user-defined frequency thresholds to distinguish informative from uninformative features. In practical scenarios where *p* ≫ *n*, determining the optimal frequency threshold without prior data knowledge is challenging, as illustrated by our synthetic modeling results. This reliance on prior knowledge limits these methods to feature selection only.

Stabl improves on these methodologies by experimentally and, under certain assumptions, theoretically extending FDR control techniques devised for MX knockoff and random permutation noise^19,54,55^. Minimizing the FDP_+_ offers two key advantages: it balances the tradeoff between reliability and sparsity by combining an increasing and decreasing function of the threshold, and, assuming exchangeability between artificial and uninformative features, it guarantees a stochastic upper bound on FDP using the reliability threshold, ensuring reliability during the optimization procedure. By minimizing this function ex-ante, Stabl objectively defines a model fit without requiring prior data knowledge.

Experimental results on synthetic datasets demonstrate Stabl’s ability to select an optimal reliability threshold by minimizing FDP_+_, leading to improved reliability and sparsity compared to popular SRMs such as Lasso, EN, SGL or AL, all while maintaining similar predictivity performance. These findings hold across different data distributions, correlation structures and prediction tasks. When applied to real-world omic studies, Stabl consistently performs favorably compared to other SRMs. In each case study, identification of a manageable number of reliable biomarkers facilitated the interpretation of the multivariable predictive models. Stabl embeds the discovery of reliable candidate biomarkers within the predictive modeling process, eliminating the need for separate analyses that risk overfitting, such as post hoc analyses with user-defined cutoffs after the initial model fitting or the selection of clinical endpoint-associated features before modeling.

Stabl’s versatility extends to multi-omic datasets, offering an alternative that avoids the potential shortcomings of early-fusion and late-fusion strategies. Although early fusion combines all omic data layers for joint optimization, regardless of each dataset’s unique properties, and late fusion independently fits models for each omic before integrating predictions without weighing features from different omics against each other^11,12^, Stabl computes a distinct reliability threshold for each omic layer, tailoring its approach to the specific dataset. This enables integration of selected features into a final modeling layer, a capability that was particularly useful for analysis of our dataset involving patients undergoing surgery. Stabl identified a patient-specific immune signature spanning both plasma and single-cell datasets that appears to be programmed before surgery and predictive of SSIs.

Our study has limitations. The assumption of exchangeability between artificial and uninformative features underpins our theoretical guarantee, which builds on a recent line of research focused on constructing artificial features to establish control over the FDR^19,21,23,54–56^. Hence, Stabl’s validity hinges on the accuracy of the artificial feature generation technique. Future efforts will investigate relaxing the exchangeability assumption by exploring pairwise exchangeability settings to accommodate a wider range of data scenarios where complete exchangeability may not hold^19^. Additionally, improving knockoff generation methods, such as deep knockoff^57^ and metropolized knockoff^25^, may enhance the robustness and flexibility of our approach in handling diverse data distributions and structures. We also observed that Stabl can be overly conservative. However, Stabl is designed to optimize reliability, sparsity and predictivity performances simultaneously, which can result in feature under-selection when only a subset of informative features is sufficient for optimal predictive performance. Other algorithms addressing these performance tasks individually, such as double machine learning^58^ for reliability, Boruta^59^ for sparsity and random forest^60^ or gradient boosting^61^ for predictivity, warrant further evaluation to systematically investigate each method’s performance in comparison to, or integrated with, the Stabl statistical framework. Finally, integrating emerging algorithms for multi-omic data, such as cooperative multiview learning^11^, may further enhance Stabl’s capabilities in multi-omic modeling tasks.

Analysis of high-dimensional omic data has transformed biomarker discovery, necessitating adjustments to machine learning methods to facilitate clinical translation. Stabl addresses key requirements of an effective biomarker discovery pipeline by offering a unified supervised learning framework that bridges the gap between predictive modeling of clinical endpoints and selection of reliable candidate biomarkers. Across diverse real-world single-omic and multi-omic datasets, Stabl identified biologically meaningful biomarker candidates, providing a robust machine learning pipeline that holds promise for generalization across all omic data.

## Methods

### Notations

Given a vector of outcomes $Y\in {{\mathbb{R}}}^{n}$ and a matrix of covariates ${{{\boldsymbol{X}}}}\in {{\mathbb{R}}}^{n\times p}$, where *n* denotes the number of observations (sample size) and *p* denotes the number of covariates (features) in each sample. We are interested in estimating parameters $\beta ={({\beta }_{1},\ldots ,{\beta }_{p})}^{{\mathsf{T}}}\in {{\mathbb{R}}}^{p}$, within the linear model:

$$Y={{{\boldsymbol{X}}}}\beta +\varepsilon \,.$$

Here, *ε* is an unknown noise vector, which is centered and independent of ***X***.

We denote the columns of ***X*** by ${X}_{1},\ldots ,{X}_{p}\in {{\mathbb{R}}}^{n}$ and the entries of *Y* by *y*_1_, …, *y*_*n*_. We denote by *S* ≔ {*i* ∈ [*p*]: *β*_*i*_ ≠ 0} the set of informative features and by *N* ≔ {*i* ∈ [*p*]: *β*_*i*_ = 0} the set of uninformative features. Throughout [*m*] ≔ {1, …, *m*} is the set of first *m* integers, and ∣*A*∣ is the cardinality of a set *A*.

Our main objective is to estimate *S*, and we will generally denote by $\hat{S}$ an estimator of this set. Given coefficient estimates ${\hat{\beta }}=({{\hat{\beta }}_{1},\ldots ,{\hat{\beta }}_{p}})^{{\mathsf{T}}}$, an estimate of *S* can be constructed using the support of $\hat{\beta }$. We will denote this by $\hat{S}(\hat{\beta }):= \{i\in [p]:\,{\hat{\beta }}_{i}\ne 0\}$.

### Lasso, EN, AL and SGL

Motivated by omic application, our main focus is on the high-dimensional regime *p* ≫ *n*. Lasso is a regression method that uses an *ℓ*_1_-regularization penalty to yield sparse solutions^7^. Denoting with *λ* the regularization parameter, Lasso estimate is defined by:

$${\hat{\beta }}_{{{\mbox{Lasso}}}}(\lambda )=\arg \mathop{\min }\limits_{b\in {{\mathbb{R}}}^{p}}\left\{\parallel Y-{{{\boldsymbol{X}}}}b{\parallel }_{2}^{2}+\lambda \parallel b{\parallel }_{1}\right\}\,.$$

For sparse linear models, and under suitable conditions on the design matrix ***X*** (for example, restricted isometry or restricted eigenvalue conditions), the Lasso is known to provide consistent estimates of *β*, for certain choices of *λ* (refs. ^62–64^). It is also known that the Lasso can yield consistent variable selection—that is, $| \hat{S}({\hat{\beta }}_{{{\mbox{Lasso}}}}(\lambda ))\cap S| +| \hat{S}({\hat{\beta }}_{{{\mbox{Lasso}}}}(\lambda ))\cap N| \to 0$ (refs. ^65,66^). However, variable selection consistency requires stronger conditions on ***X***, such as the irrepresentability or the generalized irrepresentability condition^65,67^.

EN is a regression method that combines *ℓ*_1_-regularization and *ℓ*_2_-regularization penalties^8^. Denoting by *λ*_1_ and *λ*_2_ the regularization parameters of these two penalties, the EN estimate is defined by:

$${\hat{\beta }}_{{{\mbox{EN}}}}({\lambda }_{1},{\lambda }_{2})=\arg \mathop{\min }\limits_{b\in {{\mathbb{R}}}^{p}}\left\{\parallel Y-{{{\boldsymbol{X}}}}b{\parallel }_{2}^{2}+{\lambda }_{1}\parallel b{\parallel }_{1}+{\lambda }_{2}\parallel b{\parallel }_{2}\right\}\,.$$

Although we will mostly focus on Lasso as our basic estimator, this can be replaced by EN or other sparse regression methods without much change to our overall methodology.

AL is a regression method based on the Lasso with adaptive weights to penalize different coefficients in the *ℓ*_1_ penalty differently. To define the model, first we need $\hat{\beta }$, a root-n-consistent estimator to *β*, and we can consider *β*_OLS_. Then, choose a *γ* > 0 and define $\hat{w}=| \hat{\beta }{| }^{-\gamma }$. As such, denoting with *λ* the regularization parameter, the AL estimate is defined by:

$${\hat{\beta }}_{AL}(\lambda )=\arg \mathop{\min }\limits_{b\in {{\mathbb{R}}}^{p}}\left\{\parallel Y-{{{\boldsymbol{X}}}}b{\parallel }_{2}^{2}+\lambda \mathop{\sum }\limits_{j=1}^{p}{\hat{w}}_{j}| {b}_{j}| \right\}\,.$$

The weighted Lasso above can be solved with the same algorithm used to solve the Lasso. With well-chosen weights and regularization parameters, AL also enjoys the oracle property^9^:

${\mathbb{P}}(\hat{S}({\hat{\beta }}_{AL}(\lambda ))=S)\to 1$ and $\sqrt{n}({\hat{\beta }}_{AL}(\lambda )-\beta ){\to }_{d}{{{\mathcal{N}}}}(0,{{{\Sigma }}}^{* })$, with Σ^*^ the covariance matrix of the true subset model.

SGL extends the concept of sparse regression methods for problems with group covariates, with sparsity on both within and between groups in high-dimensional settings^10^. The SGL penalty can be formulated as:

$${\hat{\beta }}_{{{{\rm{SGL}}}}}({\lambda }_{1},{\lambda }_{2})=\arg \mathop{\min }\limits_{b\in {{\mathbb{R}}}^{p}}\left\{\frac{1}{2n}\parallel Y-Xb{\parallel }_{2}^{2}+{\lambda }_{1}\parallel b{\parallel }_{1}+{\lambda }_{2}\mathop{\sum }\limits_{g=1}^{G}\sqrt{{p}_{g}}\parallel {b}_{{{{{\mathcal{G}}}}}_{g}}{\parallel }_{2}\right\}$$

where *G* is the number of groups, and *p*_*g*_ denotes the number of covariates in group *g*. The first term in the objective function measures the data-fitting loss, whereas the second and third terms enforce sparsity at the individual feature level and group level, respectively.

### SS

SS^18^ is a technique to improve variable selection in high-dimensional methods, including the Lasso. The algorithm uses Lasso on subsamples of the original data $(Y,{{{\boldsymbol{X}}}}\,)\in {{\mathbb{R}}}^{n\times (p+1)}$. At each iteration *k* ∈ {1, …, *B*}, a different subsample (*Y*, ***X***)^*k*^ of size ⌊*n*/2⌋ × (*p* + 1) is selected. Lasso is used to fit a linear model on (*Y*, ***X***)^*k*^ over a range of regularization parameters $\lambda \in {{\Lambda }}\subseteq {{\mathbb{R}}}_{\ge 0}$. This yields an estimate that we denote by:

$$\hat{\beta }(k,\lambda )={({\hat{\beta }}_{1}(k,\lambda ),\ldots ,{\hat{\beta }}_{p}(k,\lambda ))}^{{\mathsf{T}}}\,.$$

After *B* iterations, it is possible, for any feature *i* and regularization parameter *λ*, to define a ‘frequency of selection’ *f*_*i*_ measuring how often feature *i* was selected by Lasso:

$${f}_{i}(\lambda )=\frac{1}{B}\mathop{\sum }\limits_{k=1}^{B}{{{{\bf{1}}}}}_{\left[{\hat{\beta }}_{i}(k,\lambda )\ne 0\right]}$$

Plotting *f*_*i*_ as a function of 1/*λ* yields a ‘stability path’ for feature *i*. Plotting all stability paths on the same graph yields a ‘stability graph’. Denoting the ‘selection threshold’ by *t* ∈ (0, 1), selected features are those whose stability path *f*_*i*_(*λ*) crosses the line *y* = *t*. In other words, the set of stable features is defined as:

$${\hat{S}}_{{{\mbox{SS}}}}(t)=\left\{i\in [p]:\ \mathop{\max }\limits_{\lambda \in {{\Lambda }}}{f}_{i}(\lambda )\ge t\right\}$$

Notice that, in SS, *t* is arbitrary in that it has to be defined ex-ante. The threshold value is a tuning parameter whose influence is very small^18^. However, we observe that, in some cases, the results are sensitive to the chosen threshold, thereby motivating the development of a data-driven threshold optimization.

### Stabl framework

#### Preliminaries

Our algorithm builds upon the framework of SS and provides a way to define a data-driven threshold by optimizing a surrogate for the FDP. We construct such a surrogate by introducing artificially generated features in the Lasso regression. We thus build upon a recent fruitful line of work that develops several constructions of such artificial features and establishes control of the FDR under varying assumptions^23,54–56^.

The general Stabl procedure can accommodate a variety of feature-generating procedures. In our implementation, we experimented with two specific constructions:

- Random permutation of the original features^55^
- MX knockoffs^19^

#### Stabl algorithm

The initial step of the Stabl procedure involves selecting a base SRM (for example, Lasso, AL, EN and SGL), in which case the procedure is denoted Stabl_SRM_. It runs as follows:

- From the original matrix ${{{\boldsymbol{X}}}}=({X}_{1},\ldots ,{X}_{p})\in {{\mathbb{R}}}^{n\times p}$, we generate a matrix $\tilde{{{{\boldsymbol{X}}}}}=(\tilde{{X}_{1}},\ldots ,\tilde{{X}_{p}})\in {{\mathbb{R}}}^{n\times p}$ of artificial features of the same dimensions as the original matrix.
- We concatenate the original matrix ***X*** and the artificial matrix $\tilde{{{{\boldsymbol{X}}}}}$, and define:
  $${\mathbb{X}}=\left[\,{{{\boldsymbol{X}}}}\,\left\vert \right.\,\tilde{{{{\boldsymbol{X}}}}}\,\right]\in {{\mathbb{R}}}^{n\times 2p}$$
  All the following steps run using the ${\mathbb{X}}$ matrix as input. We denote by $A=\left\{p+1,\ldots ,2p\right\}$ the set of artificial features and by $O=\left\{1,\ldots ,p\right\}$ the set of original features. In the context of SGL, an extra layer of information regarding feature groupings is needed. Specifically, each feature requires supplementary information about its respective group assignment. To adapt the procedure to this requirement, each artificial feature is linked to the group of its original feature source.
- We fix *B* the number of subsampling iterations. At each iteration $k\in \left\{1,\ldots ,B\right\}$, a subsample of size ⌊*n*/2⌋ is drawn without replacement from $(Y,{\mathbb{X}})$, denoted by ${(Y,{\mathbb{X}})}^{k}\in {{\mathbb{R}}}^{\lfloor n/2\rfloor ,2p+1}$. The size of subsamples could be ⌊*αn*⌋ with *α* ∈ (0, 1). Selecting subsamples of size ⌊*n*/2⌋ most closely resembles the bootstrap while allowing computationally efficient implementation^18^.
- We use the base SRM to fit a model on data ${(Y,{\mathbb{X}})}^{k}$ for different values of regularization parameters *λ* ∈ Λ. For models with only one penalization (Lasso and AL), ${{\Lambda }}\subset {{\mathbb{R}}}_{+}^{* }$. For models with two penalizations (EN and SGL), ${{\Lambda }}\subset {{{\mathbb{R}}}_{+}^{* }}^{2}$. For each set of hyperparameters *λ* (in the context of EN), beyond the conventional pursuit of the *ℓ*_1_-regularization parameter, we introduce three distinct options for determining the parameter that governs the equilibrium between *ℓ*_1_ and *ℓ*_2_ regularization. This results in the creation of a hyperparameter set, within which the maximum value is selected for each feature; this yields an estimate $\hat{\beta }(k,\lambda )$ defined as:
  $$\hat{\beta }(k,\lambda )={({\hat{\beta }}_{1}(k,\lambda ),\ldots ,{\hat{\beta }}_{2p}(k,\lambda ))}^{{\mathsf{T}}}$$
- For each feature *j*, the maximum frequency of selection over Λ is computed. In the case of models with two hyperparameters (EN and SGL), this leads to a two-dimensional optimization.
  $$\begin{array}{r}{f}_{j}=\mathop{\max }\limits_{\lambda \in {{\Lambda }}}\,{f}_{j}(\lambda )=\mathop{\max }\limits_{\lambda \in {{\Lambda }}}\,\frac{1}{B}\mathop{\sum }\limits_{k=1}^{B}{{{{\bf{1}}}}}_{\left[{\hat{\beta }}_{j}(k,\lambda )\ne 0\right]}\end{array}$$
- For a given frequency threshold *t* ∈ [0, 1], a feature *j* is selected if *f*_*j*_ ≥ *t*. We define the augmented FDP at *t* by
  $${{{{\rm{FDP}}}}}_{+}(t)=\frac{1+{\sum }_{j\in A}{{{{\bf{1}}}}}_{[{f}_{j}\ge t]}}{{\sum }_{j\in O}{{{{\bf{1}}}}}_{[{f}_{j}\ge t]}\vee 1}$$
  The set of selected features at *t* is $\hat{S}(t):= \{\,j\in O:\ {f}_{j}\ge t\}$.
- We define the reliability threshold as:
  1 $$\theta \in \arg \mathop{\min }\limits_{t\in [0,1]}{{{{\rm{FDP}}}}}_{+}(t)=\arg \mathop{\min }\limits_{t\in [0,1]}\frac{1+{\sum }_{j\in A}{{{{\bf{1}}}}}_{[{f}_{j}\ge t]}}{{\sum }_{j\in O}{{{{\bf{1}}}}}_{[{f}_{j}\ge t]}\vee 1}\,,$$
  which results in a selected feature set $\hat{S}(\theta )$. When multiple minimizers exist, we select one arbitrarily (but, in practice, we always found a unique minimizer). At *θ*, we achieve the following augmented FDP:
  2 $${q}_{+}={{{{\rm{FDP}}}}}_{+}(\theta )=\mathop{\min }\limits_{t\in [0,1]}{{{{\rm{FDP}}}}}_{+}(t)\,.$$
- We obtain the final estimate for the Stabl model using:
  $$\begin{array}{r}{\hat{\beta }}_{{{\mbox{Stabl}}}}=\arg \mathop{\min }\limits_{b\in {{\mathbb{R}}}^{p}}\parallel Y-{{{\boldsymbol{X}}}}b{\parallel }_{2}^{2}\\ {{{\rm{s.t.}}}}\,{b}_{i}=0{{{\rm{if}}}}i\notin \hat{S}(\theta )\end{array}$$

#### Link with FDP and FDR

FDP and FDR^68^ are classical metrics to assess the quality of a model selection method. Consider a general method parameterized by a threshold *t* ∈ (0, 1) (for example, the stability threshold in our approach). For any fixed *t*, the method returns a selected subset of features $\hat{S}(t)$, resulting in the FDP

$${{{\rm{FDP}}}}(t):= \frac{| N\cap \hat{S}(t)| }{| \hat{S}(t)| \vee 1}\,.$$

Several approaches use a threshold $\hat{t}=\hat{t}(Y,{{{\boldsymbol{X}}}})$ that is dependent on the data. The resulting FDP ${{{\rm{FDP}}}}(\hat{t})$ is a random quantity at fixed $\hat{t}=t$ and is also random because it is evaluated at a random threshold. An important goal of a model selection procedure is to achieve a small ${{{\rm{FDP}}}}(\hat{t})$ with as large a probability as possible. Often the distribution of ${{{\rm{FDP}}}}(\hat{t})$ is summarized via the FDR

$${{{{\rm{FDR}}}}}_{\hat{t}}:= {\mathbb{E}}\left\{{{{\rm{FDP}}}}(\hat{t})\right\}\,.$$

Because $| N\cap \hat{S}|$ is not observed, several methods estimate it by constructing a set of artificial features that share common behavior with the uninformative features^19,21,23,54,56^. In all of these cases, the artificial features are used to construct a surrogate of FDP(*t*) that we denoted in the previous section by FDP_+_(*t*).

The key distinction between Stabl and previous work is in the selection of the threshold $\hat{t}$. Previous approaches start by fixing a target FDR, denoted by *q* ∈ (0, 1), and then set

3 $$\hat{t}:= \min \left\{t\in (0,1):\ {{{{\rm{FDP}}}}}_{+}(t)\le q\right\}\,.$$

In contrast, we choose the Stabl threshold *θ* by minimizing FDP_+_(*t*) over *t* ∈ (0, 1) as per equation (1). The resulting observed FDP surrogate *q*_+_, defined in equation (2), is now a random variable.

Although the idea of minimizing FDP_+_(*t*) over *t* is very natural from an empirical viewpoint, it is less natural mathematically. Indeed, earlier work exploits in a crucial way the fact that $\hat{t}$ defined via equation (3) is a stopping time for a suitably defined filtration, to conclude that

4 $${{{{\rm{FDR}}}}}_{\hat{t}}:= {\mathbb{E}}\left\{{{{{\rm{FDP}}}}}_{+}(\hat{t})\right\}\le q\,.$$

In contrast, our threshold *θ* is not a stopping time, and, therefore, a similarly simple argument is not available. Related to this is the fact that *q*_+_ is itself random.

We carried out numerical simulations on synthetic data (compare to Section 4.6). We observe empirically that often

5 $${{{{\rm{FDR}}}}}_{\theta }:= {\mathbb{E}}\{{{{\rm{FDP}}}}(\theta )\}\lesssim {\mathbb{E}}\{{q}_{+}\}={\mathbb{E}}\{{{{{\rm{FDP}}}}}_{+}(\theta )\}\,.$$

In the next section, we will provide mathematical support for this finding.

#### Theoretical guarantees

We will establish two bounds on the FDP achieved by Stabl, under the following exchangeability assumption.

##### Assumption 1

Exchangeability of the extended null set. Denote by ${{{{\boldsymbol{X}}}}}_{S}:= {({X}_{i})}_{i\in S}$ the covariates in the informative set and by ${{{{\boldsymbol{X}}}}}_{N\cup A}:= {({X}_{i})}_{i\in N\cup A}$ the covariates in the null set or in the artificial set. We assume that ***X***_*N*∪*A*_ is exchangeable. Namely, for any permutation *π* of the set *N* ∪ *A*, we have

6 $$(Y,{{{{\boldsymbol{X}}}}}_{S},{{{{\boldsymbol{X}}}}}_{N\cup A}^{\pi })\mathop{=}\limits^{{{{\rm{d}}}}}(Y,{{{{\boldsymbol{X}}}}}_{S},{{{{\boldsymbol{X}}}}}_{N\cup A}).$$

(Here, ${{{{\boldsymbol{X}}}}}_{N\cup A}^{\pi }$ is the matrix obtained by permuting the columns of ***X***_*N*∪*A*_ using *π*, and $\mathop{=}\limits^{{{{\rm{d}}}}}$ denotes equality in distribution.)

Our first result establishes that the true FDP(*θ*) cannot be much larger than the minimum value of the FDP surrogate, ${q}_{+}=\mathop{\min }\nolimits_{t\in (0,1)}{{{{\rm{FDP}}}}}_{+}(t)$, with large probability. We defer proofs to Section 11.4.5.

##### Proposition 1

Under Assumption 1, we have, for any Δ > 0,

7 $$\begin{array}{r}{\mathbb{P}}\left({{{\rm{FDP}}}}(\theta )\ge (1+{{\Delta }}){q}_{+}\right)\le \frac{1}{1+{{\Delta }}}\,.\end{array}$$

Although reassuring, Lemma 1 exhibits only a slow decrease of the probability that FDP(*θ*) ≥ (1 + Δ)*q*_+_ with Δ. A sharper result can be obtained when the optimal threshold is not too high.

##### Theorem 1

Under Assumption 1, further assume ∣*S*∣ ≤ *p*/2. Let $M:= | N\cap \hat{S}(\theta )| +| A\cap \hat{S}(\theta )|$ be the total number of false discoveries (including those among artificial features). Then, there exist constants c_*_, C_*_ > 0 such that, for any ${{\Delta }}\in (0,m/{C}_{* }\log p)$,

8 $${\mathbb{P}}\left({{{\rm{FDP}}}}(\theta )\ge (1+{{\Delta }}){q}_{+}\ \,{{\mbox{and}}}\,\ M\ge m\right)\le 2{e}^{-{c}_{* }m{{{\Delta }}}^{2}}\,.$$

This result gives a tighter control of the excess of FDP(*θ*) over the surrogate *q*_+_. It implies that, in the event that the number of false discoveries is at least *m*, we have

9 $${{{\rm{FDP}}}}(\theta )\le \left(1+{O}_{P}\left({m}^{-1/2}\right)\right)\cdot {q}_{+}\,.$$

As should be clear from the proof, the assumption ∣*S*∣ ≤ *p*/2 could be replaced by ∣*S*∣ ≤ (1 − *c*)*p* for any strictly positive constant *c*.

#### Proofs

Throughout this appendix, $c,C,C{\prime} ,\ldots \,$ will be used to denote absolute constants whose value might change from line to line. We begin by defining the stopping time:

10 $${t}_{k}:= \inf \left\{t:\ | \hat{S}(t)\cap N| +| \hat{S}(t)\cap A| \le | N| +| A| -k\right\}\,.$$

In words, *t*_*k*_ is the threshold for the *k*-th-to-last false discovery. We will assume the *t*_*k*_ to be distinct: 0 = *t*_0_ < *t*_1_ < ⋯ < *t*_∣*A*∣+∣*N*∣_ < 1. Indeed, we can always reduce the problem to this case by a perturbation argument. We define ${k}_{\max }:= | N| +| A|$. We let ${n}_{k}:= | \hat{S}({t}_{k})\cap N|$, ${a}_{k}:= | \hat{S}({t}_{k})\cap A|$, and define $\underline{k}(t):= \max (k:\ {t}_{k}\le t)$.

##### Lemma 1

*Under Assumption 1, for any* ${{\Delta }}\in {\mathbb{R}}$, *we have*

11 $${\mathbb{P}}\left({{{\rm{FDP}}}}(\theta )\ge (1+{{\Delta }}){q}_{+}\right)={\mathbb{P}}\left(\frac{{n}_{\underline{k}(\theta )}}{{a}_{\underline{k}(\theta )}+1}\ge (1+{{\Delta }})\right)\,.$$

##### Proof

By definition (recalling that *O* = *S* ∪ *N* is the set of original features):

$$\begin{array}{l}{{{\rm{FDP}}}}(\theta )\,\,=\frac{| \hat{S}(\theta )\cap N| }{| \hat{S}(\theta )\cap O| \vee 1}\\\qquad\qquad =\frac{| \hat{S}(\theta )\cap A| +1}{| \hat{S}(\theta )\cap O| \vee 1}\cdot \frac{| \hat{S}(\theta )\cap N| }{| \hat{S}(\theta )\cap A| +1}\\\qquad\qquad ={{{{\rm{FDP}}}}}_{+}(\theta )\cdot \frac{{n}_{\underline{k}(\theta )}}{{a}_{\underline{k}(\theta )}+1}\\\qquad\qquad={q}_{+}\cdot \frac{{n}_{\underline{k}(\theta )}}{{a}_{\underline{k}(\theta )}+1}\,.\end{array}$$

The claim follows.

We next define ${k}_{0}:= \min (k:\ {a}_{k}=0)$ and

12 $${\underline{Z}}_{k}:= \frac{{n}_{k}}{{a}_{k}+1}\,,\ \ \ \ \ {Z}_{k}:= {\underline{Z}}_{k\vee {k}_{0}}\,.$$

The next result is standard, but we provide a proof for the reader’s convenience.

##### Lemma 2

*Under Assumption 1, the process* ${\big({\underline{Z}}_{k}\big)}_{k\le {k}_{\max }}$ *is a supermartingale with respect to the filtration* ${{{{\mathcal{F}}}}}_{k}:= \sigma (\{{n}_{i},{a}_{i}:i\le k\}\cup \{{f}_{j}:j\in S\})$, *and* ${({Z}_{k})}_{k\le {k}_{\max }}$ *is a martingale. Finally,* ${\underline{Z}}_{k}\le {Z}_{k}$ *for all k*.

##### Proof

By exchangeability, the (*k* + 1)-th false discovery is equally likely to be among any of the *n*_*k*_ + *a*_*k*_ nulls that have not yet been rejected. Hence, conditional joint distribution of *n*_*k*+1_, *a*_*k*+1_ is (for $k < {k}_{\max }$):

$$\begin{array}{rc}{\mathbb{P}}\left({n}_{k+1}={n}_{k}-1,{a}_{k+1}={a}_{k}\left\vert \right.{{{{\mathcal{F}}}}}_{k}\right)&=\frac{{n}_{k}}{{n}_{k}+{a}_{k}}\,,\\ {\mathbb{P}}\left({n}_{k+1}={n}_{k},{a}_{k+1}={a}_{k}-1\left\vert \right.{{{{\mathcal{F}}}}}_{k}\right)&=\frac{{a}_{k}}{{n}_{k}+{a}_{k}}\,.\end{array}$$

Hence, in the event {*k* < *k*_0_} (in which case *a*_*k*_ > 0)

$${\mathbb{E}}\big[{\underline{Z}}_{k+1}| {{{{\mathcal{F}}}}}_{k}\big]=\frac{{n}_{k}}{{n}_{k}+{a}_{k}}\cdot \frac{{n}_{k}-1}{{a}_{k}+1}+\frac{{a}_{k}}{{n}_{k}+{a}_{k}}\cdot \frac{{n}_{k}}{{a}_{k}}={\underline{Z}}_{k}\,.$$

On the other hand, in the event {*k* ≥ *k*_0_}:

$${\mathbb{E}}\left[{\underline{Z}}_{k+1}| {{{{\mathcal{F}}}}}_{k}\right]={\mathbb{E}}[{n}_{k+1}| {{{{\mathcal{F}}}}}_{k}]={n}_{k}-1 < {\underline{Z}}_{k}\,.$$

Hence, ${\underline{Z}}_{k}$ is a supermartingale. The same calculation implies that *Z*_*k*_ is a martingale. The inequality ${\underline{Z}}_{k}\le {Z}_{k}$ follows from the fact that ${\underline{Z}}_{k}$ is decreasing in *k* for *k* ≥ *k*_0_.

We are now in a position to prove Proposition 1 and Theorem 1.

##### Proof

Proof of Proposition 1 by Lemma 1

$$\begin{array}{l}{\mathbb{P}}\left({{{\rm{FDP}}}}(\theta )\ge (1+{{\Delta }}){q}_{+}\right)={\mathbb{P}}\left({\underline{Z}}_{\underline{k}(\theta )}\ge (1+{{\Delta }})\right)\\\qquad\qquad\qquad\qquad\qquad\quad \le {\mathbb{P}}\left(\mathop{\max }\limits_{k\le {k}_{\max }}{\underline{Z}}_{k}\ge (1+{{\Delta }})\right)\\\qquad\qquad\qquad\qquad\qquad\quad \mathop{\le }\limits^{(a)}{\mathbb{P}}\left(\mathop{\max }\limits_{k\le {k}_{\max }}{Z}_{k}\ge (1+{{\Delta }})\right)\\\qquad\qquad\qquad\qquad\qquad\quad \mathop{\le }\limits^{(b)}\frac{1}{1+{{\Delta }}}{\mathbb{E}}\{{Z}_{{k}_{\max }}\}\,,\end{array}$$

where (*a*) follows from Lemma 2 and (*b*) from Doob’s maximal inequality. Because (*Z*_*k*_) is a martingale, following the above:

$$\begin{array}{l}{\mathbb{P}}\left({{{\rm{FDP}}}}(\theta )\ge (1+{{\Delta }}){q}_{+}\right)\le \frac{1}{1+{{\Delta }}}{\mathbb{E}}\{{Z}_{{k}_{\max }}\}\\\qquad\qquad\qquad\qquad\qquad\quad =\frac{1}{1+{{\Delta }}}{\mathbb{E}}\{{Z}_{0}\}\\\qquad\qquad\qquad\qquad\qquad\quad =\frac{1}{1+{{\Delta }}}\cdot \frac{| N| }{| A| +1}\le \frac{1}{1+{{\Delta }}}\,.\end{array}$$

This proves the claim.

##### Proof

Proof of Theorem 1. By the same argument as in Lemma 1 (and adopting the standard notation ${\mathbb{P}}(A;B)={\mathbb{P}}(A\,{{\mbox{and}}}\,B)$):

$$\begin{array}{l}{\mathbb{P}}\left({{{\rm{FDP}}}}(\theta )\ge (1+{{\Delta }}){q}_{+}\,;\ M\ge m\right)={\mathbb{P}}\left({\underline{Z}}_{\underline{k}(\theta )}\ge (1+{{\Delta }})\,;\ M\ge m\right)\\\qquad\qquad\qquad\qquad\qquad\qquad\qquad\quad ={\mathbb{P}}\left({\underline{Z}}_{\underline{k}(\theta )}\ge (1+{{\Delta }})\,;\ \underline{k}(\theta )\le {k}_{\max }-m\right)\\\qquad\qquad\qquad\qquad\qquad\qquad\qquad\quad \mathop{\le }\limits^{(a)}{\mathbb{P}}\left({Z}_{\underline{k}(\theta )}\ge (1+{{\Delta }})\,;\ \underline{k}(\theta )\le {k}_{\max }-m\right)\\\qquad\qquad\qquad\qquad\qquad\qquad\qquad\quad \le {\mathbb{P}}\left(\mathop{\max }\limits_{k\le {k}_{\max }-m}{Z}_{k}\ge (1+{{\Delta }})\,;\ \underline{k}(\theta )\le {k}_{\max }-m\right)\\\qquad\qquad\qquad\qquad\qquad\qquad\qquad\quad \le {\mathbb{P}}\left(\mathop{\max }\limits_{k\le {k}_{\max }-m}{Z}_{k}\ge (1+{{\Delta }})\right)\,,\end{array}$$

where (*a*) follows from Lemma 2.

Letting $K:= {k}_{\max }-m$, for any non-negative, non-decreasing convex function $\psi :{{\mathbb{R}}}_{\ge 0}\to {{\mathbb{R}}}_{\ge 0}$

13 $$\begin{array}{ll}{\mathbb{P}}\left(\mathop{\max }\limits_{k\le K}{Z}_{k}\ge (1+{{\Delta }})\right)&={\mathbb{P}}\left(\mathop{\max }\limits_{k\le K}\psi ({Z}_{k})\ge \psi (1+{{\Delta }})\right)\\ &\mathop{\le }\limits^{(a)}\frac{{\mathbb{E}}\left\{\psi ({Z}_{K})\right\}}{\psi (1+{{\Delta }})}\,,\end{array}$$

where (*a*) follows from Doob’s inequality for the submartingale ${(\psi ({Z}_{k}))}_{k\ge 0}$.

Recalling the definition ${k}_{0}:= \min (k:\ {a}_{k}=0)$, we estimate the last expectation by

$$\begin{array}{l}{\mathbb{E}}\left\{\psi ({Z}_{K})\right\}\le {\mathbb{E}}\left\{\psi ({n}_{{k}_{0}}){{{{\boldsymbol{1}}}}}_{{k}_{0}\le K}\right\}+{\mathbb{E}}\left\{\psi \left(\frac{{n}_{K}}{{a}_{K}+1}\right){{{{\boldsymbol{1}}}}}_{{k}_{0}\ > \ K}\right\}\\\qquad\qquad\quad \le {\mathbb{E}}\left\{\psi (p){{{{\boldsymbol{1}}}}}_{{k}_{0}\le K}\right\}+{\mathbb{E}}\left\{\psi \left(\frac{{n}_{K}}{{a}_{K}+1}\right)\right\}\\\qquad\qquad\quad \le \psi (p){\mathbb{P}}({a}_{K}=0)+{\mathbb{E}}\left\{\psi \left(\frac{{n}_{K}}{{a}_{K}+1}\right){{{{\boldsymbol{1}}}}}_{| {n}_{K}-{\overline{n}}_{K}| \le \delta {\overline{n}}_{K}}{{{{\boldsymbol{1}}}}}_{| {a}_{K}-{\overline{a}}_{K}| \le \delta {\overline{a}}_{K}}\right\}\\\qquad\qquad\quad +\psi (p){\mathbb{P}}\left(| {n}_{K}-{\overline{n}}_{K}| > \delta {\overline{n}}_{K}\right)+\psi (p){\mathbb{P}}\left(| {a}_{K}-{\overline{a}}_{K}| > \delta {\overline{a}}_{K}\right)\\\qquad\qquad\quad \le {\mathbb{E}}\left\{\psi \left(\frac{{n}_{K}}{{a}_{K}+1}\right){{{{\boldsymbol{1}}}}}_{| {n}_{K}-{\overline{n}}_{K}| \le \delta {\overline{n}}_{K}}{{{{\boldsymbol{1}}}}}_{| {a}_{K}-{\overline{a}}_{K}| \le \delta {\overline{a}}_{K}}\right\}\\\qquad\qquad\quad +\psi (p){\mathbb{P}}\left(| {n}_{K}-{\overline{n}}_{K}| > \delta {\overline{n}}_{K}\right)+2\psi (p){\mathbb{P}}\left(| {a}_{K}-{\overline{a}}_{K}| > \delta {\overline{a}}_{K}\right)\,.\end{array}$$

Here, ${\overline{a}}_{K}={\mathbb{E}}[{a}_{K}]$, ${\overline{n}}_{K}={\mathbb{E}}[{n}_{K}]$, and *δ* is a small constant.

Let *X* ~ Binom(∣*N*∣, *ρ*), *Y* ~ Binom(∣*A*∣, *ρ*) be independent binomial random variables. Then, it is easy to see that, for any *ρ* ∈ (0, 1),

14 $${\mathbb{P}}({n}_{K}=r,{a}_{K}=s)={\mathbb{P}}\left(X=r,Y=s\left\vert \right.X+Y=m\right)\,,$$

15 $${\mathbb{E}}[{n}_{K}]=\frac{m| N| }{| A| +| N| }\,,\ \ \ \ {\mathbb{E}}[{a}_{K}]=\frac{m| A| }{| A| +| N| }\,.$$

In particular, because ∣*A*∣ = *p* and, by assumption, ∣*N*∣ ≥ *p*/2, we have $m/2\le {\mathbb{E}}[{a}_{K}]\le 2m/3$, $m/3\le {\mathbb{E}}[{n}_{K}]\le m/2$. Further choosing *ρ* = *m*/(∣*A*∣ + ∣*N*∣),

16 $${\mathbb{P}}\left(| {n}_{K}-{\overline{n}}_{K}| > \delta {\overline{n}}_{K}\right)=\frac{{\mathbb{P}}\left(| X-{\mathbb{E}}X| \ge \delta {\mathbb{E}}X;\ X+Y=m\right)}{{\mathbb{P}}\left(X+Y=m\right)}$$

17 $$\le C{m}^{1/2}{\mathbb{P}}\left(| X-{\mathbb{E}}X| \ge \delta {\mathbb{E}}X\right)$$

18 $$\le C{m}^{1/2}{e}^{-m({\delta }^{2}\wedge \delta )/C}\,,$$

where the first inequality follows by the local central limit theorem and the second by Bernstein inequality. Of course, a similar bound holds for *a*_*K*_.

Substituting above, we get, for *δ* < 1,

19 $${\mathbb{E}}\left\{\psi ({Z}_{K})\right\}\le {\mathbb{E}}\left\{\psi \left(\frac{{n}_{K}}{{a}_{K}+1}\right){{{{\boldsymbol{1}}}}}_{| {n}_{K}-{\overline{n}}_{K}| \le \delta {\overline{n}}_{K}}{{{{\boldsymbol{1}}}}}_{| {a}_{K}-{\overline{a}}_{K}| \le \delta {\overline{a}}_{K}}\right\}+C{m}^{1/2}\psi (p){e}^{-m{\delta }^{2}/C}\,.$$

Let ${n}_{K}:= (1+\eta ){\overline{n}}_{K}$ and ${a}_{K}:= (1+\alpha ){\overline{a}}_{K}$. For $| {n}_{K}-{\overline{n}}_{K}| \le \delta {\overline{n}}_{K}$, $| {a}_{K}-{\overline{a}}_{K}| \le \delta {\overline{a}}_{K}$, *δ* ≤ 1/4, we have

$$\begin{array}{ll}\frac{{n}_{K}}{{a}_{K}+1}&\le \frac{{\overline{n}}_{K}}{{\overline{a}}_{K}}\cdot \frac{1+\eta }{1+\alpha }\\ &\le \frac{| N| }{| A| }\cdot \left(1+\eta \right)\cdot \left(1-\alpha +2{\alpha }^{2}\right)\\ &\le 1+2| \eta | +2| \alpha | \,.\end{array}$$

We next choose $\psi (x)={(x-1)}_{+}^{\ell }$ for some *ℓ* ≥ 1 (this function is monotone and convex as required). We thus get, from (19), fixing *δ* = 1/4,

$$\begin{array}{ll}{\mathbb{E}}\left\{\psi ({Z}_{K})\right\}&\le {2}^{\ell }\,{\mathbb{E}}\left\{{\left(\frac{{n}_{K}-{\overline{n}}_{K}}{{\overline{n}}_{K}}+\frac{{a}_{K}-{\overline{a}}_{K}}{{\overline{a}}_{K}}\right)}_{+}^{\ell }\right\}+C{m}^{1/2}{p}^{\ell }{e}^{-m/C}\\ &\le {4}^{\ell }{\mathbb{E}}\left\{{\left(\frac{{n}_{K}-{\overline{n}}_{K}}{{\overline{n}}_{K}}\right)}^{\ell }\right\}+{4}^{\ell }{\mathbb{E}}\left\{{\left(\frac{{a}_{K}-{\overline{a}}_{K}}{{\overline{a}}_{K}}\right)}^{\ell }\right\}+C{m}^{1/2}{p}^{\ell }{e}^{-m/C}\\ &\le {4}^{\ell }\int\nolimits_{0}^{\infty }\left(1\wedge 2{e}^{-m(\delta \wedge {\delta }^{2})/C}\right){\delta }^{\ell -1}\,{{{\rm{d}}}}\delta +C{m}^{1/2}{p}^{\ell }{e}^{-m/C}\\ &\le {\left(\frac{C\ell }{m}\right)}^{\ell /2}+C{p}^{\ell +1}{e}^{-m/C}\,,\end{array}$$

where the last inequality holds for *ℓ* < *m*/*C* with *C* a sufficiently large constant. If we further choose $\ell \le m/(C\log p)$, then we get

$${\mathbb{E}}\left\{\psi ({Z}_{K})\right\}\le {\left(\frac{C\ell }{m}\right)}^{\ell /2}+C{e}^{-m/{C}^{{\prime} }}\le {\left(\frac{{C}^{{\prime}{\prime}}\ell }{m}\right)}^{\ell /2}\,.$$

Substituting in equation (13), we get, for any $q\le m/(C\log p)$:

$${\mathbb{P}}\left({{{\rm{FDP}}}}(\theta )\ge (1+{{\Delta }}){q}_{+}\,;\ M\ge m\right)\le {\left(\frac{{C}^{{\prime}{\prime}}\ell }{{{{\Delta }}}^{2}m}\right)}^{\ell /2}$$

Choosing *ℓ* = *c*_0_Δ^2^*m* for a sufficiently small constant *c*_0_ implies the claim.

### Comparison of algorithmic complexity

We compare the algorithmic complexity of the Lasso, EN, SS and Stabl algorithms:

- Lasso, EN and AL: Given the number of samples (*n*) and the number of features (*p*), the time complexity of the Lasso, EN or AL algorithm is $O(np\min \{n,p\})$ (refs. ^18,69^).
- SGL: Given the number of groups (*g*) and the average number of features in a group (*m*), the time complexity of the SGL would be $O(gmnp\min \{n,p\})$.
- SS: SS’s complexity depends on the number of subsamples (*B*) and the number of regularization parameters (*R*) considered. Assuming Lasso or EN is used as the base model, the time complexity of SS would be $O(BRnp\min \{n,p\})$.
- Stabl: Stabl’s complexity is driven by the base model (Lasso, EN, SGL or AL) and the additional steps introduced by the method. The time complexity of Stabl would be $O(BRn[p+{p}^{{\prime} }]\min \{n,p+{p}^{{\prime} }\})$ or $O(BRgmn[p+{p}^{{\prime} }]\min \{n,p+{p}^{{\prime} }\})$, where ${p}^{{\prime} }$ represents the number of artificial features introduced by Stabl’s method.

### Synthetic datasets

#### Gaussian models without correlation

We use a standard Gaussian covariates model^70,71^. Denoting the rows of ***X*** by *x*_1_, …*x*_*n*_, and the responses by *y*_1_, …, *y*_*n*_, we let the samples (*y*_*i*_, *x*_*i*_) be i.i.d. with:

20 $${x}_{i}={g}_{i}+t{z}_{i}\,,\ \ {g}_{i} \sim {{{\mathcal{N}}}}(0,{{{{\boldsymbol{I}}}}}_{p})\,,\ \ {z}_{i} \sim {{{\mathcal{N}}}}(0,{{{{\boldsymbol{\Sigma }}}}}_{Z})\,,$$

21 $${y}_{i}=\beta {z}_{i}^{\top }+{\epsilon }_{i}\,,\ \ \ {\epsilon }_{i} \sim {{{\mathcal{N}}}}(0,1)\,.$$

We use the following covariance and coefficients

22 $${{{{\boldsymbol{\Sigma }}}}}_{Z}={{{\rm{diag}}}}\left(\underbrace{{s}^{2},\ldots ,{s}^{2}}_{\begin{array}{c}k\end{array}},\underbrace{0,\ldots ,0}_{\begin{array}{c}p-k\end{array}}\right)\,,$$

23 $$\beta =\left(\underbrace{{\beta }_{1},\ldots ,{\beta }_{k}}_{\begin{array}{c}k\end{array}},\underbrace{0,\ldots ,0}_{\begin{array}{c}p-k\end{array}}\right)\,.$$

24 $$\forall i\le k,\,{\beta }_{i} \sim U(-10,10)$$

This structure was also used in ref. ^11^.

Note that the above can also be written in the standard form as

$${y}_{i}=b{x}_{i}^{\top }+{\tilde{\epsilon }}_{i},\ \ \ {x}_{i} \sim {{{\mathcal{N}}}}(0,{{{\boldsymbol{\Sigma }}}})\,,\ \ \ {\tilde{\epsilon }}_{i} \sim {{{\mathcal{N}}}}(0,{\sigma }^{2})\,,$$

where

25 $${{{\boldsymbol{\Sigma }}}}={{{\rm{diag}}}}\left(\underbrace{1+{s}^{2}{t}^{2},\ldots ,1+{s}^{2}{t}^{2}}_{\begin{array}{c}k\end{array}},1,\ldots ,1\right)\,,$$

26 $$b=\frac{t{s}^{2}}{1+{t}^{2}{s}^{2}}\beta \,,\ \ \ \ \ {\sigma }^{2}=1+\frac{{s}^{2}}{1+{t}^{2}{s}^{2}}\parallel \beta {\parallel }^{2}\,.$$

This distribution is parametrized by:

- Number of features *p*, number of informative features *k* and sample size *n*
- Variance parameters *s*, *t*
- *β* coefficients

Note that, for a binary outcome, we can use the new response ${p}_{i}={{{{\bf{1}}}}}_{S({y}_{i})\ge 0.5}$. S being the sigmoid function: $S(x)=\frac{1}{1+{\exp }^{-x}}$

#### Gaussian models with correlation

Following the procedure devised in the previous section, we simulate the Gaussian model with three levels of correlations. In this case, we use the same model as the previous section, but **Σ**_*Z*_ is a *k* × *k* matrix that captures the correlation among the informative features. We can define **Σ**_*Z*_ as:

27 $${{{{\boldsymbol{\Sigma }}}}}_{Z}=\left(\begin{array}{llll}{s}^{2}&{\rho }_{1}&\cdots \,&{\rho }_{k-1}\\ {\rho }_{1}&{s}^{2}&\cdots \,&{\rho }_{k-2}\\ \vdots &\vdots &\ddots &\vdots \\ {\rho }_{k-1}&{\rho }_{k-2}&\cdots \,&{s}^{2}\end{array}\right),$$

where *ρ*_1_, …, *ρ*_*k*−1_ are the correlation parameters for the informative features.

The coefficients *β* and the covariance matrix **Σ** used to generate the covariates *x*_*i*_ can be defined as before.

#### Non-Gaussian models

Although previous simulations were based on normally distributed data, omic data, such as bulk or single-cell RNA-seq datasets, often follow negative binomial and zero-inflated negative binomial distributions. The MX knockoff framework, despite its inherent adaptability to non-normal distributions, often requires modification based on the dataset’s specific nature and any existing model that describes the joint distribution of feature covariates^19,25^. For scenarios governed by a known data generation process, the MX knockoff framework was adjusted to generate artificial features. These features mirrored the marginal covariate distribution and correlation structure of non-normally distributed datasets. For these scenarios, the Stabl_SRM_ framework combined with MX knockoffs consistently enhanced sparsity and reliability in both regression and classification tasks (Extended Data Fig. 8 and Supplementary Table 2). In cases with undisclosed joint distribution, random permutations offer a viable option for generation of artificial features. Although ensuring genuine marginal distributions, this technique might not retain the dataset’s original correlation structure. However, the Stabl_SRM_’s results using random permutations paralleled those achieved with MX knockoffs on non-normally distributed datasets of varying correlation structures (Extended Data Fig. 8).

Collectively, synthetic modeling experiments underscore that the choice of base SRM and noise generation techniques within the Stabl_SRM_ framework can influence feature selection and model performance. Ideally, the correlation structure and data distribution should dictate this choice, but real-world datasets often have unknown true distributions. Therefore, selecting between MX knockoff or random permutation for artificial feature generation within the Stabl framework hinges on knowledge of covariate distribution and the analyst’s priority-preserving the original dataset’s correlation structure or its distribution.

##### Normal to Anything framework

Normal to Anything (NORTA) was designed to synthesize high-dimensional multivariate datasets^72–75^. This method can be used to generate random variables with arbitrary marginal distributions and correlation matrix from a multivariate normal distribution. In essence, the problem boils down to finding the pairwise correlations between the normal vectors that yield the desired correlation between the vectors of the non-normal distribution. In practice, this can be achieved using quantile functions. Using this method, we created correlated vectors following either a zero-inflated negative binomial model or a standard negative binomial model. Our simulations harnessed the capabilities of the Julia package Bigsimr, which implements this framework. This package enables data generation via the Gaussian copula (a joint distribution of the multivariate uniform vector obtained from the multivariate normal vector), facilitating the creation of datasets with targeted correlations and specified marginal distributions, such as Gaussian and negative binomials.

##### Negative binomial models

To generate the synthetic negative binomial models, we initially create a correlation matrix **Σ**_*Z*_ for the multivariate normal from which the copula is computed, and we verify that the informative features match the desired level of correlation (low (*ρ* = 0.2), intermediate (*ρ* = 0.5) and high (*ρ* = 0.7)).

We constructed *z*_*i*_ using this strategy and used the following parameters for the marginal distributions: NB(*μ* = 2, *ϕ* = 0.1).

Similar to the Gaussian cases, we then use the generated data to create the response with the following procedure:

2 $$\begin{array}{rc}&{y}_{i}=\beta {z}_{i}^{\top }+{\epsilon }_{i}\,,\ \ \ {\epsilon }_{i} \sim {{{\mathcal{N}}}}(0,1)\,.\end{array}$$

##### Zero-inflated negative binomial and normal models

To generate zero-inflated (ZI) covariates in our models, we follow a similar process as described earlier for the non-zero values in a negative binomial distribution or Gaussian distribution. Let *x*_*ij*_ represent the *j*-th covariate of the *i*-th observation. The ZI covariate can be generated as follows:

$$x^*_{ij} \sim \begin{cases} 0 & {\rm{with}}\,{\rm{probability}}\, \pi \\ x_{ij} & {\rm{with}}\, {\rm{probability}}\, (1 - \pi) \end{cases}$$

where *π* is the probability of observing a zero and is fixed in our examples at 0.1.

##### Adaptation of the MX knockoff with Gaussian copulas

In situations where the quantile–quantile transformation is available, we can easily adapt the MX knockoff procedure to generate knockoffs tailored to the chosen distribution. Specifically, from the synthetic data, we can estimate **Σ**_*Z*_ and generate MX knockoffs, thereby establishing the correspondence to the chosen distribution. For the sake of comparison with the random permutation procedure, we use this modified version of the knockoffs when we considered synthetic non-normal distributions.

#### Synthetic data for SGL

To apply SGL, the creation of predefined feature groups for analysis is needed. This was achieved through the construction of sets of five correlated covariates (*X*_*i*_). This was accomplished by generating a block diagonal correlation matrix (Σ_*Z*_) where, apart from the diagonal entries, all other elements were set to zero. This matrix was formulated to encapsulate the interrelationships solely within each covariate group. Specifically, the diagonal blocks, each of size five, represented distinct groups. By adopting this approach, we explicitly defined the covariate groups to be considered during the optimization process of the algorithm. This methodology remained consistent across various scenarios involving correlation structures and data distributions.

Let *X*_*i*_ denote the *i-*th group of correlated covariates, where *i* = 1, 2, …, *m* is the index of the group. The block diagonal correlation matrix Σ_*Z*_ is given by:

$${{{\Sigma }}}_{Z}=\left[\begin{array}{cccc}{{{\Sigma }}}_{Z1}&0&\ldots &0\\ 0&{{{\Sigma }}}_{Z2}&\ldots &0\\ \vdots &\vdots &\ddots &\vdots \\ 0&0&\ldots &{{{\Sigma }}}_{Zm}\end{array}\right]$$

Here, each Σ_*Zi*_ represents the correlation matrix among the covariates within group *X*_*i*_. By structuring Σ_*Z*_ in this way, we intentionally limit the relationships within each group and disregard correlations between different groups.

#### SS coupled with grid search

In this approach, we combined SS with grid search to optimize the threshold used to select the features. The procedure was as follows:

- We used a grid search method with a predefined number of possible thresholds ranging from 0% to 100%, evenly spaced across the range. This allowed us to test the sensitivity of SS performance to different thresholds.
- For each threshold, we applied the SS algorithm with the chosen threshold to select a subset of features. We then used this subset of features to train a logistic regression model.
- We used a CV method to compute the *R*^2^ score of the model for each threshold in the grid search on the training set.
- We selected the threshold that resulted in the highest *R*^2^ score as the optimal threshold.
- Finally, we used the selected threshold to predict the outcome variable on the test set using the logistic regression model trained on the full dataset with the selected subset of features.

#### Computational framework and pre-processing

Stabl was designed and executed using the Python packages ‘scikit-learn’ (version 1.1.2), ‘joblib’ (version 1.1.0) and ‘knockpy’ (version 1.2) (for the knockoff sampling generation). The Lasso algorithm fed into the Stabl subsampling process was executed using ‘scikit-learn’ (version 1.1.2) using the default threshold for feature selection at 10^−5^ in absolute value. The synthetic data generation was done using the Python package ‘numpy’ (version 1.23.1). Basic pre-processing steps, including variance thresholds and standardization, were executed using the Python packages ‘scikit-learn’ (version 1.1.2), ‘pandas’ (version 1.4.2) and ‘numpy’ (version 1.23.1). Visualization functions to plot stability path and FDR curves were executed using ‘seaborn’ (version 0.12.0) and ‘matplotlib’ (version 3.5.2).

#### Metrics on synthetic datasets

##### Predictive performance for binary classification

To evaluate our models, we use the AUROC and the area under the precision-recall curve (AUPRC) in the case of binary classification.

A common scale of performance was used to refer to the AUROC:

- 0.5–0.7 AUROC: modest performance
- 0.7–0.8 AUROC: good performance
- 0.8–0.9 AUROC: very good performance
- 0.9–1 AUROC: excellent performance

##### Predictive performance for regression

For regression tasks, the coefficient of determination *R*^2^, the RMSE and the mean absolute error (MAE) were used conventionally.

As for the AUROC, an arbitrary but common scale of performances was used in terms of *R*^2^ score:

- 0.0–0.3: No linear relationship
- 0.3–0.5: A weak linear relationship
- 0.5–0.7: A moderate linear relationship
- 0.7–0.9: A strong linear relationship
- 0.9–1.0: A very strong linear relationship

Note that, in some specific situations, the *R*^2^ score can be negative when the predictions are arbitrarily worse than using a constant value.

To assess the statistical significance of our results, we always performed a statistical test (two-sided Pearsonʼs *r*). To compare between methods, a two-sided Mann–Whitney rank-sum test was performed on the distribution of the repetition of the training for a given *n*.

##### Sparsity

Our measure of sparsity is the number of features that are selected in the final model. On the synthetic dataset, random samples are generated many times, so the average size of the set of selected features serves as our metric. To compare between methods, a two-sided Mann–Whitney rank-sum test was performed on the distribution of the repetition of the training for a given *n*.

##### Reliability

On the synthetic dataset, as we can sort out informative from uninformative features, we are able to compute the JI and the FDR, which are defined as:

$$\begin{array}{r}J=\frac{| \hat{S}\cap S| }{| \hat{S}\cup S| }\\ FDR=\frac{| \hat{S}\cap N| }{| \hat{S}| }\end{array}$$

The JI ranges from 0 (if no informative features are selected) to 1 (if the selected set comprises all informative features). To compare between methods, a two-sided Mann–Whitney rank-sum test was performed on the distribution of the repetition of the training for a given *n*.

### Benchmark on real-world datasets

#### Description of the datasets

##### PE dataset

The PE dataset contained cfRNA data previously collected as part of a prospective study of 49 pregnant women (29 with PE, 20 normotensive) receiving routine antenatal care at Lucile Packard Children’s Hospital at Stanford University. Blood samples were collected three times in pregnancy (early, mid and late pregnancy). Women were diagnosed as having PE following American College of Obstetrics and Gynecology^76^ guidelines. Women in the control group had uncomplicated term pregnancies. Samples collected from women who developed PE were collected before clinical diagnosis. The study was reviewed and approved by the institutional review board (IRB) at Stanford University (no. 21956). The details of the study design and the cfRNA sample preparation and data quality assessment were previously described^26,27^.

##### COVID-19 dataset

The analysis leveraged existing plasma proteomics data collected from 68 adults with a positive SARS-CoV-2 test (qRT–PCR on a nasopharyngeal swab specimen^29^). Publicly available plasma proteomic data using 784 SARS-CoV-2 samples from 306 positive patients was used for independent validation of the findings^28^. In the first study, 30 individuals reported having mild COVID-19 disease—that is, asymptomatic or various mild symptoms (for example, cough, fever, sore throat and loss of smell and taste) without any breathing issues. Thirteen individuals reported having moderate disease—that is, evidence of lower respiratory tract disease but with oxygen saturation (SpO_2_) above 94%. Twenty-five individuals were hospitalized with severe disease due to respiratory distress (SpO_2_ % ≥94%, respiratory frequency ≤30 breaths per minute, PaO_2_/FiO_2_ ≤300 mmHg or lung infiltrates ≥50%). For modeling purposes, COVID-19 severity was dummy-coded as follows: mild or moderate = 1 and severe = 2. The validation cohort consisted of 125 samples from patients with mild or moderate COVID-19 and 659 samples from patients with severe COVID-19. For both training and validation datasets, the Olink proximity extension assay (PEA, Olink Proteomics, Explore panel) was used to measure the plasma protein levels of 1,472 proteins^77^. Plasma was pre-treated with 1% Triton X-100 for 2 h at room temperature to inactivate the virus before freezing at −80 °C and shipping. The arbitrary unit normalized protein expression (NPX) is used to express the raw expression values obtained with the Olink assay, where high NPX values represent high protein concentration. Values were log_2_ transformed to account for heteroskedasticity.

##### Time-to-labor dataset

This dataset consisted of existing single-cell proteomic (mass cytometry), plasma proteomic and metabolomic data derived from the analysis of samples collected in a longitudinal cohort of pregnant women receiving routine antepartum and postpartum care at the Lucile Packard Children’s Hospital at Stanford University, as previously described^38^. The study was approved by the IRB of Stanford University (no. 40105), and all participants signed an informed consent form.

In brief, *n* = 63 study participants were enrolled in their second or third trimester of an uncomplicated, singleton pregnancy. Serial peripheral blood samples were collected at one to three times throughout pregnancy before the onset of spontaneous labor (the median sample size per patient is three).

In plasma, high-throughput untargeted mass spectrometry and an aptamer-based proteomic platform were used to quantify the concentration of 3,529 metabolites and 1,317 proteins, respectively. In whole blood, a 46-parameter mass cytometry assay measured a total of 1,502 single-cell immune features in each sample. These included the frequencies of 41 immune cell subsets (major innate and adaptive populations), their endogenous intracellular activities (phosphorylation states of 11 signaling proteins) and the capacities of each cell subset to respond to receptor-specific immune challenges (lipopolysaccharide (LPS), interferon-α (IFN-α), granulocyte macrophage colony-stimulating factor (GM-CSF) and a combination of IL-2, IL-4 and IL-6).

The original model to predict the time to onset of labor was trained on a cohort of *n* = 53 women with *n* = 150 samples. The independent validation of the model was performed on *n* = 10 additional pregnancies with *n* = 27 samples. A total of 6,348 immune, metabolite and protein features were included per sample. In this specific dataset, to account for the longitudinal nature of the data, we performed a patient shuffle split (PSS) method to assess the generalizability of our models. Specifically, we divided the dataset into two subsets and used one subset for training and the other for testing. Each subset contains all data from an individual patient (that is, for a given patient, its data are either in the training subset or the testing subset). We repeated this process *n* times, leaving out different patients (that is, all their data) each time. This approach allowed us to evaluate the performance of our models in predicting time to labor for patients not included in the training data. The dataset obtained was first *z*-scored, and the knockoff method was used for Stabl modeling experiments.

##### DREAM challenge dataset

The DREAM challenge study aimed at classifying PT and T labor pregnancies from vaginal microbiome data^48^. The DREAM challenge dataset contains nine publicly available and curated microbiome datasets with 1,569 samples, across 580 individuals (336 individuals delivered at T and 244 delivered PT). The DREAM challenge included 318 teams who submitted results for the classification of PT versus T pregnancies.

The MaLiAmPi pipeline was used to process all the data^48,49^. Essentially, DADA2 was used to assemble each project’s raw reads into approximate sequence variants (ASVs). These ASVs were then employed to recruit complete 16S rRNA gene alleles from a repository based on sequence similarity. The recruits were then assembled into a maximum-likelihood phylogeny using RAxM^78^, and the ASVs were placed onto this common phylogenetic tree through EPA-ng^79^. The final step was to use these placements to determine community alpha-diversity, phylogenetic (KR) distance between communities and taxonomic assignments for each ASV and to cluster ASVs into phylotypes based on their phylogenetic distance. Moreover, VALENCIA was used to identify each sample’s community state type (CST)^80^. MaLiAmPi is accessible as a nextflow workflow and is containerized at 100%, enabling it to be used on multiple high-performance computing resources.

Following the description for pre-processing of the best-performing team on the first challenge, we use specimens collected no later than 32 weeks of gestation to develop the prediction model. We extract microbiome data from phylotype_nreads.5e_1.csv, phylotype_nreads.1e0.csv, taxonomy_nreads.species.csv, taxonomy_nreads.genus.csv and taxonomy_nreads.family.csv tables. The phylotype_nreads.1e_1.csv table is not used because its number of columns (9,718) is overwhelming compared to the sample size.

We apply the centered log-ratio (clr) transformation^81^ on microbiome data to obtain scale-invariant values. In clr transformation, given a *D*-dimensional input *x*,

$$clr(x)=ln\left[\frac{{x}_{1}}{{g}_{m}(x)},\ldots ,\frac{{x}_{D}}{{g}_{m}(x)}\right]$$

where ${g}_{m}(x)={\left(\mathop{\prod }\nolimits_{i = 1}^{D}{x}_{i}\right)}^{\frac{1}{D}}$ is the geometric mean of *x*.

In this dataset, to account for the longitudinal nature of the data, we performed a PSS method to assess the generalizability of our models on the time-to-labor dataset.

##### SSI dataset

Patients undergoing non-urgent major abdominal colorectal surgery were prospectively enrolled between 11 July 2018 and 11 November 2020 at Stanford University Hospital after approval by the IRB of Stanford University and the obtaining of written informed consent (IRB-46978). Inclusion criteria were patients over 18 years of age who were willing and able to sign a written consent. Exclusion criteria were a history of inflammatory/autoimmune conditions not related to the indication for colorectal surgery as well as undergoing surgery that did not include resection of the bowel.

A nested case–control study was designed to identify pre-operative immunological factors predictive of the occurrence of an SSI. The study protocol was designed following the STROBE guidelines. The primary clinical endpoint was the occurrence of an SSI within 30 d of surgery, defined as superficial, deep or organ space SSI, anastomotic leak or dehiscence of the surgical incision. The primary clinical endpoint and all clinical variables were independently curated and validated by a colorectal surgeon and a practicing anesthesiologist. To minimize the effect of clinical and demographic variables potentially associated with the development of an SSI, patients who developed an SSI were matched to a control group of patients who did not develop an SSI. Patient characteristics and types of surgical procedures are provided in Supplementary Table 7. We performed a power analysis^82^ to determine the minimum required sample size of 80 patients to achieve an expected AUROC of 0.8, with a maximum 95% confidence interval (CI) of 0.25 and an expected SSI incidence of 25%. After conducting a frequency-matching procedure, we included a total of 93 patients, which reduced the expected confidence interval range to 0.23.

Whole blood and plasma samples were collected on the day of surgery (DOS) before induction of anesthesia, processed and analyzed following a similar workflow as previously described^53^. In brief, whole blood samples were either left unstimulated (to quantify cell frequency and endogenous cellular activities) or stimulated with a series of receptor-specific ligands eliciting key intracellular signaling responses implicated in the host’s immune response to trauma/injury, including LPS, TNFα and a combination of IL-2, IL-4 and IL-6. From each sample, 1,134 single-cell proteomic features were extracted using a 41-parameter single-cell mass cytometry immunoassay (Supplementary Table 11), including the frequency of 35 major innate and adaptive immune cells (Extended Data Fig. 10) and their intracellular signaling activities (for example, the phosphorylation state of 11 proteins). In addition, the plasma concentrations of 712 inflammatory proteins were quantified using the SOMAscan manual assay for human plasma^83,84^. SOMAscan kits were run in a SomaLogic trained and certified assay site. Mass cytometry data were collected using the default software for the CyTOF 3.0 Helios instrument (Helios CyTOF software, version 7.0.5189, Standard BioTools) and then gated using CellEngine (CellCarta).

### Stabl analysis of real-world datasets

For each real-world dataset, the dataset obtained was first *z*-scored, and the Stabl_SRM_ method was applied using Lasso, EN or AL as the base SRM (hyperparameters listed in Supplementary Table 13). To preserve the correlation structure of synthetic features, MX knockoffs served as the primary method for introducing noise in all omics datasets, except for the PE dataset (cfRNA). This dataset demonstrated the lowest internal correlation level (≤1% of features with intermediate or high correlations, *R* ≥ 0.5), and, therefore, random permutations were employed as the noise generation approach.

### Metrics on real-world datasets

#### Monte Carlo CV

The Monte Carlo CV is done as follows. At each fold, the dataset is split randomly into training and testing sets, and the model is then trained and evaluated using the training and testing sets, respectively:

- In the COVID-19 and SSI datasets, we executed Monte Carlo CV using the *RepeatedStratifiedKFold* class of ‘scikit-learn’ (version 1.1.2), which repeats the multiple K-fold CV scheme. We then take the median of the predictions to obtain the final predictions. This technique ensures that all samples are evaluated the same number of times. We used stratified five-fold CV (20% of the data are tested at each fold) to ensure that the class repartition was preserved among all the folds.
- In the time-to-labor, PE and DREAM datasets, we used the Monte Carlo CV with the *GroupShuffleSplit* class of ‘scikit-learn’ (version 1.1.2), allowing us to preserve the patients’ repartition between the training and testing sets as no patient’s samples are split into both sets. As before, the final predictions are obtained by taking the median of the predictions for each sample. The testing proportion was set at 20% at each fold.

#### Predictive performance

The predictive performance was measured using the same metrics as in the artificial datasets. The values were computed using the median from the Monte Carlo CV procedure for all the training cohorts. For the validation, the predictions from the final models were applied to compute the relevant metrics. When comparing predictive performance between methods, a two-sided bootstrap test was performed on the distribution of the CV folds.

#### Sparsity

Sparsity was defined as the average number of features selected in the model during the CV procedure. When comparing sparsity performance between methods, a two-sided Mann–Whitney rank-sum test was performed on the distribution of the CV folds.

#### Multi-omic modeling using Stabl and late-fusion Lasso

In early fusion, the features from different omics data are combined into a single feature set before training a model. This means that the model sees the combined feature set as a single input and learns a single set of weights for all the features. In contrast, late fusion involves training separate models on each omics data and then combining their predictions at the end. This can be done by taking the average of the predictions or by training a final model to combine the predictions from the separate models. Late fusion can be more flexible, allowing the different models to learn different weights for the features from each data source. Similarly to late fusion, Stabl adopts an independent analysis approach for each omic data layer by fitting specific reliability thresholds before selecting the most reliable features to be merged into a final layer. However, in contrast to late fusion, Stabl computes a specific reliability threshold for each omic data layer, allowing for the integration of the features selected from each omic data layer into a final modeling layer.

### Visualization

#### Uniform manifold approximation and projection

Uniform manifold approximation and projection (UMAP) is a dimensionality reduction technique that can be used to reduce the number of dimensions in a dataset while preserving the global structure of the data. UMAPs were plotted using the ‘umap-learn’ library and default parameters. The two first UMAP supports were used to represent all the molecular features in two-dimensional plots for all omics. The node sizes and colors were then calculated based on the intensity of the association with the outcome as the −log_10_ *P*value.

#### Stability paths

The stability path is used to visualize how the features are selected as the regularization parameter is varied. The stability path is a curve that plots the mean stability of each feature as a function of the regularization parameter. The stability of a feature is defined as the proportion of times that the feature is selected by the model when trained on different subsets of the data. The stability path can identify a range of regularization parameters that result in a stable set of features being selected.

#### Box plots

Throughout the figures, the box plots show the three-quartile values of the distribution along with extreme values. The whiskers extend to points that lie within 1.5× interquartile range (IQR) of the lower and upper quartile, and observations that fall outside this range are displayed independently.

#### ROC and PR curves

In the figures, the ROC and PR curves are displayed along with their CIs. The 95% CIs are computed with 2,000 stratified bootstrap replicates.

### Reporting summary

Further information on research design is available in the Nature Portfolio Reporting Summary linked to this article.

## Online content

Any methods, additional references, Nature Portfolio reporting summaries, source data, extended data, supplementary information, acknowledgements, peer review information; details of author contributions and competing interests; and statements of data and code availability are available at 10.1038/s41587-023-02033-x.

## Supplementary information

Supplementary Information Supplementary Tables 1–13.

Reporting Summary

## Data Availability

The datasets generated and/or analyzed during the current study are available on GitHub (https://github.com/gregbellan/Stabl/tree/main/Sample%20Data) and Dryad (10.5061/dryad.stqjq2c7d).

## Code availability

The Stabl framework and custom computer code used in this study can be accessed on GitHub (https://github.com/gregbellan/Stabl) and Zenodo (10.5281/zenodo.8406758).
