# Supplementary material for: Discovery of sparse, reliable omic biomarkers with Stabl
Source: Nat Biotechnol. Author manuscript; Available in PMC 2024 Oct 14. (PMC11217152; doi:10.1038/s41587-023-02033-x)
Supplement: supplemental_PMID38168992 [file NIHMS1960016-supplement-supplemental_PMID38168992.pdf]

	Number of lambdas	Number of bootstraps	Equivalent number of Lasso runs	Time to run (Stabl)
**PE – CFRNA (159 samples, 37184 features)**				
$Stabl_{L}$-RP	30	150	4 500	06 min 50 s
$Stabl_{AL}$-RP	30	150	4 500	10 min 06 s
$Stabl_{EN}$-RP	30	50	1 500	39 min 00 s
**COVID-19 – Proteomics (68, 1463)**				
$Stabl_{L}$-MX	30	1000	30 000	00 min 38 s
$Stabl_{L}$-RP	30	1000	30 000	00 min 38 s
$Stabl_{AL}$-MX	30	1000	30 000	00 min 47 s
$Stabl_{AL}$-RP	30	1000	30 000	00 min 47 s
$Stabl_{EN}$-MX	30	100	3 000	11 min 31 s
$Stabl_{EN}$-RP	30	100	3 000	11 min 27 s
**Onset of Labor – Proteomics (150, 1317)**				
$Stabl_{L}$-MX	30	1000	30 000	01 min 17 s
$Stabl_{L}$-RP	30	1000	30 000	01 min 12 s
$Stabl_{AL}$-MX	30	1000	30 000	01 min 34 s
$Stabl_{AL}$-RP	30	1000	30 000	01 min 25 s
$Stabl_{EN}$-MX	30	1000	30 000	01 min 10 s
$Stabl_{EN}$-RP	30	1000	30 000	01 min 08 s
**Onset of Labor – Metabolomics (150, 3529)**				
$Stabl_{L}$-MX	30	1000	30 000	04 min 11 s
$Stabl_{L}$-RP	30	1000	30 000	03 min 46 s
$Stabl_{AL}$-MX	30	1000	30 000	05 min 24 s
$Stabl_{AL}$-RP	30	1000	30 000	04 min 55 s
$Stabl_{EN}$-MX	30	1000	30 000	03 min 57 s
$Stabl_{EN}$-RP	30	1000	30 000	03 min 22 s
**Onset of Labor – CyTOF (150, 1502)**				
$Stabl_{L}$-MX	30	1000	30 000	01 min 04 s
$Stabl_{L}$-RP	30	1000	30 000	01 min 18 s
$Stabl_{AL}$-MX	30	1000	30 000	01 min 12 s
$Stabl_{AL}$-RP	30	1000	30 000	01 min 26 s
$Stabl_{EN}$-MX	30	1000	30 000	00 min 57 s
$Stabl_{EN}$-RP	30	1000	30 000	00 min 41 s
**Dream – Taxonomy (1569, 3725)**				
$Stabl_{L}$-MX	30	250	7 500	08 min 05 s
$Stabl_{L}$-RP	30	250	7 500	07 min 56 s
$Stabl_{AL}$-MX	30	250	7 500	12 min 35 s
$Stabl_{AL}$-RP	30	250	7 500	10 min 30 s
$Stabl_{EN}$-MX	15	50	750	51 min 19 s
$Stabl_{EN}$-RP	15	50	750	44 min 42 s
**Dream – Phylotype (1569, 5468)**				
$Stabl_{L}$-MX	30	250	7 500	06 min 13 s
$Stabl_{L}$-RP	30	250	7 500	06 min 40 s
$Stabl_{AL}$-MX	30	250	7 500	09 min 03 s
$Stabl_{AL}$-RP	30	250	7 500	08 min 38 s
$Stabl_{EN}$-MX	15	50	750	40 min 37 s
$Stabl_{EN}$-RP	15	50	750	36 min 39 s
**SSI – Proteomics (91, 721)**				
$Stabl_{L}$-MX	30	1000	30 000	00 min 29 s
$Stabl_{L}$-RP	30	1000	30 000	00 min 29 s
$Stabl_{AL}$-MX	30	1000	30 000	00 min 35 s
$Stabl_{AL}$-RP	30	1000	30 000	00 min 35 s
$Stabl_{EN}$-MX	30	1000	30 000	43 min 25 s
$Stabl_{EN}$-RP	30	1000	30 000	37 min 59 s
**SSI – CyTOF (93, 1125)**				
$Stabl_{L}$-MX	30	1000	30 000	00 min 30 s
$Stabl_{L}$-RP	30	1000	30 000	00 min 31 s
$Stabl_{AL}$-MX	30	1000	30 000	00 min 38 s
$Stabl_{AL}$-RP	30	1000	30 000	00 min 38 s
$Stabl_{EN}$-MX	30	1000	30 000	47 min 45 s
$Stabl_{EN}$-RP	30	1000	30 000	47 min 35 s

Supplementary Table S1 | Computation time and complexity for all clinical case studies. All benchmarks were launched using an EC2 instances on AWS (32 CPU, 128 GiB of ram).

Supplementary Table S2 | Benchmarking on synthetic data: comprehensive table of results. A comprehensive table numerically listing the results of all benchmark experiments on synthetic datasets and scenarios ($Stabl_{SRM}$ with four SRMs, tested on regression and classification tasks, three correlation structures, and four data distributions) are reported in a comprehensive and searchable online table. https://github.com/gregbellan/Stabl/blob/main/Extended%20Data%20Table%20S2.xlsx

		Stabl				Late fusion Lasso			
	n	Sparsity	Reliability (FDR)	Reliability (JI)	Predictivity	Sparsity	Reliability (FDR)	Reliability (JI)	Predictivity
	50	5 (1 - 9.8)	1 (0.9 - 1)	0 (0 - 0)	1.3 (1.2 - 1.3)	10.5 (1 - 28.5)	1 (0.8 - 1)	0 (0 - 0)	1.1 (1.1 - 1.2)
	75	31 (16.2 - 47.2)	1 (0.9 - 1)	0 (0 - 0.1)	1.3 (1.2 - 1.4)	8 (0 - 20)	0.9 (0 - 1)	0 (0 - 0)	1.1 (1.1 - 1.2)
	100	48.5 (32.2 - 97.5)	1 (0.9 - 1)	0 (0 - 0)	1.3 (1.3 - 1.6)	15 (1 - 27.5)	0.9 (0.8 - 1)	0 (0 - 0.1)	1.1 (1.1 - 1.1)
**Example 1**	150	29 (17 - 102.2)	0.9 (0.8 - 1)	0 (0 - 0.1)	1.2 (1.2 - 1.4)	30.5 (15.2 - 43.8)	0.9 (0.8 - 1)	0.1 (0 - 0.1)	1.1 (1.1 - 1.1)
	200	26 (18 - 127.2)	0.9 (0.9 - 1)	0 (0 - 0.1)	1.2 (1.2 - 1.4)	52.5 (40.2 - 64)	0.9 (0.8 - 0.9)	0.1 (0.1 - 0.1)	1.1 (1 - 1.1)
**Omic 1 (500,40)**	300	33.5 (20.2 - 135)	0.8 (0.8 - 1)	0.1 (0 - 0.1)	1.1 (1 - 1.2)	80.5 (64.8 - 96.5)	0.8 (0.8 - 0.9)	0.1 (0.1 - 0.1)	1 (1 - 1)
**Omic 2 (500,5)**	400	27.5 (19.5 - 42.5)	0.8 (0.7 - 0.9)	0.1 (0.1 - 0.2)	1 (1 - 1.1)	98.5 (78 - 123)	0.9 (0.8 - 0.9)	0.1 (0.1 - 0.2)	0.9 (0.9 - 1)
**Omic 3 (500,5)**	600	28.5 (25 - 35.5)	0.7 (0.6 - 0.7)	0.2 (0.2 - 0.2)	0.9 (0.8 - 0.9)	106.5 (93.8 - 136.5)	0.8 (0.8 - 0.9)	0.1 (0.1 - 0.2)	0.9 (0.9 - 0.9)
	800	32 (29 - 39.5)	0.6 (0.6 - 0.7)	0.3 (0.2 - 0.3)	0.8 (0.8 - 0.8)	133 (111 - 151.8)	0.9 (0.8 - 0.9)	0.1 (0.1 - 0.2)	0.9 (0.9 - 0.9)
	1000	31.5 (28.2 - 39)	0.6 (0.5 - 0.7)	0.3 (0.3 - 0.3)	0.8 (0.7 - 0.8)	126.5 (109.5 - 137.5)	0.8 (0.8 - 0.9)	0.2 (0.1 - 0.2)	0.9 (0.9 - 0.9)
	50	4 (2 - 10)	1 (0.9 - 1)	0 (0 - 0)	1.3 (1.2 - 1.4)	10.5 (1 - 25.8)	1 (0.8 - 1)	0 (0 - 0)	1.1 (1.1 - 1.2)
	75	37 (23.2 - 50.8)	1 (0.9 - 1)	0 (0 - 0)	1.3 (1.3 - 1.5)	11 (3.2 - 31.2)	0.9 (0.9 - 1)	0 (0 - 0)	1.1 (1.1 - 1.2)
	100	43.5 (24.8 - 95.5)	0.9 (0.9 - 1)	0 (0 - 0.1)	1.3 (1.2 - 1.6)	17 (4.5 - 28)	0.9 (0.9 - 1)	0 (0 - 0)	1.1 (1.1 - 1.1)
**Example 2**	150	28 (15 - 51.8)	0.9 (0.9 - 0.9)	0.1 (0 - 0.1)	1.2 (1.2 - 1.3)	21 (10.2 - 38.5)	0.9 (0.8 - 0.9)	0 (0 - 0.1)	1.1 (1.1 - 1.1)
	200	21 (15.5 - 44.8)	0.9 (0.8 - 0.9)	0.1 (0 - 0.1)	1.2 (1.1 - 1.2)	35 (11 - 49.8)	0.9 (0.8 - 0.9)	0.1 (0.1 - 0.1)	1.1 (1.1 - 1.1)
**Omic 1 (500,20)**	300	23 (16 - 33)	0.8 (0.7 - 0.9)	0.1 (0.1 - 0.2)	1.1 (1 - 1.1)	65 (42.2 - 83.8)	0.9 (0.8 - 0.9)	0.1 (0.1 - 0.1)	1.1 (1.1 - 1.1)
**Omic 2 (500,15)**	400	17 (15 - 22.8)	0.7 (0.6 - 0.8)	0.2 (0.1 - 0.2)	1 (0.9 - 1)	89 (75.2 - 106.8)	0.9 (0.8 - 0.9)	0.1 (0.1 - 0.1)	1.1 (1 - 1.1)
**Omic 3 (500,15)**	600	21 (19 - 24.8)	0.6 (0.6 - 0.7)	0.2 (0.2 - 0.2)	0.9 (0.8 - 0.9)	115.5 (86.5 - 138.2)	0.9 (0.8 - 0.9)	0.1 (0.1 - 0.1)	1 (1 - 1)
	800	24 (22.2 - 27)	0.6 (0.6 - 0.6)	0.3 (0.2 - 0.3)	0.8 (0.8 - 0.8)	112.5 (101.2 - 137.8)	0.8 (0.8 - 0.9)	0.1 (0.1 - 0.2)	1 (1 - 1)
	1000	26 (23 - 28)	0.6 (0.5 - 0.6)	0.3 (0.3 - 0.3)	0.8 (0.8 - 0.8)	125.5 (109.8 - 147.2)	0.9 (0.8 - 0.9)	0.1 (0.1 - 0.2)	1 (1 - 1)
	50	15 (8 - 27.2)	1 (0.9 - 1)	0 (0 - 0)	1.3 (1.3 - 1.4)	4 (1.2 - 20.2)	1 (0.9 - 1)	0 (0 - 0)	1.1 (1.1 - 1.2)
	75	26 (10.8 - 43.8)	1 (0.9 - 1)	0 (0 - 0)	1.3 (1.3 - 1.4)	11.5 (3 - 26.2)	0.9 (0.8 - 1)	0 (0 - 0)	1.1 (1.1 - 1.2)
	100	42.5 (25.2 - 84)	0.9 (0.9 - 1)	0 (0 - 0)	1.3 (1.2 - 1.5)	7 (1 - 23.8)	0.9 (0.6 - 1)	0 (0 - 0)	1.1 (1.1 - 1.1)
**Example 3**	150	23 (16.2 - 53)	0.9 (0.9 - 0.9)	0 (0 - 0.1)	1.2 (1.2 - 1.3)	18.5 (9 - 39.8)	0.9 (0.9 - 0.9)	0 (0 - 0.1)	1.1 (1.1 - 1.1)
	200	21.5 (14.5 - 73)	0.9 (0.8 - 0.9)	0.1 (0 - 0.1)	1.2 (1.1 - 1.2)	28 (12.8 - 53.2)	0.9 (0.8 - 0.9)	0.1 (0.1 - 0.1)	1.1 (1.1 - 1.1)
**Omic 1 (900,30)**	300	21 (17 - 28.8)	0.8 (0.7 - 0.8)	0.1 (0.1 - 0.1)	1.1 (1 - 1.1)	75 (51 - 88)	0.9 (0.9 - 0.9)	0.1 (0.1 - 0.1)	1.1 (1 - 1.1)
**Omic 2 (300,10)**	400	23 (18 - 28)	0.7 (0.6 - 0.8)	0.1 (0.1 - 0.2)	1 (1 - 1)	100.5 (77.2 - 115)	0.9 (0.8 - 0.9)	0.1 (0.1 - 0.1)	1 (1 - 1)
**Omic 3 (300,10)**	600	24 (19.2 - 25.8)	0.6 (0.6 - 0.7)	0.2 (0.2 - 0.2)	0.9 (0.8 - 0.9)	114 (92.5 - 149.8)	0.9 (0.8 - 0.9)	0.1 (0.1 - 0.1)	1 (1 - 1)
	800	26 (22.2 - 32.5)	0.6 (0.6 - 0.6)	0.3 (0.2 - 0.3)	0.8 (0.8 - 0.8)	149.5 (107.8 - 161.8)	0.9 (0.9 - 0.9)	0.1 (0.1 - 0.1)	1 (0.9 - 1)
	1000	27 (25 - 30)	0.6 (0.6 - 0.6)	0.3 (0.2 - 0.3)	0.8 (0.8 - 0.8)	140.5 (126.2 - 149.5)	0.9 (0.9 - 0.9)	0.1 (0.1 - 0.1)	0.9 (0.9 - 0.9)
	50	16.5 (11.2 - 32.5)	0.9 (0.9 - 1)	0 (0 - 0)	1.3 (1.3 - 1.4)	8.5 (3 - 27.5)	0.9 (0.9 - 1)	0 (0 - 0)	1.1 (1.1 - 1.2)
	75	15.5 (10.2 - 45.8)	0.9 (0.9 - 1)	0 (0 - 0.1)	1.3 (1.2 - 1.4)	13.5 (2 - 27.2)	0.9 (0.9 - 1)	0 (0 - 0)	1.1 (1.1 - 1.2)
	100	39 (23.2 - 79)	0.9 (0.9 - 0.9)	0.1 (0 - 0.1)	1.3 (1.2 - 1.5)	11.5 (2.2 - 31)	0.9 (0.7 - 0.9)	0 (0 - 0.1)	1.1 (1.1 - 1.1)
**Example 4**	150	33 (18.2 - 54)	0.9 (0.9 - 0.9)	0.1 (0 - 0.1)	1.2 (1.2 - 1.2)	37 (18 - 60.8)	0.9 (0.9 - 0.9)	0.1 (0 - 0.1)	1.1 (1.1 - 1.1)
	200	28.5 (12.5 - 71.5)	0.8 (0.8 - 0.9)	0.1 (0.1 - 0.1)	1.1 (1.1 - 1.2)	45 (21 - 57.8)	0.9 (0.8 - 0.9)	0.1 (0.1 - 0.1)	1.1 (1.1 - 1.1)
**Omic 1 (900,10)**	300	26.5 (19 - 38)	0.8 (0.7 - 0.9)	0.1 (0.1 - 0.1)	1.1 (1 - 1.1)	65.5 (50.2 - 86.5)	0.9 (0.8 - 0.9)	0.1 (0.1 - 0.1)	1 (1 - 1.1)
**Omic 2 (300,20)**	400	20.5 (18 - 30.2)	0.7 (0.7 - 0.8)	0.1 (0.1 - 0.2)	1 (0.9 - 1)	95 (74.8 - 111.5)	0.9 (0.8 - 0.9)	0.1 (0.1 - 0.1)	1 (1 - 1)
**Omic 3 (300,20)**	600	24 (21.2 - 27.8)	0.6 (0.6 - 0.7)	0.2 (0.2 - 0.2)	0.9 (0.8 - 0.9)	104 (86 - 120.5)	0.9 (0.8 - 0.9)	0.1 (0.1 - 0.2)	1 (1 - 1)
	800	25 (24 - 28.8)	0.6 (0.6 - 0.6)	0.3 (0.2 - 0.3)	0.8 (0.8 - 0.8)	112 (94.2 - 131.8)	0.9 (0.8 - 0.9)	0.1 (0.1 - 0.2)	1 (0.9 - 1)
	1000	28.5 (26 - 30.8)	0.6 (0.5 - 0.6)	0.3 (0.3 - 0.3)	0.8 (0.8 - 0.8)	104 (96.5 - 131)	0.8 (0.8 - 0.9)	0.1 (0.1 - 0.2)	0.9 (0.9 - 0.9)
	50	14 (6.5 - 28.5)	0.9 (0.9 - 1)	0 (0 - 0)	1.4 (1.3 - 1.4)	14.5 (1.2 - 27)	0.9 (0.9 - 1)	0 (0 - 0)	1.1 (1.1 - 1.2)
	75	24 (14 - 51.2)	0.9 (0.9 - 1)	0 (0 - 0.1)	1.3 (1.3 - 1.5)	16.5 (1.2 - 29.5)	0.9 (0.9 - 1)	0 (0 - 0)	1.1 (1.1 - 1.2)
	100	34.5 (21 - 47.8)	0.9 (0.9 - 0.9)	0 (0 - 0.1)	1.3 (1.2 - 1.3)	8 (1.2 - 16.5)	0.9 (0.6 - 1)	0 (0 - 0.1)	1.1 (1.1 - 1.1)
**Example 5**	150	38 (27 - 71.2)	0.9 (0.9 - 0.9)	0.1 (0 - 0.1)	1.2 (1.2 - 1.3)	25 (11.2 - 40)	0.9 (0.9 - 0.9)	0.1 (0 - 0.1)	1.1 (1.1 - 1.1)
	200	26 (19 - 49.2)	0.9 (0.8 - 0.9)	0.1 (0.1 - 0.1)	1.1 (1.1 - 1.2)	39.5 (16.2 - 56.2)	0.9 (0.8 - 0.9)	0.1 (0.1 - 0.1)	1.1 (1.1 - 1.1)
**Omic 1 (1000,20)**	300	24 (19 - 30.2)	0.8 (0.7 - 0.8)	0.1 (0.1 - 0.2)	1 (1 - 1.1)	65 (48 - 90.8)	0.9 (0.8 - 0.9)	0.1 (0.1 - 0.1)	1.1 (1 - 1.1)
**Omic 2 (250,15)**	400	20 (17 - 27.2)	0.7 (0.6 - 0.7)	0.2 (0.1 - 0.2)	1 (0.9 - 1)	79 (62.5 - 94.8)	0.9 (0.8 - 0.9)	0.1 (0.1 - 0.1)	1 (1 - 1.1)
**Omic 3 (250,15)**	600	23 (20 - 25.8)	0.6 (0.6 - 0.7)	0.2 (0.2 - 0.2)	0.9 (0.9 - 0.9)	100.5 (90 - 124.8)	0.9 (0.8 - 0.9)	0.1 (0.1 - 0.1)	1 (1 - 1)
	800	25 (22 - 27)	0.6 (0.6 - 0.6)	0.2 (0.2 - 0.3)	0.8 (0.8 - 0.8)	115 (103.5 - 148.2)	0.9 (0.8 - 0.9)	0.1 (0.1 - 0.2)	1 (1 - 1)
	1000	26 (25 - 29)	0.6 (0.5 - 0.6)	0.3 (0.3 - 0.3)	0.8 (0.8 - 0.8)	117 (108 - 127.5)	0.9 (0.8 - 0.9)	0.1 (0.1 - 0.1)	1 (1 - 1)

Supplementary Table S3 | Stabl$_L$'s performance on synthetic multi-omic data compared to Lasso. Sparsity ($|\hat{S}|$), reliability (FDR and JI), and predictivity (RMSE) of Stabl$_L$ and late fusion Lasso are represented as median (IQR) for five examples of synthetic triple-omic datasets with 1450 uninformative and 50 informative features each (1500 features total).

Case study	PE	COVID	OOL			SSI		Dream	
Omic	CFRNA	Proteomics	Proteomics	Metabolomics	CyTOF	Proteomics	CyTOF	Taxonomy	Phylotype
% Corr. > 0.7	<1%	8%	<1%	<1%	3%	1%	2%	18%	5%
% Corr. > 0.5	<1%	16%	2%	2%	7%	3%	7%	35%	11%
% Corr. > 0.2	13%	46%	20%	16%	35%	24%	37%	69%	41%

Supplementary Table S4 | Correlation structure of all clinical case study datasets.

	Predictivity			Sparsity			
	SRM	Stabl$_{SRM}$-MX	Stabl$_{SRM}$-RP	SRM	Stabl$_{SRM}$-MX	Stabl$_{SRM}$-RP	p-value SRM vs. Stabl$_{SRM}$-MX (RP)
**SSI**							
Lasso	0.722 [0.580, 0.838]	0.818 [0.713, 0.903]	NA	61 [43.25, 83.25]	28.5 [20, 36]	NA	< 1e-16
ALasso	0.757 [0.631, 0.876]	0.804 [0.703, 0.890]	NA	33 [25, 46]	24 [19, 30]	NA	3.03E-06
EN	0.752 [0.630, 0.860]	0.779 [0.677, 0.882]	NA	304.5 [186, 449.25]	30.5 [21.75, 48]	NA	< 1e-16
**COVID (Training cohort)**							
Lasso	0.858 [0.745, 0.952]	0.834 [0.718, 0.920]	0.847 [0.736, 0.935]	17.5 [8, 107.25]	9 [4, 17]	7 [4.75, 13]	1.84E-07 (4e-10)
ALasso	0.863 [0.750, 0.948]	0.763 [0.638, 0.887]	NA	7.5 [3, 18.25]	6 [3, 8]	NA	6.89E-05
EN	0.878 [0.771, 0.954]	0.865 [0.767, 0.951]	NA	473 [22.25, 545]	16.5 [6, 63]	NA	3.89E-14
**COVID (Validation cohort)**							
Lasso	0.759 [0.708, 0.806]	0.749 [0.704, 0.790]	0.748 [0.706, 0.789]				
ALasso	0.741 [0.690, 0.788]	0.758 [0.719, 0.802]	NA				
EN	0.765 [0.715, 0.811]	0.734 [0.678, 0.785]	NA				
**PE**							
Lasso	0.842 [0.781, 0.902]	NA	0.828 [0.759, 0.892]	251.5 [150.25, 358.25]	NA	11 [8, 17]	< 1e-16
ALasso	0.833 [0.761, 0.895]	NA	0.799 [0.730, 0.870]	57 [38.5, 87.5]	NA	7 [5, 11]	< 1e-16
EN	0.830 [0.759, 0.893]	NA	0.859 [0.780, 0.927]	1576 [290.5, 1690.25]	NA	26 [15.5, 38]	< 1e-16
**Dream**							
Lasso	0.713 [0.686, 0.738]	0.687 [0.658, 0.716]	0.685 [0.663, 0.707]	638 [605.75, 675.25]	16 [13.75, 22]	28 [23.25, 32]	< 1e-16 (< 1e-16)
ALasso	0.710 [0.682, 0.738]	0.647 [0.617, 0.675]	NA	367 [336, 413.25]	10 [8, 13]	NA	< 1e-16
EN	0.729 [0.703, 0.754]	0.685 [0.659, 0.713]	NA	3470 [3105.25, 3757]	25 [20, 36.75]	NA	< 1e-16
**OOL (Training cohort)**							
Lasso	14.659 [11.998, 17.823]	15.033 [11.541, 19.063]	14.949 [11.955, 18.351]	194.5 [172, 218.25]	25 [20.75, 38.75]	25 [22 - 29]	< 1e-16 (< 1e-16)
ALasso	14.885 [12.291, 17.814]	14.954 [11.336, 19.139]	NA	133 [106, 162]	25 [21, 36.25]	NA	< 1e-16
EN	14.908 [12.417, 17.408]	17.273 [12.758, 23.093]	NA	470.5 [346, 590.5]	98 [55.75, 196.75]	NA	< 1e-16
**OOL (Validation cohort)**							
Lasso	19.199 [13.933, 24.777]	20.882 [16.567, 24.921]	21.791 [16.703, 26.196]				
ALasso	20.068 [15.404, 24.294]	20.882 [16.950, 24.849]	NA				
EN	19.088 [12.852, 25.003]	24.934 [19.905, 29.488]	NA				

Supplementary Table S5 | Benchmarking on clinical case studies: comprehensive table of results. Significance of sparsity performances was calculated using a two-sided Mann Whitney test.

PE	Predictivity (ROC AUC)	Predictivity (PR AUC)	Sparsity (number of features)
$Stabl_L$-RP	0.829 [0.755, 0.898]	0.850 [0.766, 0.931]	11.000 [7.750, 16.000]
SS (threshold 30%)	0.832 [0.764, 0.894] (p=0.383)	0.862 [0.778, 0.930] (p=0.281)	10.500 [9.000, 12.250] (p=4.683e-01)
SS (threshold 50%)	0.777 [0.694, 0.841] (p=0.014)	0.856 [0.783, 0.913] (p=0.424)	2.000 [2.000, 3.000] (p=6.495e-33)
SS (threshold 80%)	0.464 [0.432, 0.486] (p=0.0)	0.586 [0.507, 0.667] (p=0.0)	0.000 [0.000, 0.250] (p=3.266e-36)

COVID-19 (Training cohort)	Predictivity (ROC AUC)	Predictivity (PR AUC)	Sparsity (number of features)
$Stabl_L$-RP	0.847 [0.736, 0.935]	0.736 [0.543, 0.918]	7.000 [4.750, 13.000]
SS (threshold 30%)	0.851 [0.746, 0.939] (p=0.463)	0.739 [0.577, 0.923] (p=0.488)	3.000 [2.000, 4.000] (p=4.864e-18)
SS (threshold 50%)	0.491 [0.442, 0.532] (p=0.0)	0.364 [0.254, 0.471] (p=0.0)	0.000 [0.000, 1.000] (p=1.026e-35)
SS (threshold 80%)	0.500 [0.500, 0.500] (p=0.0)	0.368 [0.250, 0.485] (p=0.0)	0.000 [0.000, 0.000] (p=5.108e-39)

COVID-19 (Validation cohort)	Predictivity (ROC AUC)	Predictivity (PR AUC)
$Stabl_L$-RP	0.748 [0.706, 0.789]	0.932 [0.912, 0.952]
SS (threshold 30%)	0.778 [0.734, 0.820] (p=0.056)	0.946 [0.930, 0.962] (p=0.011)
SS (threshold 50%)	0.722 [0.670, 0.768] (p=0.252)	0.925 [0.901, 0.947] (p=0.289)
SS (threshold 80%)	0.500 [0.500, 0.500] (p=0.0)	0.841 [0.814, 0.867] (p=0.0)

Supplementary Table S6 | $Stabl_L$'s performance on single-omic datasets compared to SS. Significance of sparsity performances was calculated using a two-sided Mann Whitney test, significance of predictivity performances was calculated using permutation testing.

Features	Coefficients
CDK10	0.11
MT-TM	-1.34
MT-TH	2.43
HNRNPA3P6	-0.81
RPL34P34	-0.71
YWHAQP5	0.46
RPL23AP7	-1.45
GOLGA5P1	-0.76
MTRNR2L8	0.01

Supplementary Table S7 | Features selected by $Stabl_L$ for clinical case study 1: PE.

Features	Coefficients
CCL20	1.74
CRTAC1	-0.66
MDGA1	-2.03
MZB1	2.93

Supplementary Table S8 | Features selected by Stabl$_L$ for clinical case study 2: COVID-19.

Features	Coefficients
Granulocytes_S6_unstim	-3.18
Granulocytes_CREB_GMCSF	-1.65
Granulocytes_STAT1_IFNa	-2.90
NK_STAT1_IFNa	17.86
CD69negCD56loCD16negNK_STAT1_IFNa	-8.28
CD56hiCD16negNK_MAPKAPK2_IL246	1.12
Bcells	-0.91
CCR5posCCR2posCD4Tem	4.46
NKT	2.59
Granulocytes	0.75
Angiopoietin.2	-1.61
Siglec.6	4.09
Activin.A	4.28
IL.1.R4	2.03
SLPI	2.66
MMP.12	-3.65
PLXB2	-1.42
X147.0663_4.7	0.98
X193.0618_5.3	3.34
X331.2264_8.1	0.80
X335.2226_9.3	-2.79
X349.2371_7.5	2.32
X367.1495_0.8	1.37
X371.1895_10.6	3.14
X441.264_9.5	1.28
X443.208_8.4	7.43
X461.2387_8.7	-1.76

Supplementary Table S9 | Features selected by Stabl$_L$ for clinical case study 3: Time to labor.

Patient characteristic	No Surgical Site Infection, 83% (n=77)	Surgical Site Infection, 17% (n=16)
Age (mean + SD)	59.2 ±13.9	58.8 ± 14.2
Male, % (n)	49 (38)	50 (8)
Ethnicity, black, % (n)	1 (1)	0 (0)
BMI	28.3 ± 6.5	26.6 ± 4.6
**Surgical indication**		
Cancer	58% (45)	25% (4)
Inflammatory bowel disease	6% (5)	12.5% (2)
Other	35% (27)	62.5% (10)
**Type of surgery**		
Colectomy	64% (49)	56% (9)
Small bowels	3% (2)	6% (1)
Other	34% (26)	37% (6)
**Surgical approach**		
Minimally invasive	32% (25)	32% (5)
Open surgery	68% (52)	68% (11)
Operative duration, min, mean±SD	213 ±132	234 ±111
ASA classification, median	3	2.5

Supplementary Table S10 | Clinical information for clinical case study 4: SSI.

Target	Clone	Supplier	Catalog #	Concentration (µg/ml)	Comment
CD235ab	HIR2	Biolegend	306615	1	
CD61	VI-PL2	BD	555752	0.5	
CD45	HI30	Biolegend	304045	1	
CD66	CD66a-B1.1	BD	551354	1	
CD7	M-T701	BD	555359	1	
CD19	HIB19	Biolegend	302247	1	
CD45RA	HI100	Biolegend	304143	1	
CD11b	ICRF44	Biolegend	301337	2	
CD4	RPA-T4	Biolegend	300541	2	
CD8a	RPA-T8	BD	557084	1	
CD11c	Bu15	Biolegend	337221	1	
CD123	6H6	Biolegend	306027	1	
TCRγδ	B1	BD	555715	4	
FceRIα	AER-37 (CRA-1)	Biolegend	334602	0.5	
CD161	HP-3G10	Biolegend	339919	4	
CD33	WM53	Biolegend	303419	2	
CRTH2	BM16	Biolegend	350102	4	
CD16	3G8	Biolegend	302051	2	
CD25	M-A251	Biolegend	356102	2	
CD3	UCHT1	Biolegend	300443	1	
CXCR4	12G5	Biolegend	306502	4	
CD62L	DREG.200	Thermo Fisher	BMS1015	0.5	
CCR2	K036C2	Biolegend	357202	2	
HLA-DR	L243	Biolegend	307651	2	
CD14	M5E2	Biolegend	301843	4	
CD56	NCAM16.2	BD	559043	1	
OLFM4	hOLFM4	Matt Alder (CCHMC)	-	4	Received as a gift
pCREB	87G3	CST	9198	2	Custom synthesis
pSTAT5	C11C5	CST	51879SF	4	
pp38	36/p38	BD	612281	2	Custom synthesis
pSTAT1	14/P-STAT1	BD	612132	1	Custom synthesis
pSTAT3	M9C6	CST	74309SF	4	
pS6	D57.2.2E	CST	4858	2	Custom synthesis
pMAPKAPK2	27B7	CST	3007	1	Custom synthesis
Tbet	4B10	Thermo Fisher	14-5825-82	8	
cPARP	F21-852	BD	552597	2	
FoxP3	PCH101	Thermo Fisher	14-4776-82	10	
IκB	L35A5	CST	4814	8	Custom synthesis
pNFκB	K10-895.12.50	BD	558393	2	
pERK1/2	D13.14.4E	CST	45899SF	2	
pSTAT6	A15137E	Biolegend	686002	2	

Supplementary Table S11 | Antibody panel for clinical case study 4: SSI.

Features	Coefficients
unstim_CD4Trm_Frequency	4.90
unstim_Tregnaive_Frequency	0.84
IL246_Granulocytes_154Sm_STAT3	-0.02
IL246_Th1naive_153Eu_STAT1	1.89
SAMHD1	2.69
ARL11	3.28
LDLR	3.37
RNASET2	1.65
HSPH1	2.22
WWOX	2.82
MTHFD2	0.67
OGG1	-4.60
ITGAV|ITGB3	-2.78
CNTN3	-0.17
CTNNA3	1.35
IL1B	3.68
CCL3	3.69
FCGR1A	1.88
MAPK8	2.49
CSF2	1.16
PPIB	-0.31
BIRC3	-2.88
IL18	1.92
ASAH1	1.93
ALK	3.04
TNFSF10	1.77
RAP2A	0.37
TOPBP1	0.75
IRF6	0.15

Supplementary Table S12 | Features selected by Stabl$_L$ for clinical case study 4: SSI.

	CFRNA	COVID-19	OOL	Biobank	Dream
**Preprocessing**					
**Variance threshold**	0.01	0.01	0.01	0.01	0.01
**Low info filter**	0.2	0.2	0.2	0.2	0.2
**Simple Imputer**	median	median	median	median	median
**StandardScaler**	default	default	default	default	default
**Class weight**	Balanced	Balanced		Balanced	Balanced

Training-CV

	CFRNA	COVID-19	OOL	Biobank	Dream
**General Stabl parameters**					
**FDR range (Step)**	0.1 - 1 (0.01)	0.1 - 1 (0.01)	0.1 - 1 (0.01)	0.1 - 1 (0.01)	0.1 - 1 (0.01)
**Lambda name**	C	C	alpha	C	C
**Artificial proportion**	0.5	1	1	1	0.5
**Artificial type**	RP	MX	MX	MX	MX
**Sample fraction**	0.5	0.5	0.5	0.5	0.5
**Replace**	FALSE	FALSE	FALSE	FALSE	FALSE
**Random state**	42	42	42	1	42
**Splitter**	GroupShuffleSplit	RepeatedStratifiedKFold	GroupShuffleSplit	RepeatedStratifiedKFold	GroupShuffleSplit
**Splitter N (splits)**	100	5	100	5	100
**Splitter N (repeats)**		20		20	
**Test size**	0.2		0.2		0.2
**Stabl$_{L}$**					
**N bootstraps**	150	1000	300	500	100
**Lambda grid**	Linear, 0.01, 1, 10	Linear, 0.01, 1, 10	Log, 0, 2, 10	Linear, 0.01, 1, 10	Linear, 0.004, 0.4, 10
**Solver**	liblinear	liblinear		liblinear	liblinear
**Stabl$_{AL}$**					
**N bootstraps**	150	1000	300	500	100
**Lambda grid**	Linear, 0.01, 10, 10	Linear, 0.01, 10, 10	Log, 0, 2, 10	Linear, 0.01, 10, 10	Linear, 0.004, 4, 10
**Solver**	liblinear	liblinear		liblinear	liblinear
**Stabl$_{EN}$**					
**N bootstraps**	50	100	300	100	50
	L1R = 0.5: Log, -2, 1, 5	L1R = 0.5: Log, -2, 1, 5	L1R = 0.5: Log, 1, 2, 5	L1R = 0.5: Log, -2, 0, 5	L1R = 0.2: Log, -3, -2, 5
	L1R = 0.7: Log, -2, 1, 5	L1R = 0.7: Log, -2, 1, 5	L1R = 0.7: Log, 0.5, 2, 5	L1R = 0.7: Log, -2, 0, 5	L1R = 0.5: Log, -3, -2, 5
**Lambda grid**	L1R = 0.9, Log, -2, 1, 5	L1R = 0.9, Log, -2, 1, 5	L1R = 0.9, Log, 0.5, 2, 5	L1R = 0.9, Log, -2, 0, 5	L1R = 0.8, Log, -3, -2, 5
**Solver**	saga	saga		saga	saga
**BaseSRMs**					
**Splitter Type**	RepeatedStratifiedKFold	RepeatedStratifiedKFold	RepeatedStratifiedKFold	RepeatedStratifiedKFold	RepeatedStratifiedKFold
**Splitter n (splits)**	5	5	5	5	5
**Splitter n (repeats)**	5	5	5	5	5
**random state**	42	42	42	42	42
**Lambda grid (Lasso)**	Log, -2, 2, 30	Log, -2, 2, 30	Log, -2, 2, 30	Log, -2, 2, 30	Log, -3, 0, 30
**Lambda grid (ALasso)**	Log, -2, 2, 20	Log, -2, 2, 20	Log, -2, 2, 30	Log, -2, 2, 30	Log, -3, 0, 30
**Lamdba grid (EN)**	Log, -2, 1, 5	Log, -2, 1, 10	Log, -2, 2, 10	Log, -2, 1, 10	Log, -2, 1, 10
**L1 Ratio (L1R)**	0.5, 0.7, and 0.9	0.5, 0.7, and 0.9	0.5, 0.7, and 0.9	0.5, 0.7, and 0.9	Linear, 0.2, 0.8, 3

Training-Validation

	CFRNA	COVID-19	OOL	Biobank	Dream
**General Stabl parameters**					
**FDR range (Step)**	0.1 - 1 (0.01)	0.1 - 1 (0.01)	0.1 - 1 (0.01)	0.1 - 1 (0.01)	0.1 - 1 (0.01)
**Lambda name**	C	C	alpha	C	C
**Artificial proportion**	1	1	1	1	0.5
**Artificial type**	RP	MX	MX	MX	MX
**Sample fraction**	0.5	0.5	0.5	0.5	0.5
**Replace**	FALSE	FALSE	FALSE	FALSE	FALSE
**Random state**	42	42	42	1	42
**Stabl$_{L}$**					
**N bootstraps**	300	5000	2000	5000	250
**Lambda grid**	Linear, 0.01, 1, 10	Linear, 0.01, 1, 30	Log, 0, 2, 30	Linear, 0.01, 1, 30	Linear, 0.004, 0.4, 30
**Solver**	liblinear	liblinear		liblinear	liblinear
**Stabl$_{AL}$**					
**N bootstraps**	300	5000	2000	5000	250
**Lambda grid**	Linear, 0.01, 10, 10	Linear, 0.01, 10, 30	Log, 0, 2, 30	Linear, 0.01, 10, 30	Linear, 0.004, 4, 30
**Solver**	liblinear	liblinear		liblinear	liblinear
**Stabl$_{EN}$**					
**N bootstraps**	100	200	2000	1000	50
	L1R = 0.5: Log, -2, 1, 5	L1R = 0.5: Log, -2, 1, 10	L1R = 0.5: Log, 1, 2, 10	L1R = 0.5: Log, -2, 0, 10	L1R = 0.2: Log, -3, -1, 5
	L1R = 0.7: Log, -2, 1, 5	L1R = 0.7: Log, -2, 1, 10	L1R = 0.7: Log, 0.5, 2, 10	L1R = 0.7: Log, -2, 0, 10	L1R = 0.5: Log, -3, -1, 5
**Lambda grid**	L1R = 0.9, Log, -2, 1, 5	L1R = 0.9, Log, -2, 1, 10	L1R = 0.9, Log, 0.5, 2, 10	L1R = 0.9, Log, -2, 0, 10	L1R = 0.8, Log, -3, -1, 5
**Solver**	saga	saga		saga	saga
**BaseSRMs**					
**Splitter Type**	RepeatedStratifiedKFold	RepeatedStratifiedKFold	RepeatedStratifiedKFold	RepeatedStratifiedKFold	RepeatedStratifiedKFold
**Splitter n (splits)**	5	5	5	5	5
**Splitter n (repeats)**	5	5	5	5	5
**random state**	42	42	42	42	42
**Lambda grid (Lasso)**	Log, -2, 2, 30	Log, -2, 2, 30	Log, -2, 2, 30	Log, -2, 2, 30	Log, -3, 0, 30
**Lambda grid (ALasso)**	Log, -2, 2, 20	Log, -2, 2, 20	Log, -2, 2, 30	Log, -2, 2, 30	Log, -3, 0, 30
**Lamdba grid (EN)**	Log, -2, 1, 10	Log, -2, 1, 10	Log, -2, 2, 10	Log, -2, 1, 10	Log, -3, 0, 10
**L1 Ratio (L1R)**	0.5, 0.7, and 0.9	0.5, 0.7, and 0.9	0.5, 0.7, and 0.9	0.5, 0.7, and 0.9	0.5, 0.7, and 0.9

Supplementary Table S13 | List of hyperparameters used in all clinical case studies. All other parameters were default.
