# Supplementary material for: Discovery of sparse, reliable omic biomarkers with Stabl
Source: Nat Biotechnol. 2024 Jan 2;42(10):1581–93. doi: 10.1038/s41587-023-02033-x (PMC11217152; doi:10.1038/s41587-023-02033-x)
Supplement: Supplementary file 1 [file 41587_2023_2033_MOESM1_ESM.pdf]

nature biotechnology

Article https://doi.org/10.1038/s41587-023-02033-x

Discovery of sparse, reliable omic biomarkers with Stabl

In the format provided by the authors and unedited

	Number of lambdas	Number of bootstraps	Equivalent number of Lasso runs	Time to run (Stabl)
**PE – CFRNA (159 samples, 37184 features)**				
$Stabl_{L}$-RP	30	150	4 500	06 min 50 s
$Stabl_{AL}$-RP	30	150	4 500	10 min 06 s
$Stabl_{EN}$-RP	30	50	1 500	39 min 00 s
**COVID-19 – Proteomics (68, 1463)**				
$Stabl_{L}$-MX	30	1000	30 000	00 min 38 s
$Stabl_{L}$-RP	30	1000	30 000	00 min 38 s
$Stabl_{AL}$-MX	30	1000	30 000	00 min 47 s
$Stabl_{AL}$-RP	30	1000	30 000	00 min 47 s
$Stabl_{EN}$-MX	30	100	3 000	11 min 31 s
$Stabl_{EN}$-RP	30	100	3 000	11 min 27 s
**Onset of Labor – Proteomics (150, 1317)**				
$Stabl_{L}$-MX	30	1000	30 000	01 min 17 s
$Stabl_{L}$-RP	30	1000	30 000	01 min 12 s
$Stabl_{AL}$-MX	30	1000	30 000	01 min 34 s
$Stabl_{AL}$-RP	30	1000	30 000	01 min 25 s
$Stabl_{EN}$-MX	30	1000	30 000	01 min 10 s
$Stabl_{EN}$-RP	30	1000	30 000	01 min 08 s
**Onset of Labor – Metabolomics (150, 3529)**				
$Stabl_{L}$-MX	30	1000	30 000	04 min 11 s
$Stabl_{L}$-RP	30	1000	30 000	03 min 46 s
$Stabl_{AL}$-MX	30	1000	30 000	05 min 24 s
$Stabl_{AL}$-RP	30	1000	30 000	04 min 55 s
$Stabl_{EN}$-MX	30	1000	30 000	03 min 57 s
$Stabl_{EN}$-RP	30	1000	30 000	03 min 22 s
**Onset of Labor – CyTOF (150, 1502)**				
$Stabl_{L}$-MX	30	1000	30 000	01 min 04 s
$Stabl_{L}$-RP	30	1000	30 000	01 min 18 s
$Stabl_{AL}$-MX	30	1000	30 000	01 min 12 s
$Stabl_{AL}$-RP	30	1000	30 000	01 min 26 s
$Stabl_{EN}$-MX	30	1000	30 000	00 min 57 s
$Stabl_{EN}$-RP	30	1000	30 000	00 min 41 s
**Dream – Taxonomy (1569, 3725)**				
$Stabl_{L}$-MX	30	250	7 500	08 min 05 s
$Stabl_{L}$-RP	30	250	7 500	07 min 56 s
$Stabl_{AL}$-MX	30	250	7 500	12 min 35 s
$Stabl_{AL}$-RP	30	250	7 500	10 min 30 s
$Stabl_{EN}$-MX	15	50	750	51 min 19 s
$Stabl_{EN}$-RP	15	50	750	44 min 42 s
**Dream – Phylotype (1569, 5468)**				
$Stabl_{L}$-MX	30	250	7 500	06 min 13 s
$Stabl_{L}$-RP	30	250	7 500	06 min 40 s
$Stabl_{AL}$-MX	30	250	7 500	09 min 03 s
$Stabl_{AL}$-RP	30	250	7 500	08 min 38 s
$Stabl_{EN}$-MX	15	50	750	40 min 37 s
$Stabl_{EN}$-RP	15	50	750	36 min 39 s
**SSI – Proteomics (91, 721)**				
$Stabl_{L}$-MX	30	1000	30 000	00 min 29 s
$Stabl_{L}$-RP	30	1000	30 000	00 min 29 s
$Stabl_{AL}$-MX	30	1000	30 000	00 min 35 s
$Stabl_{AL}$-RP	30	1000	30 000	00 min 35 s
$Stabl_{EN}$-MX	30	1000	30 000	43 min 25 s
$Stabl_{EN}$-RP	30	1000	30 000	37 min 59 s
**SSI – CyTOF (93, 1125)**				
$Stabl_{L}$-MX	30	1000	30 000	00 min 30 s
$Stabl_{L}$-RP	30	1000	30 000	00 min 31 s
$Stabl_{AL}$-MX	30	1000	30 000	00 min 38 s
$Stabl_{AL}$-RP	30	1000	30 000	00 min 38 s
$Stabl_{EN}$-MX	30	1000	30 000	47 min 45 s
$Stabl_{EN}$-RP	30	1000	30 000	47 min 35 s

Supplementary Table S1 | Computation time and complexity for all clinical case studies. All benchmarks were launched using an EC2 instances on AWS (32 CPU, 128 GiB of ram).

Supplementary Table S2 | Benchmarking on synthetic data: comprehensive table of results. A comprehensive table numerically listing the results of all benchmark experiments on synthetic datasets and scenarios ($Stabl_{SRM}$ with four SRMs, tested on regression and classification tasks, three correlation structures, and four data distributions) are reported in a comprehensive and searchable online table. https://github.com/gregbellan/Stabl/blob/main/Extended%20Data%20Table%20S2.xlsx

		Stabl				Late fusion Lasso			
	n	Sparsity	Reliability (FDR)	Reliability (JI)	Predictivity	Sparsity	Reliability (FDR)	Reliability (JI)	Predictivity
	50	5 (1 - 9.8)	1 (0.9 - 1)	0 (0 - 0)	1.3 (1.2 - 1.3)	10.5 (1 - 28.5)	1 (0.8 - 1)	0 (0 - 0)	1.1 (1.1 - 1.2)
	75	31 (16.2 - 47.2)	1 (0.9 - 1)	0 (0 - 0.1)	1.3 (1.2 - 1.4)	8 (0 - 20)	0.9 (0 - 1)	0 (0 - 0)	1.1 (1.1 - 1.2)
	100	48.5 (32.2 - 97.5)	1 (0.9 - 1)	0 (0 - 0)	1.3 (1.3 - 1.6)	15 (1 - 27.5)	0.9 (0.8 - 1)	0 (0 - 0.1)	1.1 (1.1 - 1.1)
**Example 1**	150	29 (17 - 102.2)	0.9 (0.8 - 1)	0 (0 - 0.1)	1.2 (1.2 - 1.4)	30.5 (15.2 - 43.8)	0.9 (0.8 - 1)	0.1 (0 - 0.1)	1.1 (1.1 - 1.1)
	200	26 (18 - 127.2)	0.9 (0.9 - 1)	0 (0 - 0.1)	1.2 (1.2 - 1.4)	52.5 (40.2 - 64)	0.9 (0.8 - 0.9)	0.1 (0.1 - 0.1)	1.1 (1 - 1.1)
**Omic 1 (500,40)**	300	33.5 (20.2 - 135)	0.8 (0.8 - 1)	0.1 (0 - 0.1)	1.1 (1 - 1.2)	80.5 (64.8 - 96.5)	0.8 (0.8 - 0.9)	0.1 (0.1 - 0.1)	1 (1 - 1)
**Omic 2 (500,5)**	400	27.5 (19.5 - 42.5)	0.8 (0.7 - 0.9)	0.1 (0.1 - 0.2)	1 (1 - 1.1)	98.5 (78 - 123)	0.9 (0.8 - 0.9)	0.1 (0.1 - 0.2)	0.9 (0.9 - 1)
**Omic 3 (500,5)**	600	28.5 (25 - 35.5)	0.7 (0.6 - 0.7)	0.2 (0.2 - 0.2)	0.9 (0.8 - 0.9)	106.5 (93.8 - 136.5)	0.8 (0.8 - 0.9)	0.1 (0.1 - 0.2)	0.9 (0.9 - 0.9)
	800	32 (29 - 39.5)	0.6 (0.6 - 0.7)	0.3 (0.2 - 0.3)	0.8 (0.8 - 0.8)	133 (111 - 151.8)	0.9 (0.8 - 0.9)	0.1 (0.1 - 0.2)	0.9 (0.9 - 0.9)
	1000	31.5 (28.2 - 39)	0.6 (0.5 - 0.7)	0.3 (0.3 - 0.3)	0.8 (0.7 - 0.8)	126.5 (109.5 - 137.5)	0.8 (0.8 - 0.9)	0.2 (0.1 - 0.2)	0.9 (0.9 - 0.9)
	50	4 (2 - 10)	1 (0.9 - 1)	0 (0 - 0)	1.3 (1.2 - 1.4)	10.5 (1 - 25.8)	1 (0.8 - 1)	0 (0 - 0)	1.1 (1.1 - 1.2)
	75	37 (23.2 - 50.8)	1 (0.9 - 1)	0 (0 - 0)	1.3 (1.3 - 1.5)	11 (3.2 - 31.2)	0.9 (0.9 - 1)	0 (0 - 0)	1.1 (1.1 - 1.2)
	100	43.5 (24.8 - 95.5)	0.9 (0.9 - 1)	0 (0 - 0.1)	1.3 (1.2 - 1.6)	17 (4.5 - 28)	0.9 (0.9 - 1)	0 (0 - 0)	1.1 (1.1 - 1.1)
**Example 2**	150	28 (15 - 51.8)	0.9 (0.9 - 0.9)	0.1 (0 - 0.1)	1.2 (1.2 - 1.3)	21 (10.2 - 38.5)	0.9 (0.8 - 0.9)	0 (0 - 0.1)	1.1 (1.1 - 1.1)
	200	21 (15.5 - 44.8)	0.9 (0.8 - 0.9)	0.1 (0 - 0.1)	1.2 (1.1 - 1.2)	35 (11 - 49.8)	0.9 (0.8 - 0.9)	0.1 (0.1 - 0.1)	1.1 (1.1 - 1.1)
**Omic 1 (500,20)**	300	23 (16 - 33)	0.8 (0.7 - 0.9)	0.1 (0.1 - 0.2)	1.1 (1 - 1.1)	65 (42.2 - 83.8)	0.9 (0.8 - 0.9)	0.1 (0.1 - 0.1)	1.1 (1.1 - 1.1)
**Omic 2 (500,15)**	400	17 (15 - 22.8)	0.7 (0.6 - 0.8)	0.2 (0.1 - 0.2)	1 (0.9 - 1)	89 (75.2 - 106.8)	0.9 (0.8 - 0.9)	0.1 (0.1 - 0.1)	1.1 (1 - 1.1)
**Omic 3 (500,15)**	600	21 (19 - 24.8)	0.6 (0.6 - 0.7)	0.2 (0.2 - 0.2)	0.9 (0.8 - 0.9)	115.5 (86.5 - 138.2)	0.9 (0.8 - 0.9)	0.1 (0.1 - 0.1)	1 (1 - 1)
	800	24 (22.2 - 27)	0.6 (0.6 - 0.6)	0.3 (0.2 - 0.3)	0.8 (0.8 - 0.8)	112.5 (101.2 - 137.8)	0.8 (0.8 - 0.9)	0.1 (0.1 - 0.2)	1 (1 - 1)
	1000	26 (23 - 28)	0.6 (0.5 - 0.6)	0.3 (0.3 - 0.3)	0.8 (0.8 - 0.8)	125.5 (109.8 - 147.2)	0.9 (0.8 - 0.9)	0.1 (0.1 - 0.2)	1 (1 - 1)
	50	15 (8 - 27.2)	1 (0.9 - 1)	0 (0 - 0)	1.3 (1.3 - 1.4)	4 (1.2 - 20.2)	1 (0.9 - 1)	0 (0 - 0)	1.1 (1.1 - 1.2)
	75	26 (10.8 - 43.8)	1 (0.9 - 1)	0 (0 - 0)	1.3 (1.3 - 1.4)	11.5 (3 - 26.2)	0.9 (0.8 - 1)	0 (0 - 0)	1.1 (1.1 - 1.2)
	100	42.5 (25.2 - 84)	0.9 (0.9 - 1)	0 (0 - 0)	1.3 (1.2 - 1.5)	7 (1 - 23.8)	0.9 (0.6 - 1)	0 (0 - 0)	1.1 (1.1 - 1.1)
**Example 3**	150	23 (16.2 - 53)	0.9 (0.9 - 0.9)	0 (0 - 0.1)	1.2 (1.2 - 1.3)	18.5 (9 - 39.8)	0.9 (0.9 - 0.9)	0 (0 - 0.1)	1.1 (1.1 - 1.1)
	200	21.5 (14.5 - 73)	0.9 (0.8 - 0.9)	0.1 (0 - 0.1)	1.2 (1.1 - 1.2)	28 (12.8 - 53.2)	0.9 (0.8 - 0.9)	0.1 (0.1 - 0.1)	1.1 (1.1 - 1.1)
**Omic 1 (900,30)**	300	21 (17 - 28.8)	0.8 (0.7 - 0.8)	0.1 (0.1 - 0.1)	1.1 (1 - 1.1)	75 (51 - 88)	0.9 (0.9 - 0.9)	0.1 (0.1 - 0.1)	1.1 (1 - 1.1)
**Omic 2 (300,10)**	400	23 (18 - 28)	0.7 (0.6 - 0.8)	0.1 (0.1 - 0.2)	1 (1 - 1)	100.5 (77.2 - 115)	0.9 (0.8 - 0.9)	0.1 (0.1 - 0.1)	1 (1 - 1)
**Omic 3 (300,10)**	600	24 (19.2 - 25.8)	0.6 (0.6 - 0.7)	0.2 (0.2 - 0.2)	0.9 (0.8 - 0.9)	114 (92.5 - 149.8)	0.9 (0.8 - 0.9)	0.1 (0.1 - 0.1)	1 (1 - 1)
	800	26 (22.2 - 32.5)	0.6 (0.6 - 0.6)	0.3 (0.2 - 0.3)	0.8 (0.8 - 0.8)	149.5 (107.8 - 161.8)	0.9 (0.9 - 0.9)	0.1 (0.1 - 0.1)	1 (0.9 - 1)
	1000	27 (25 - 30)	0.6 (0.6 - 0.6)	0.3 (0.2 - 0.3)	0.8 (0.8 - 0.8)	140.5 (126.2 - 149.5)	0.9 (0.9 - 0.9)	0.1 (0.1 - 0.1)	0.9 (0.9 - 0.9)
	50	16.5 (11.2 - 32.5)	0.9 (0.9 - 1)	0 (0 - 0)	1.3 (1.3 - 1.4)	8.5 (3 - 27.5)	0.9 (0.9 - 1)	0 (0 - 0)	1.1 (1.1 - 1.2)
	75	15.5 (10.2 - 45.8)	0.9 (0.9 - 1)	0 (0 - 0.1)	1.3 (1.2 - 1.4)	13.5 (2 - 27.2)	0.9 (0.9 - 1)	0 (0 - 0)	1.1 (1.1 - 1.2)
	100	39 (23.2 - 79)	0.9 (0.9 - 0.9)	0.1 (0 - 0.1)	1.3 (1.2 - 1.5)	11.5 (2.2 - 31)	0.9 (0.7 - 0.9)	0 (0 - 0.1)	1.1 (1.1 - 1.1)
**Example 4**	150	33 (18.2 - 54)	0.9 (0.9 - 0.9)	0.1 (0 - 0.1)	1.2 (1.2 - 1.2)	37 (18 - 60.8)	0.9 (0.9 - 0.9)	0.1 (0 - 0.1)	1.1 (1.1 - 1.1)
	200	28.5 (12.5 - 71.5)	0.8 (0.8 - 0.9)	0.1 (0.1 - 0.1)	1.1 (1.1 - 1.2)	45 (21 - 57.8)	0.9 (0.8 - 0.9)	0.1 (0.1 - 0.1)	1.1 (1.1 - 1.1)
**Omic 1 (900,10)**	300	26.5 (19 - 38)	0.8 (0.7 - 0.9)	0.1 (0.1 - 0.1)	1.1 (1 - 1.1)	65.5 (50.2 - 86.5)	0.9 (0.8 - 0.9)	0.1 (0.1 - 0.1)	1 (1 - 1.1)
**Omic 2 (300,20)**	400	20.5 (18 - 30.2)	0.7 (0.7 - 0.8)	0.1 (0.1 - 0.2)	1 (0.9 - 1)	95 (74.8 - 111.5)	0.9 (0.8 - 0.9)	0.1 (0.1 - 0.1)	1 (1 - 1)
**Omic 3 (300,20)**	600	24 (21.2 - 27.8)	0.6 (0.6 - 0.7)	0.2 (0.2 - 0.2)	0.9 (0.8 - 0.9)	104 (86 - 120.5)	0.9 (0.8 - 0.9)	0.1 (0.1 - 0.2)	1 (1 - 1)
	800	25 (24 - 28.8)	0.6 (0.6 - 0.6)	0.3 (0.2 - 0.3)	0.8 (0.8 - 0.8)	112 (94.2 - 131.8)	0.9 (0.8 - 0.9)	0.1 (0.1 - 0.2)	1 (0.9 - 1)
	1000	28.5 (26 - 30.8)	0.6 (0.5 - 0.6)	0.3 (0.3 - 0.3)	0.8 (0.8 - 0.8)	104 (96.5 - 131)	0.8 (0.8 - 0.9)	0.1 (0.1 - 0.2)	0.9 (0.9 - 0.9)
	50	14 (6.5 - 28.5)	0.9 (0.9 - 1)	0 (0 - 0)	1.4 (1.3 - 1.4)	14.5 (1.2 - 27)	0.9 (0.9 - 1)	0 (0 - 0)	1.1 (1.1 - 1.2)
	75	24 (14 - 51.2)	0.9 (0.9 - 1)	0 (0 - 0.1)	1.3 (1.3 - 1.5)	16.5 (1.2 - 29.5)	0.9 (0.9 - 1)	0 (0 - 0)	1.1 (1.1 - 1.2)
	100	34.5 (21 - 47.8)	0.9 (0.9 - 0.9)	0 (0 - 0.1)	1.3 (1.2 - 1.3)	8 (1.2 - 16.5)	0.9 (0.6 - 1)	0 (0 - 0.1)	1.1 (1.1 - 1.1)
**Example 5**	150	38 (27 - 71.2)	0.9 (0.9 - 0.9)	0.1 (0 - 0.1)	1.2 (1.2 - 1.3)	25 (11.2 - 40)	0.9 (0.9 - 0.9)	0.1 (0 - 0.1)	1.1 (1.1 - 1.1)
	200	26 (19 - 49.2)	0.9 (0.8 - 0.9)	0.1 (0.1 - 0.1)	1.1 (1.1 - 1.2)	39.5 (16.2 - 56.2)	0.9 (0.8 - 0.9)	0.1 (0.1 - 0.1)	1.1 (1.1 - 1.1)
**Omic 1 (1000,20)**	300	24 (19 - 30.2)	0.8 (0.7 - 0.8)	0.1 (0.1 - 0.2)	1 (1 - 1.1)	65 (48 - 90.8)	0.9 (0.8 - 0.9)	0.1 (0.1 - 0.1)	1.1 (1 - 1.1)
**Omic 2 (250,15)**	400	20 (17 - 27.2)	0.7 (0.6 - 0.7)	0.2 (0.1 - 0.2)	1 (0.9 - 1)	79 (62.5 - 94.8)	0.9 (0.8 - 0.9)	0.1 (0.1 - 0.1)	1 (1 - 1.1)
**Omic 3 (250,15)**	600	23 (20 - 25.8)	0.6 (0.6 - 0.7)	0.2 (0.2 - 0.2)	0.9 (0.9 - 0.9)	100.5 (90 - 124.8)	0.9 (0.8 - 0.9)	0.1 (0.1 - 0.1)	1 (1 - 1)
	800	25 (22 - 27)	0.6 (0.6 - 0.6)	0.2 (0.2 - 0.3)	0.8 (0.8 - 0.8)	115 (103.5 - 148.2)	0.9 (0.8 - 0.9)	0.1 (0.1 - 0.2)	1 (1 - 1)
	1000	26 (25 - 29)	0.6 (0.5 - 0.6)	0.3 (0.3 - 0.3)	0.8 (0.8 - 0.8)	117 (108 - 127.5)	0.9 (0.8 - 0.9)	0.1 (0.1 - 0.1)	1 (1 - 1)

Supplementary Table S3 | Stabl$_L$'s performance on synthetic multi-omic data compared to Lasso. Sparsity ($|\hat{S}|$), reliability (FDR and JI), and predictivity (RMSE) of Stabl$_L$ and late fusion Lasso are represented as median (IQR) for five examples of synthetic triple-omic datasets with 1450 uninformative and 50 informative features each (1500 features total).

Case study	PE	COVID	OOL			SSI		Dream	
Omic	CFRNA	Proteomics	Proteomics	Metabolomics	CyTOF	Proteomics	CyTOF	Taxonomy	Phylotype
% Corr. > 0.7	<1%	8%	<1%	<1%	3%	1%	2%	18%	5%
% Corr. > 0.5	<1%	16%	2%	2%	7%	3%	7%	35%	11%
% Corr. > 0.2	13%	46%	20%	16%	35%	24%	37%	69%	41%

Supplementary Table S4 | Correlation structure of all clinical case study datasets.

	Predictivity			Sparsity			
	SRM	StablSRM-MX	StablSRM-RP	SRM	StablSRM-MX	StablSRM-RP	p-value SRM vs. StablSRM-MX (RP)
**SSI**							
Lasso	0.722 [0.580, 0.838]	0.818 [0.713, 0.903]	NA	61 [43.25, 83.25]	28.5 [20, 36]	NA	< 1e-16
ALasso	0.757 [0.631, 0.876]	0.804 [0.703, 0.890]	NA	33 [25, 46]	24 [19, 30]	NA	3.03E-06
EN	0.752 [0.630, 0.860]	0.779 [0.677, 0.882]	NA	304.5 [186, 449.25]	30.5 [21.75, 48]	NA	< 1e-16
**COVID (Training cohort)**							
Lasso	0.858 [0.745, 0.952]	0.834 [0.718, 0.920]	0.847 [0.736, 0.935]	17.5 [8, 107.25]	9 [4, 17]	7 [4.75, 13]	1.84E-07 (4e-10)
ALasso	0.863 [0.750, 0.948]	0.763 [0.638, 0.887]	NA	7.5 [3, 18.25]	6 [3, 8]	NA	6.89E-05
EN	0.878 [0.771, 0.954]	0.865 [0.767, 0.951]	NA	473 [22.25, 545]	16.5 [6, 63]	NA	3.89E-14
**COVID (Validation cohort)**							
Lasso	0.759 [0.708, 0.806]	0.749 [0.704, 0.790]	0.748 [0.706, 0.789]				
ALasso	0.741 [0.690, 0.788]	0.758 [0.719, 0.802]	NA				
EN	0.765 [0.715, 0.811]	0.734 [0.678, 0.785]	NA				
**PE**							
Lasso	0.842 [0.781, 0.902]	NA	0.828 [0.759, 0.892]	251.5 [150.25, 358.25]	NA	11 [8, 17]	< 1e-16
ALasso	0.833 [0.761, 0.895]	NA	0.799 [0.730, 0.870]	57 [38.5, 87.5]	NA	7 [5, 11]	< 1e-16
EN	0.830 [0.759, 0.893]	NA	0.859 [0.780, 0.927]	1576 [290.5, 1690.25]	NA	26 [15.5, 38]	< 1e-16
**Dream**							
Lasso	0.713 [0.686, 0.738]	0.687 [0.658, 0.716]	0.685 [0.663, 0.707]	638 [605.75, 675.25]	16 [13.75, 22]	28 [23.25, 32]	< 1e-16 (< 1e-16)
ALasso	0.710 [0.682, 0.738]	0.647 [0.617, 0.675]	NA	367 [336, 413.25]	10 [8, 13]	NA	< 1e-16
EN	0.729 [0.703, 0.754]	0.685 [0.659, 0.713]	NA	3470 [3105.25, 3757]	25 [20, 36.75]	NA	< 1e-16
**OOL (Training cohort)**							
Lasso	14.659 [11.998, 17.823]	15.033 [11.541, 19.063]	14.949 [11.955, 18.351]	194.5 [172, 218.25]	25 [20.75, 38.75]	25 [22 - 29]	< 1e-16 (< 1e-16)
ALasso	14.885 [12.291, 17.814]	14.954 [11.336, 19.139]	NA	133 [106, 162]	25 [21, 36.25]	NA	< 1e-16
EN	14.908 [12.417, 17.408]	17.273 [12.758, 23.093]	NA	470.5 [346, 590.5]	98 [55.75, 196.75]	NA	< 1e-16
**OOL (Validation cohort)**							
Lasso	19.199 [13.933, 24.777]	20.882 [16.567, 24.921]	21.791 [16.703, 26.196]				
ALasso	20.068 [15.404, 24.294]	20.882 [16.950, 24.849]	NA				
EN	19.088 [12.852, 25.003]	24.934 [19.905, 29.488]	NA				

Supplementary Table S5 | Benchmarking on clinical case studies: comprehensive table of results. Significance of sparsity performances was calculated using a two-sided Mann Whitney test.

PE	Predictivity (ROC AUC)	Predictivity (PR AUC)	Sparsity (number of features)
$Stabl_L$-RP	0.829 [0.755, 0.898]	0.850 [0.766, 0.931]	11.000 [7.750, 16.000]
SS (threshold 30%)	0.832 [0.764, 0.894] (p=0.383)	0.862 [0.778, 0.930] (p=0.281)	10.500 [9.000, 12.250] (p=4.683e-01)
SS (threshold 50%)	0.777 [0.694, 0.841] (p=0.014)	0.856 [0.783, 0.913] (p=0.424)	2.000 [2.000, 3.000] (p=6.495e-33)
SS (threshold 80%)	0.464 [0.432, 0.486] (p=0.0)	0.586 [0.507, 0.667] (p=0.0)	0.000 [0.000, 0.250] (p=3.266e-36)

COVID-19 (Training cohort)	Predictivity (ROC AUC)	Predictivity (PR AUC)	Sparsity (number of features)
$Stabl_L$-RP	0.847 [0.736, 0.935]	0.736 [0.543, 0.918]	7.000 [4.750, 13.000]
SS (threshold 30%)	0.851 [0.746, 0.939] (p=0.463)	0.739 [0.577, 0.923] (p=0.488)	3.000 [2.000, 4.000] (p=4.864e-18)
SS (threshold 50%)	0.491 [0.442, 0.532] (p=0.0)	0.364 [0.254, 0.471] (p=0.0)	0.000 [0.000, 1.000] (p=1.026e-35)
SS (threshold 80%)	0.500 [0.500, 0.500] (p=0.0)	0.368 [0.250, 0.485] (p=0.0)	0.000 [0.000, 0.000] (p=5.108e-39)

COVID-19 (Validation cohort)	Predictivity (ROC AUC)	Predictivity (PR AUC)
$Stabl_L$-RP	0.748 [0.706, 0.789]	0.932 [0.912, 0.952]
SS (threshold 30%)	0.778 [0.734, 0.820] (p=0.056)	0.946 [0.930, 0.962] (p=0.011)
SS (threshold 50%)	0.722 [0.670, 0.768] (p=0.252)	0.925 [0.901, 0.947] (p=0.289)
SS (threshold 80%)	0.500 [0.500, 0.500] (p=0.0)	0.841 [0.814, 0.867] (p=0.0)

Supplementary Table S6 | $Stabl_L$'s performance on single-omic datasets compared to SS. Significance of sparsity performances was calculated using a two-sided Mann Whitney test, significance of predictivity performances was calculated using permutation testing.

Features	Coefficients
CDK10	0.11
MT-TM	-1.34
MT-TH	2.43
HNRNPA3P6	-0.81
RPL34P34	-0.71
YWHAQP5	0.46
RPL23AP7	-1.45
GOLGA5P1	-0.76
MTRNR2L8	0.01

Supplementary Table S7 | Features selected by Stabl$_L$ for clinical case study 1: PE.

Features	Coefficients
CCL20	1.74
CRTAC1	-0.66
MDGA1	-2.03
MZB1	2.93

Supplementary Table S8 | Features selected by Stabl$_L$ for clinical case study 2: COVID-19.

Features	Coefficients
Granulocytes_S6_unstim	-3.18
Granulocytes_CREB_GMCSF	-1.65
Granulocytes_STAT1_IFNa	-2.90
NK_STAT1_IFNa	17.86
CD69negCD56loCD16negNK_STAT1_IFNa	-8.28
CD56hiCD16negNK_MAPKAPK2_IL246	1.12
Bcells	-0.91
CCR5posCCR2posCD4Tem	4.46
NKT	2.59
Granulocytes	0.75
Angiopoietin.2	-1.61
Siglec.6	4.09
Activin.A	4.28
IL.1.R4	2.03
SLPI	2.66
MMP.12	-3.65
PLXB2	-1.42
X147.0663_4.7	0.98
X193.0618_5.3	3.34
X331.2264_8.1	0.80
X335.2226_9.3	-2.79
X349.2371_7.5	2.32
X367.1495_0.8	1.37
X371.1895_10.6	3.14
X441.264_9.5	1.28
X443.208_8.4	7.43
X461.2387_8.7	-1.76

Supplementary Table S9 | Features selected by Stabl$_L$ for clinical case study 3: Time to labor.

Patient characteristic	No Surgical Site Infection, 83% (n=77)	Surgical Site Infection, 17% (n=16)
Age (mean + SD)	59.2 ±13.9	58.8 ± 14.2
Male, % (n)	49 (38)	50 (8)
Ethnicity, black, % (n)	1 (1)	0 (0)
BMI	28.3 ± 6.5	26.6 ± 4.6
**Surgical indication**		
Cancer	58% (45)	25% (4)
Inflammatory bowel disease	6% (5)	12.5% (2)
Other	35% (27)	62.5% (10)
**Type of surgery**		
Colectomy	64% (49)	56% (9)
Small bowels	3% (2)	6% (1)
Other	34% (26)	37% (6)
**Surgical approach**		
Minimally invasive	32% (25)	32% (5)
Open surgery	68% (52)	68% (11)
Operative duration, min, mean±SD	213 ±132	234 ±111
ASA classification, median	3	2.5

Supplementary Table S10 | Clinical information for clinical case study 4: SSI.

Target	Clone	Supplier	Catalog #	Concentration (µg/ml)	Comment
CD235ab	HIR2	Biolegend	306615	1	
CD61	VI-PL2	BD	555752	0.5	
CD45	HI30	Biolegend	304045	1	
CD66	CD66a-B1.1	BD	551354	1	
CD7	M-T701	BD	555359	1	
CD19	HIB19	Biolegend	302247	1	
CD45RA	HI100	Biolegend	304143	1	
CD11b	ICRF44	Biolegend	301337	2	
CD4	RPA-T4	Biolegend	300541	2	
CD8a	RPA-T8	BD	557084	1	
CD11c	Bu15	Biolegend	337221	1	
CD123	6H6	Biolegend	306027	1	
TCRγδ	B1	BD	555715	4	
FceRIα	AER-37 (CRA-1)	Biolegend	334602	0.5	
CD161	HP-3G10	Biolegend	339919	4	
CD33	WM53	Biolegend	303419	2	
CRTH2	BM16	Biolegend	350102	4	
CD16	3G8	Biolegend	302051	2	
CD25	M-A251	Biolegend	356102	2	
CD3	UCHT1	Biolegend	300443	1	
CXCR4	12G5	Biolegend	306502	4	
CD62L	DREG.200	Thermo Fisher	BMS1015	0.5	
CCR2	K036C2	Biolegend	357202	2	
HLA-DR	L243	Biolegend	307651	2	
CD14	M5E2	Biolegend	301843	4	
CD56	NCAM16.2	BD	559043	1	
OLFM4	hOLFM4	Matt Alder (CCHMC)	-	4	Received as a gift
pCREB	87G3	CST	9198	2	Custom synthesis
pSTAT5	C11C5	CST	51879SF	4	
pp38	36/p38	BD	612281	2	Custom synthesis
pSTAT1	14/P-STAT1	BD	612132	1	Custom synthesis
pSTAT3	M9C6	CST	74309SF	4	
pS6	D57.2.2E	CST	4858	2	Custom synthesis
pMAPKAPK2	27B7	CST	3007	1	Custom synthesis
Tbet	4B10	Thermo Fisher	14-5825-82	8	
cPARP	F21-852	BD	552597	2	
FoxP3	PCH101	Thermo Fisher	14-4776-82	10	
IκB	L35A5	CST	4814	8	Custom synthesis
pNFκB	K10-895.12.50	BD	558393	2	
pERK1/2	D13.14.4E	CST	45899SF	2	
pSTAT6	A15137E	Biolegend	686002	2	

Supplementary Table S11 | Antibody panel for clinical case study 4: SSI.

Features	Coefficients
unstim_CD4Trm_Frequency	4.90
unstim_Tregnaive_Frequency	0.84
IL246_Granulocytes_154Sm_STAT3	-0.02
IL246_Th1naive_153Eu_STAT1	1.89
SAMHD1	2.69
ARL11	3.28
LDLR	3.37
RNASET2	1.65
HSPH1	2.22
WWOX	2.82
MTHFD2	0.67
OGG1	-4.60
ITGAV|ITGB3	-2.78
CNTN3	-0.17
CTNNA3	1.35
IL1B	3.68
CCL3	3.69
FCGR1A	1.88
MAPK8	2.49
CSF2	1.16
PPIB	-0.31
BIRC3	-2.88
IL18	1.92
ASAH1	1.93
ALK	3.04
TNFSF10	1.77
RAP2A	0.37
TOPBP1	0.75
IRF6	0.15

Supplementary Table S12 | Features selected by Stabl$_L$ for clinical case study 4: SSI.

	CFRNA	COVID-19	OOL	Biobank	Dream
**Preprocessing**					
**Variance threshold**	0.01	0.01	0.01	0.01	0.01
**Low info filter**	0.2	0.2	0.2	0.2	0.2
**Simple Imputer**	median	median	median	median	median
**StandardScaler**	default	default	default	default	default
**Class weight**	Balanced	Balanced		Balanced	Balanced

Training-CV

**General Stabl parameters**					
**FDR range (Step)**	0.1 - 1 (0.01)	0.1 - 1 (0.01)	0.1 - 1 (0.01)	0.1 - 1 (0.01)	0.1 - 1 (0.01)
**Lambda name**	C	C	alpha	C	C
**Artificial proportion**	0.5	1	1	1	0.5
**Artificial type**	RP	MX	MX	MX	MX
**Sample fraction**	0.5	0.5	0.5	0.5	0.5
**Replace**	FALSE	FALSE	FALSE	FALSE	FALSE
**Random state**	42	42	42	1	42
**Splitter**	GroupShuffleSplit	RepeatedStratifiedKFold	GroupShuffleSplit	RepeatedStratifiedKFold	GroupShuffleSplit
**Splitter N (splits)**	100	5	100	5	100
**Splitter N (repeats)**		20		20	
**Test size**	0.2		0.2		0.2
**Stabl$_{L}$**					
**N bootstraps**	150	1000	300	500	100
**Lambda grid**	Linear, 0.01, 1, 10	Linear, 0.01, 1, 10	Log, 0, 2, 10	Linear, 0.01, 1, 10	Linear, 0.004, 0.4, 10
**Solver**	liblinear	liblinear		liblinear	liblinear
**Stabl$_{AL}$**					
**N bootstraps**	150	1000	300	500	100
**Lambda grid**	Linear, 0.01, 10, 10	Linear, 0.01, 10, 10	Log, 0, 2, 10	Linear, 0.01, 10, 10	Linear, 0.004, 4, 10
**Solver**	liblinear	liblinear		liblinear	liblinear
**Stabl$_{EN}$**					
**N bootstraps**	50	100	300	100	50
**Lambda grid**	L1R = 0.5: Log, -2, 1, 5<br>L1R = 0.7: Log, -2, 1, 5<br>L1R = 0.9, Log, -2, 1, 5	L1R = 0.5: Log, -2, 1, 5<br>L1R = 0.7: Log, -2, 1, 5<br>L1R = 0.9, Log, -2, 1, 5	L1R = 0.5: Log, 1, 2, 5<br>L1R = 0.7: Log, 0.5, 2, 5<br>L1R = 0.9, Log, 0.5, 2, 5	L1R = 0.5: Log, -2, 0, 5<br>L1R = 0.7: Log, -2, 0, 5<br>L1R = 0.9, Log, -2, 0, 5	L1R = 0.2: Log, -3, -2, 5<br>L1R = 0.5: Log, -3, -2, 5<br>L1R = 0.8, Log, -3, -2, 5
**Solver**	saga	saga		saga	saga
**BaseSRMs**					
**Splitter Type**	RepeatedStratifiedKFold	RepeatedStratifiedKFold	RepeatedStratifiedKFold	RepeatedStratifiedKFold	RepeatedStratifiedKFold
**Splitter n (splits)**	5	5	5	5	5
**Splitter n (repeats)**	5	5	5	5	5
**random state**	42	42	42	42	42
**Lambda grid (Lasso)**	Log, -2, 2, 30	Log, -2, 2, 30	Log, -2, 2, 30	Log, -2, 2, 30	Log, -3, 0, 30
**Lambda grid (ALasso)**	Log, -2, 2, 20	Log, -2, 2, 20	Log, -2, 2, 30	Log, -2, 2, 30	Log, -3, 0, 30
**Lamdba grid (EN)**	Log, -2, 1, 5	Log, -2, 1, 10	Log, -2, 2, 10	Log, -2, 1, 10	Log, -2, 1, 10
**L1 Ratio (L1R)**	0.5, 0.7, and 0.9	0.5, 0.7, and 0.9	0.5, 0.7, and 0.9	0.5, 0.7, and 0.9	Linear, 0.2, 0.8, 3

Training-Validation

**General Stabl parameters**					
**FDR range (Step)**	0.1 - 1 (0.01)	0.1 - 1 (0.01)	0.1 - 1 (0.01)	0.1 - 1 (0.01)	0.1 - 1 (0.01)
**Lambda name**	C	C	alpha	C	C
**Artificial proportion**	1	1	1	1	0.5
**Artificial type**	RP	MX	MX	MX	MX
**Sample fraction**	0.5	0.5	0.5	0.5	0.5
**Replace**	FALSE	FALSE	FALSE	FALSE	FALSE
**Random state**	42	42	42	1	42
**Stabl$_{L}$**					
**N bootstraps**	300	5000	2000	5000	250
**Lambda grid**	Linear, 0.01, 1, 10	Linear, 0.01, 1, 30	Log, 0, 2, 30	Linear, 0.01, 1, 30	Linear, 0.004, 0.4, 30
**Solver**	liblinear	liblinear		liblinear	liblinear
**Stabl$_{AL}$**					
**N bootstraps**	300	5000	2000	5000	250
**Lambda grid**	Linear, 0.01, 10, 10	Linear, 0.01, 10, 30	Log, 0, 2, 30	Linear, 0.01, 10, 30	Linear, 0.004, 4, 30
**Solver**	liblinear	liblinear		liblinear	liblinear
**Stabl$_{EN}$**					
**N bootstraps**	100	200	2000	1000	50
**Lambda grid**	L1R = 0.5: Log, -2, 1, 5<br>L1R = 0.7: Log, -2, 1, 5<br>L1R = 0.9, Log, -2, 1, 5	L1R = 0.5: Log, -2, 1, 10<br>L1R = 0.7: Log, -2, 1, 10<br>L1R = 0.9, Log, -2, 1, 10	L1R = 0.5: Log, 1, 2, 10<br>L1R = 0.7: Log, 0.5, 2, 10<br>L1R = 0.9, Log, 0.5, 2, 10	L1R = 0.5: Log, -2, 0, 10<br>L1R = 0.7: Log, -2, 0, 10<br>L1R = 0.9, Log, -2, 0, 10	L1R = 0.2: Log, -3, -1, 5<br>L1R = 0.5: Log, -3, -1, 5<br>L1R = 0.8, Log, -3, -1, 5
**Solver**	saga	saga		saga	saga
**BaseSRMs**					
**Splitter Type**	RepeatedStratifiedKFold	RepeatedStratifiedKFold	RepeatedStratifiedKFold	RepeatedStratifiedKFold	RepeatedStratifiedKFold
**Splitter n (splits)**	5	5	5	5	5
**Splitter n (repeats)**	5	5	5	5	5
**random state**	42	42	42	42	42
**Lambda grid (Lasso)**	Log, -2, 2, 30	Log, -2, 2, 30	Log, -2, 2, 30	Log, -2, 2, 30	Log, -3, 0, 30
**Lambda grid (ALasso)**	Log, -2, 2, 20	Log, -2, 2, 20	Log, -2, 2, 30	Log, -2, 2, 30	Log, -3, 0, 30
**Lamdba grid (EN)**	Log, -2, 1, 10	Log, -2, 1, 10	Log, -2, 2, 10	Log, -2, 1, 10	Log, -3, 0, 10
**L1 Ratio (L1R)**	0.5, 0.7, and 0.9	0.5, 0.7, and 0.9	0.5, 0.7, and 0.9	0.5, 0.7, and 0.9	0.5, 0.7, and 0.9

Supplementary Table S13 | List of hyperparameters used in all clinical case studies. All other parameters were default.
