# Supplementary material for: Discovery of sparse, reliable omic biomarkers with Stabl
Source: Nat Biotechnol. 2024 Jan 2;42(10):1581–93. doi: 10.1038/s41587-023-02033-x (PMC11217152; doi:10.1038/s41587-023-02033-x)
Supplement: Supplementary file 2 [file 41587_2023_2033_MOESM2_ESM.pdf]

nature portfolio

Corresponding author(s): Brice Gaudillière, MD, PhD

Last updated by author(s): 10/10/2023

Reporting Summary

Nature Portfolio wishes to improve the reproducibility of the work that we publish. This form provides structure for consistency and transparency in reporting. For further information on Nature Portfolio policies, see our Editorial Policies and the Editorial Policy Checklist.

Statistics

For all statistical analyses, confirm that the following items are present in the figure legend, table legend, main text, or Methods section.

n/a	Confirmed	
☐	☒	The exact sample size (*n*) for each experimental group/condition, given as a discrete number and unit of measurement
☐	☒	A statement on whether measurements were taken from distinct samples or whether the same sample was measured repeatedly
☐	☒	The statistical test(s) used AND whether they are one- or two-sided<br>*Only common tests should be described solely by name; describe more complex techniques in the Methods section.*
☐	☒	A description of all covariates tested
☐	☒	A description of any assumptions or corrections, such as tests of normality and adjustment for multiple comparisons
☐	☒	A full description of the statistical parameters including central tendency (e.g. means) or other basic estimates (e.g. regression coefficient) AND variation (e.g. standard deviation) or associated estimates of uncertainty (e.g. confidence intervals)
☐	☒	For null hypothesis testing, the test statistic (e.g. *F*, *t*, *r*) with confidence intervals, effect sizes, degrees of freedom and *P* value noted<br>*Give P values as exact values whenever suitable.*
☐	☒	For Bayesian analysis, information on the choice of priors and Markov chain Monte Carlo settings
☒	☐	For hierarchical and complex designs, identification of the appropriate level for tests and full reporting of outcomes
☐	☒	Estimates of effect sizes (e.g. Cohen's *d*, Pearson's *r*), indicating how they were calculated

Our web collection on statistics for biologists contains articles on many of the points above.

Software and code

Policy information about availability of computer code

Data collection	The data collected or used in this study was either available through previous publications, or in the case of the clinical study use-case #4 dataset, collected using the default software for the CyTOF 3.0 Helios instrument (Helios CyTOF Software v7.0.5189. Standard Bio Tools, Inc), and then gated using CellEngine (CellCarta, Montreal, Canada). A detailed gating strategy is provided in supplementary extended data.
Data analysis	The Stabl framework and custom computer code used in this study for the data analysis can be accessed on GitHub (www.github/gregbellan/Stabl) and Zenodo (https://doi.org/10.5281/zenodo.8406758).<br><br>We used:<br>- Python (version from 3.7 up to 3.10) packages: joblib v1.1.0, tqdm v4.64.0, matplotlib v3.5.2, numpy v1.23.1, knockpy v1.2, scikit-learn v1.1.2, seaborn v0.12.0, groupyr v0.3.2, pandas v1.4.2, statsmodels v0.14.0, openpyxl v3.0.7, adjustText v0.8, scipy v1.10.1, julia v0.6.1, osqp v0.6.2<br>- Julia (version 1.9.2) packages: Bigsimr v0.8.7, Distributions v0.25.98, PyCall v1.96.1<br>- Cmake version 3.27.4 (version for mac)

For manuscripts utilizing custom algorithms or software that are central to the research but not yet described in published literature, software must be made available to editors and reviewers. We strongly encourage code deposition in a community repository (e.g. GitHub). See the Nature Portfolio guidelines for submitting code & software for further information.

Data

Policy information about availability of data

All manuscripts must include a data availability statement. This statement should provide the following information, where applicable:

- Accession codes, unique identifiers, or web links for publicly available datasets
- A description of any restrictions on data availability
- For clinical datasets or third party data, please ensure that the statement adheres to our policy

The datasets generated during and/or analyzed during the current study are available on GitHub (https://github.com/gregbellan/Stabl/tree/main/Sample%20Data) and Dryad (https://doi.org/10.5061/dryad.stqjq2c7d.) (Prior to publication: https://datadryad.org/stash/share/phquF4lYp83HUjX7m9ZwMvSRXlNGRGHyFBkJJPFZivs)

Human research participants

Policy information about studies involving human research participants and Sex and Gender in Research.

Reporting on sex and gender

Only biologically-assigned sex was reported, and demographic characteristics for clinical case study 5 are provided in Supplementary Table S10. Frequency matching was performed to ensure a balanced sex percentage in both groups.

Population characteristics

Population characteristics for clinical case studies 1-4 were previously published. Clinical and demographic characteristics for clinical case study 5 are provided in Supplementary Table S10.

Recruitment

Clinical cases studies 1-4 are based on previously published biological and clinical data. All population-relevant characteristics are available in the cited articles. We are presenting the main characteristics here:

Clinical case study 1 (cfRNA): extracted from a discovery and validation cohort:
Discovery: N=33
Controls: N=16 (age: 32.1 ± 4.9; BMI: 22.8 ± 3.3; race: 100% White)
PE: N=17 (age: 31.1 ± 6.3; BMI: 29.4 ± 7.9; race: [53% White, 6% Black, 24% Asian, 17%Other]; GA at onset of PE: 35.8±3.8)
Validation N=16
Controls: N=4 (age: 30.7 ± 4.8; BMI: 23.5 ± 2.5; race: 100% White)
PE: N=12 (age: 32.3 ± 4.5; BMI: 29.4 ± 7.7; race: [42% White, 8% Black, 33% Asian, 17% Other]; GA at onset of PE: 36.6±3.7)

Clinical case study 2 (COVID-19):
Training:
Mild: N=50 (age: 41.5[23, 78], race: [46% male, 24% Asian, 10% Hispanic/Latino, 46% White, 6% Black, 14% NA], diabetes: [14% Yes/pre-diabetic, 68%No, 18% NA])
Moderate: N=21 (age: 45[19, 78], race: [38% male, 10% Asian, 14% Hispanic/Latino, 48% White, 0% Black, 24% NA], diabetes: [10% Yes/pre-diabetic, 71%No, 19% NA])
Severe: N=26 (age: 52.5[29, 78], race: [46.2% male, 8% Asian, 46% Hispanic/Latino, 15% White, 12% Black, 19% NA], diabetes: [35% Yes/pre-diabetic, 54%No, 12% NA])
Validation: All COVID subjects N=306 (n=784 samples); Age: 58 [45, 75], Male: 53%, Race : [54% Hispanic/Latino, 10% Black], diabetes: [40% Yes])

Clinical case study 3 (Time to labor):
Training: N=53 (n=150 samples); age: 33[30, 35]; BMI: 23.5[21, 25.6]; GA: 39.4 [39.8, 40]; Race: [Asian 49%, White 36%, Other 15%]
Validation: N=10 (n=27 samples); age 31 [29, 33]; BMI 24.3[20.7, 25.3]; GA: 39.2 [38.7, 40.3]; Race: [Asian 60%, White 30%, Other 10%]

Clinical case study 4 (Dream challenge): Extracted from the training cohort:
N=1268; Age range [Unknown 54.5%, < 18: 0.3%, 18-28: 17.9%, 28-38: 23.1%, >38: 4.2%]; Race: [American Indian/Alaska Native: 0.5%; Asian: 6.4%; Black/African American: 59.9%, Native Hawaiian/Other Pacific Islanders: 0.2%; White:28.4%; NA: 5%], Delivery: [Term: 67.1%, Pre-term: 32.9%]

The clinical-study use case 5 is a nested case-control study utilizing samples and clinical outcomes collected in patients enrolled in the "Specimen Collection for Evaluation/Prediction of Operative Outcomes at Stanford (IRB-46978). For this study, patients undergoing non-urgent major abdominal colorectal surgery were enrolled between 07/11/2018 and 11/11/2020 at Stanford University Hospital after approval by the Institutional Review Board of Stanford University. Written informed consent was obtained from all study participants. Inclusion criteria were patients over 18 years of age who were willing and able to sign a written consent. Exclusion criteria were a history of inflammatory/autoimmune conditions not related to the indication for colorectal surgery as well as undergoing surgery that did not include resection of the bowel.

Ethics oversight

The clinical case study 5 utilized data from patients enrolled after approval by the Institutional Review Board of Stanford University (IRB 46978).

Note that full information on the approval of the study protocol must also be provided in the manuscript.

Field-specific reporting

Please select the one below that is the best fit for your research. If you are not sure, read the appropriate sections before making your selection.

☒ Life sciences ☐ Behavioural & social sciences ☐ Ecological, evolutionary & environmental sciences

For a reference copy of the document with all sections, see nature.com/documents/nr-reporting-summary-flat.pdf

Life sciences study design

All studies must disclose on these points even when the disclosure is negative.

Sample size	With respect to clinical case study 5, we utilized the methodology outlined in Hanley et al. to determine the minimum required sample size of 80 patients to achieve an expected AUROC of 0.8, with a maximum 95% confidence interval of 0.25, and an expected SSI incidence of 25%. After conducting a frequency-matching procedure, we included a total of 93 patients, which reduced the expected confidence interval range to 0.23.<br>For the other studies, no sample size calculation was performed as the goal was to compare our model performance to existing models previously tested on published or publicly available datasets.
Data exclusions	No data were excluded from the analysis.
Replication	The goal of the study was to evaluate and compare the performance of multivariable statistical models. For each statistical model, the assessed predictive performances for a given outcome were observed neutrally and compared across modeling approaches without classifying results as success nor failure. To ensure reproducibility of the experiments, all statistical analyses were run multiple time. The statistical significance of our experiments was assessed using a Mann Whitney or a Pearson correlation p-value. When comparing models, we assessed the statistical significance using a permutation test. All synthetic benchmarking experiments were replicated 50 times. Benchmarks on real-omic data were performed using a Monte Carlo cross validation with 20 repetitions of a five-fold cross-validation strategy.
Randomization	There was no randomization as we reused previous datasets entirely or partially for clinical studies 1-4. Randomization was not relevant for case #5 due to its case-control design with frequency-matching (Supplementary Table S10).
Blinding	There was no blinding: non-interventional, observational case-control study.

Reporting for specific materials, systems and methods

We require information from authors about some types of materials, experimental systems and methods used in many studies. Here, indicate whether each material, system or method listed is relevant to your study. If you are not sure if a list item applies to your research, read the appropriate section before selecting a response.

Materials & experimental systems

n/a	Involved in the study	
☐	☒	Antibodies
☒	☐	Eukaryotic cell lines
☒	☐	Palaeontology and archaeology
☒	☐	Animals and other organisms
☐	☒	Clinical data
☒	☐	Dual use research of concern

Methods

n/a	Involved in the study	
☒	☐	ChIP-seq
☐	☒	Flow cytometry
☒	☐	MRI-based neuroimaging

Antibodies

Antibodies used	All antibodies used are provided in Supplementary Table S11.
Validation	All antibodies included in the mass cytometry assay are commercially available. For each targeted epitope, the same antibody clone and commercial provider (identified by clone number and catalogue number) is utilized to ensure reproducibility across experiments. Each antibody is validated in-house using positive and negative cell populations for phenotypic markers. For positive controls in the validation of functional (intracellular signaling) antibodies, we use whole blood stimulated with LPS (expected positive signal for pERK1/2, pP38, pMK2, pCREB, pNF-κB and IκB degradation in TLR4-expressing innate immune cell subsets, such as classical monocytes, cMCs), or Interferon alpha (expected positive signal for pSTAT1, 3, 5, 6 in innate and adaptive cells, such as cMCs and CD4+ T cells). Negative control for signaling antibodies are the respective signal measured in the unstimulated blood sample. Validated antibodies are then titrated and utilized at a concentration within the linear range of the titration curve to ensure maximum sensitivity of signal detection and avoid signal saturation effects.

Clinical data

Policy information about clinical studies

All manuscripts should comply with the ICMJE guidelines for publication of clinical research and a completed CONSORT checklist must be included with all submissions.

Clinical trial registration	NA: The clinical case studies 1-4 are based on published material. The clinical-study use case 5 is an observational case-control study (not a randomized control trial).
Study protocol	The study protocol for the case-control study (clinical case study 5) followed the STROBE (rather than CONSORT) checklist as described in the methods section.
Data collection	For clinical case studies 1-4, the data was previously collected and publicly available. For clinical case study 5, patients undergoing non-urgent major abdominal colorectal surgery were enrolled between 07/11/2018 and 11/11/2020 at Stanford University Hospital after approval by the Institutional Review Board of Stanford University (IRB 48298). Written informed consent was obtained from all study participants. Inclusion criteria were patients over 18 years of age who were willing and able to sign a written consent. Exclusion criteria were a history of inflammatory/autoimmune conditions not related to the indication for colorectal surgery as well as undergoing surgery that did not include resection of the bowel.
Outcomes	Primary and secondary outcomes for clinical case studies 1-5 are described in the methods section and provided in Supplementary Table S10.

Flow Cytometry

Plots

Confirm that:

☒ The axis labels state the marker and fluorochrome used (e.g. CD4-FITC).

☒ The axis scales are clearly visible. Include numbers along axes only for bottom left plot of group (a 'group' is an analysis of identical markers).

☒ All plots are contour plots with outliers or pseudocolor plots.

☒ A numerical value for number of cells or percentage (with statistics) is provided.

Methodology

Sample preparation	For clinical case study 5, whole blood samples were either left unstimulated or stimulated with a series of receptor-specific ligands eliciting key intracellular signaling responses implicated in the host's immune response to trauma/injury, including LPS, TF, and a combination of IL-2/4/6. Samples were then fixed using the PROT1 stabilizer buffer (Smart Tube inc, NV) and immediately stored at -80°C for further analysis. On the day of staining, samples were thawed, red blood cells lysed according to the company's protocol (Smart Tube inc, NV), and stained with a multi-parameter mass cytometry antibody panel using a protocol previously described in Gaudillere et al. SciTM, 2014.
Instrument	Samples were analyzed using a CyTOF 3.0 Helios instrument (Standard Bio Tools, Inc).
Software	The Stabl framework and custom computer code used in this study for the data analysis can be accessed on GitHub (www.github/gregbellan/Stabl) and Zenodo (https://doi.org/10.5281/zenodo.8406758).
Cell population abundance	A total of 5E+5-1E+6 cells were collected per sample for further analysis. No cell sorting was performed.
Gating strategy	Gating was performed using the Cellengine software (cellengine.com). A detailed gating strategy is provided in extended data figure 10.

☒ Tick this box to confirm that a figure exemplifying the gating strategy is provided in the Supplementary Information.
